# Reconstructing shifts in vital rates driven by long-term environmental change: a new demographic method based on readily available data

**DOI:** 10.1002/ece3.549

**Published:** 2013-06-07

**Authors:** Edgar J González, Carlos Martorell

**Affiliations:** Departamento de Ecología y Recursos Naturales, Facultad de Ciencias, Universidad Nacional Autónoma de MéxicoCircuito Exterior s/n, Ciudad Universitaria, 04510, México, DF, Mexico

**Keywords:** Environmental drivers, human impacts, integral projection models, population biology, population structure, time series

## Abstract

Frequently, vital rates are driven by directional, long-term environmental changes. Many of these are of great importance, such as land degradation, climate change, and succession. Traditional demographic methods assume a constant or stationary environment, and thus are inappropriate to analyze populations subject to these changes. They also require repeat surveys of the individuals as change unfolds. Methods for reconstructing such lengthy processes are needed. We present a model that, based on a time series of population size structures and densities, reconstructs the impact of directional environmental changes on vital rates. The model uses integral projection models and maximum likelihood to identify the rates that best reconstructs the time series. The procedure was validated with artificial and real data. The former involved simulated species with widely different demographic behaviors. The latter used a chronosequence of populations of an endangered cactus subject to increasing anthropogenic disturbance. In our simulations, the vital rates and their change were always reconstructed accurately. Nevertheless, the model frequently produced alternative results. The use of coarse knowledge of the species' biology (whether vital rates increase or decrease with size or their plausible values) allowed the correct rates to be identified with a 90% success rate. With real data, the model correctly reconstructed the effects of disturbance on vital rates. These effects were previously known from two populations for which demographic data were available. Our procedure seems robust, as the data violated several of the model's assumptions. Thus, time series of size structures and densities contain the necessary information to reconstruct changing vital rates. However, additional biological knowledge may be required to provide reliable results. Because time series of size structures and densities are available for many species or can be rapidly generated, our model can contribute to understand populations that face highly pressing environmental problems.

## Introduction

Understanding the effects of the environment on populations is central to ecology (Heller and Zavaleta 2009; Pereira et al. 2010; Crone et al. 2011). However, many environmental drivers of population change, such as land degradation, climate change, pollutant buildup, ocean acidification, and succession, operate on a long-term, directional basis (Singh 1998; Parr et al. 2003; Kroeker et al. 2010; Wake 2012). The timescales involved make the study of the impact of environmental change on vital rates (survival, growth, and reproduction) impracticable. The correct identification of such impact will allow conservation efforts to be directed more appropriately, to better understand the basis of population change, or even to track the evolutionary changes in life-history traits through time. This calls for specific methods that tackle this problem (Doak and Morris 1999; Pereira et al. 2010; Crone et al. 2011).

Traditional demographic modeling does not provide a solution, as it often assumes that environment change does not occur in a directional fashion (Caswell 2001; Ellner and Rees 2007). Nevertheless, if the environmental driver we are studying changes directionally, population would never reach stability, which is usually the focus of traditional models. Assuming stability in a changing population leads to biased conclusions (Koons et al. 2005). Furthermore, traditional models use repeat surveys of the individuals as input (Caswell 2001). Doing this for the decades or centuries required for environmental change to unfold is impracticable. A substitute, but also costly, approach would be to survey over a representative time period the individuals of a series of populations at different stages of environmental change (Dahlgren and Ehrlén 2011). However, if we are to accomplish global goals such as the assessment of the conservation status and long-term threats for all plant species by 2020 (COP 10 2010), a faster and cheaper alternative to such traditional demographic methods becomes imperative.

A viable approach would be to use time series of static, population-level data, such as population densities and structures, to reconstruct the species vital rates and their change through time as the environment changes. Such datasets have been recorded over several years for different species in the context of forestry, hunting, fisheries, and long-term ecological research (Waters 1999; Hobbie et al. 2003; Parr et al. 2003; Clucas 2011). Also, this kind of data can be rapidly collected for several populations that represent different stages of environmental change, and integrated into a chronosequence (Matthews and Whittaker 1987; Mori et al. 2007). This reconstruction of vital rates from static data has been successfully applied in the context of fisheries stock assessment (e.g., Fournier et al. 1998; Quinn 2003; Maury et al. 2005; Hilborn 2012). However, the translation of these models into an ecological context is not straightforward, as the amount of information and biological knowledge available in fisheries rarely exists for noncommercial species (Quinn 2003). For instance, the available data for most species will usually be sparsely distributed in time, and not surveyed annually as in fisheries. Also, the demographic behavior of the species that ecologists study can be quite complex, as in many species the vital rates depend on size, rather than on age. In plants, for example, organisms having originally different sizes may end up having the same size after 1 year, due to growth, shrinkage, or stasis (Caswell 2001), thus complicating the relationship between size structure and vital rates. Therefore, a model is needed that accommodates these complexities as well as a wide variety of life cycles.

As a time series of static, population-level data does not inform on the fate of individuals, more than one combination of vital rates would be expected to lead to the same series. For instance, a high proportion of seedlings in a population may result from a large fecundity, a low seedling mortality, or impediments to seedling growth. In a model that uses population-level data, Ghosh et al. (2012) envisage this problem. However, as their aim is to forecast population structures, they circumvent the problem by making assumptions on the vital rates that simplify their model but that do not reflect their behavior at the individual level (Ellner 2012). However, if we are interested in correctly reconstructing vital rates, we cannot make such assumptions.

In this article, we develop a model that, based on a time series of population size structures and densities, reconstructs the shifts in vital rates caused by a directionally changing environmental driver. The model was validated with artificially generated data and with data from a threatened cactus subject to long-term human disturbance. We show that, although more than one scenario may be obtained, the correct solution is always provided by the model, and that basic information on the biology of the species is frequently enough to discard alternative solutions.

## The Model

Our model attempts to reconstruct the vital rates (survival, growth, and reproduction) and their change over time based on a time series of size structures and densities. If these rates change as the environmental driver shifts, the structure and density of the population would be expected to evolve accordingly. The model explores a variety of vital rates, seeking the ones that produce the size structures and densities that best resemble the observed time series. A succinct description of the model is presented below. Please refer to Appendix S1 for the full details.

The vital rates of the size-structured population were modeled by means of an integral projection model (IPM; Easterling et al. 2000). An IPM integrates the vital rates into a function *k* known as the kernel. This function establishes the log-sizes *y* that individuals of log-size *x* may reach from time *t* to *t* + 1, as well as the number and sizes of their descendants. The IPM is expressed through the equation


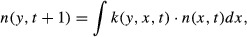
(1)

where *n* is the size structure of the population. Note that, in our model, *k* is a function of time because the vital rates are driven by environmental change.

The kernel comprises the functions associated to the survival probability, *s*, growth, *g*, number of newborns, *f*_1_, and the sizes of these, *f*_2_, which relate as


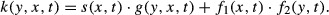
(2)

We used the following simple functions to determine these vital rates:


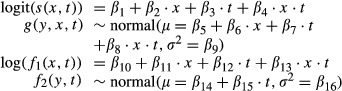
(3)

As can be seen from these equations, the vital rates and their change through time are determined by 16 parameters.

To assess whether any given set of 16 parameter values is able to reproduce the observed time series, we first calculated the vital rates for every year in the period over which environmental change takes place by substituting the parameter values in equations (3). We then calculated the time series of size structures through the iteration of equation (1). To do so, an initial size structure, *n*(*x*,*t*_0_), is required. If no environmental change had occurred before the initial time (i.e., if the environment had remained constant), it would be safe to assume that the population was in its stable state (Caswell 2001). Therefore, in the first iteration of equation (1), we used the stable (asymptotic) size structure associated with the vital rates at the initial observed time. The time series of densities was obtained by integrating the size structures. Finally, we compared these two time series with the observed ones through the composite log-likelihood:


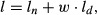
(4)

where *l*_*n*_ and *l*_*d*_ are the log-likelihoods associated with the size structures and with the densities, respectively, and *w* is a weighting factor of the relative importance of the fit of the observed size structures versus that of the observed population densities. Because at each observed point in time there is only one datum for density, but several for size structure, not using a weighting factor could belittle the contribution of density to *l*. The right value for *w* was determined experimentally (see below). The values of the 16 parameters that resulted in the highest *l*-value determined the kernel that best resembled the observed data.

## Model Validation

We performed two validation procedures: one using 10 artificial species for which we simulated time series of size structures and densities, and another using the threatened cactus *Mammillaria dixanthocentron* Backeb. The latter data come from a chronosequence of 11 populations that represent different stages of degradation due to chronic anthropogenic disturbance (CAD). For two of these populations, the vital rates were known from repeat surveys (Ureta and Martorell 2009). Both validations were conducted by comparing the vital rates reconstructed by the model with the known ones.

### Artificial species

We randomly generated the vital rates of 10 different artificial species by: (1) Establishing intervals for the 16 kernel parameters ample enough to accommodate a wide set of possible demographic behaviors: each vital rate could relate with size and time in a positive or negative way, or even not be affected by any of them. (2) Randomly choosing parameter values within these intervals to obtain the kernel for each artificial species. The resulting species had very different demographic behaviors (see Appendix S2 for details and Appendix S3 for a graphical representation of these behaviors).

We then generated a time series of size structures and densities for each species by (3) iterating the kernel over a time interval of 100 years following the procedure described in the Model section. (4) Randomly choosing 10 points in time in the range 1–100 to simulate the likely scenario in which the population is not systematically sampled, and data are available for a few, sparse years. (5) Generating samples of the population at the selected points in time. We assumed that sampling effort was constant, so that sample size was proportional to density. To introduce sampling error, the number of individuals sampled at each of the 10 points in time was simulated from a lognormal distribution with mean equal to the population density at that time. The size of each individual was obtained through a Monte Carlo simulation using the size structure of the year in question as the probability distribution (see Appendix S2).

We used the simulated time series as input to the model. To maximize the likelihood (eq. (4)), we used the Automatic Differentiation Model Builder (ADMB; Fournier et al. 2012; see Appendix S4 for the model code). As it happens when complex functions are optimized, the program may reach different maxima depending on the starting parameter values (Myung 2003). Therefore, we tried 41 different initial sets of values. These were generated by introducing increasingly larger errors (0, 5, 10, 25, and 50%) to the parameter values of the known kernel (see Appendix S2). This allowed us to evaluate how close the initial values had to be from the known ones to correctly reconstruct the kernel. Additionally, to experimentally assess the effect of *w* (eq. (4)), the 41 sets of initial values were run with *w*-values of 0, 1, 10, 100, and 1000. To assess whether the vital rates were correctly reconstructed, the Pearson correlation coefficient between the known and obtained survival, growth, and fecundity functions (*s*, *g*, and *f*_1_ in eq. (2)) was calculated. These three values, together with the correlation between the reconstructed and observed densities (*r*_*d*_), were averaged and a mean coefficient (*r*_*m*_) was calculated and used as an overall measure of fit.

When more than one solution is obtained by an optimization procedure, the researcher needs criteria to discard the incorrect ones. Two different approaches may be used. First, the researcher may resort to goodness-of-fit measures such as the likelihood associated with the size structures (*l*_*n*_ in eq. (4)), or the correlation between the reconstructed and observed densities (*r*_*d*_). Second, the decision may be based on biological knowledge of the species: the researcher may discard solutions that do not match the expected relations between size and vital rates, or the values of the latter are unrealistic.

### Real species: *Mammillaria dixanthocentron*

**Figure 1:**
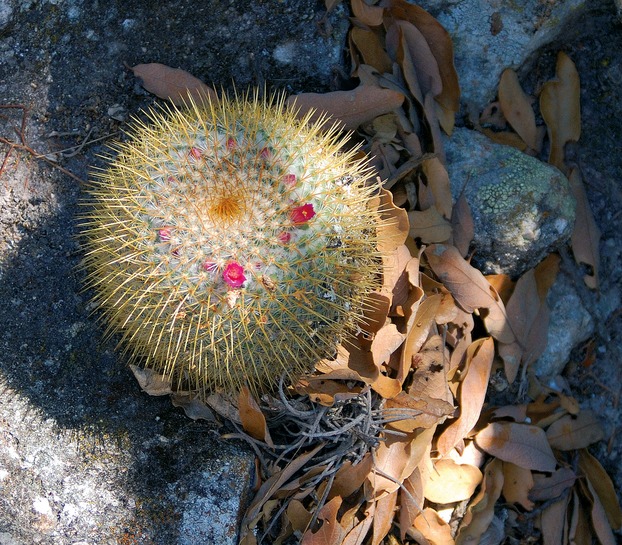
*Mammillaria dixanthocentron* Backeb.

*Mammillaria dixanthocentron* (Fig. 1) is a long-lived globose cactus that grows in tropical and temperate forests in the Mexican states of Puebla and Oaxaca. The region has experienced CAD since pre-Columbian times (McAuliffe et al. 2001), resulting in a mosaic of patches at different stages of degradation. Through demographic models, we know that this species responds negatively to CAD (Ureta and Martorell 2009).

CAD is characterized by a long-term series of frequent, low-intensity disturbance events (Singh 1998). It is a multivariate driver typical of traditional management forms such as extensive grazing, branch cutting, and extraction of nontimber forest products, and has a cumulative effect over time (Singh 1998; Martorell and Peters 2009). Thus, it can be assumed that more disturbed populations have been exposed to CAD for a longer time, and that in the past they resembled the less disturbed populations (i.e., chronosequence assumptions hold; Pickett 1989).

Eleven populations that differed in CAD were studied. In each one, the individuals in a variable number of 50 × 4 m random transects were counted, and their diameter and height measured (see Martorell and Peters 2009 for details). Plant size was defined as the volume of a cylinder. We measured CAD intensity using the Martorell and Peters (2009) index. The rate of increase in disturbance was estimated by measuring CAD at 32 sites (eight of which were *M. dixanthocentron*'s study sites) in 1998 and 2010. We found that CAD increases linearly over time, with an annual rate equal to the mean difference in CAD intensity among these 32 sites divided by 12 years. With this figure, we estimated the times since CAD onset for the 11 study sites.

A modification to the kernel was required to achieve biological realism: as in this species survival probability increases with size, the logistic survival function used for artificial species (*s* in eq. (3)) may estimate zero mortality in the largest individuals. To avoid this, we modified the function by introducing an upper limit different from one that was allowed to change through time. This limit, *s*_max_(*t*) = [1 + exp(*β*_1_ + *β*_2_ · *t*)]^−1^, was multiplied by the original function, raising the number of kernel parameters to 18.

We ran the model using the size structures and densities observed at the 11 sites as input. As before, we set wide parameter intervals, allowing ADMB to select from very contrasting demographic behaviors. However, the intervals of the parameters associated with the effect of size over survival and fecundity were restricted to increase with size as it happens in cacti (Godínez-Álvarez et al. 2003). Despite this restriction, the interaction between size and time (which could not be restricted without biasing the results) could still modify the relation between size and survival/fecundity as time passes by. One hundred starting points were randomly selected within the parameter intervals, and we used the same *w*-values as before. We compared the reconstructed vital rates with the known ones of two *M. dixanthocentron* populations that differ in the intensity of CAD they experience (Ureta and Martorell 2009).

## Results

### Validation with artificial species

**Figure 2:**
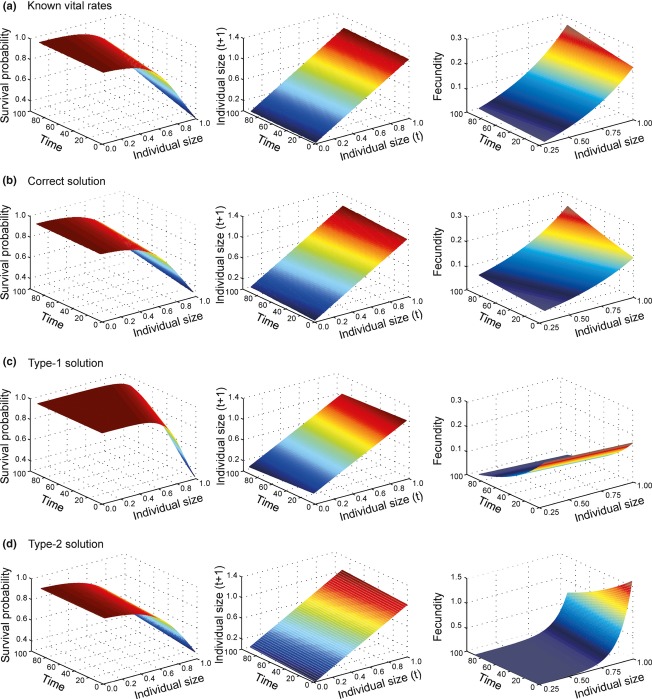
Different kinds of solutions produced by the model. (a) Known vital rates and their change through time for artificial species 3; (b) correct reconstruction; (c) incorrect reconstruction that could be discarded (type-1 solution) because the size-fecundity relation is not expected; (d) erroneous reconstruction that was biologically feasible (type-2 solution) but wrongly estimated that fecundity decreases with time.

In all the artificial species, a solution with an *r*_*m*_ higher than 0.9 was obtained, indicating that the correct vital rates were reconstructed very accurately (see Appendix S3 for a graphical representation of the solutions). However, incorrect solutions were also produced in nine of these species (Table 1). The goodness-of-fit approach to solution selection was ineffective, as neither *l*_*n*_ nor *r*_*d*_ were consistently higher for the correct one (Table 1). The biological knowledge-based approach enabled us to distinguish two types of incorrect results. Type 1: Solutions that grossly misrepresent the species biology. In these cases, the reconstructed relationship between size and vital rates either had an opposite sign to that known for the species, or strong size dependence of these rates was reconstructed when none existed. Most incorrect solutions (94%) were in this category. Type 2: Biologically plausible solutions that could not be discarded. For example, all types of solutions were obtained for the problematic species 3. The correct solution accurately reconstructed the actual vital rates (Fig. 2a and b). A type-1 solution was discarded based on its incorrect reconstruction of fecundity: in this case, the number of seedlings was independent of plant size, while a positive relationship could be expected for the species (Fig. 2c). The correct relationship between size and vital rates was found in a type-2 solution, where fecundity was erroneously reconstructed as decreasing with time (Fig. 2d).

**Table 1:** Solutions obtained by the model for the artificial species

ID	*w* = 0			*w* = 1			*w* = 10			*w* = 100			*w* = 1000		
				
	*r*_*m*_	*r*_*d*_	*l*_*n*_	*r*_*m*_	*r*_*d*_	*l*_*n*_	*r*_*m*_	*r*_*d*_	*l*_*n*_	*r*_*m*_	*r*_*d*_	*l*_*n*_	*r*_*m*_	*r*_*d*_	*l*_*n*_
1	**0.82**	**0.40**	**−51,341**	**0.94**	**0.88**	**−51,342**	0.79	0.89	**−**51,344	0.73	0.90	**−**51,374	0.73	0.99	**−**53,032
				0.78	0.88	**−**51,342									
2	**0.51**	**−0.86**	**−76,294**	0.84	0.97	**−**76,294	0.84	0.97	**−**76,295	0.84	0.97	**−**76,296	**0.95**	**0.91**	**−76,319**
	0.38	**−**0.86	**−**76,294	0.82	0.97	**−**76,303	0.82	0.97	**−**76,306				0.84	0.97	**−**76,311
													0.49	0.98	**−**79,152
3	0.67	0.73	**−**15,763	*0.84*	*0.99*	**−***15,764*	**0.97**	**0.99**	**−15,766**	**0.96**	**1.00**	**−15,828**	**0.99**	**0.99**	**−15,861**
							0.69	0.99	**−**15,779	0.85	1.00	**−**15,815	*0.94*	*1.00*	**−***17,043*
										0.69	0.99	**−**15,797	0.68	0.99	**−**16,113
										0.59	1.00	**−**15,857	0.59	1.00	**−**17,397
													0.44	1.00	**−**17,351
4	0.60	0.15	**−**48,879	**0.98**	**0.92**	**−48,880**	**0.98**	**0.92**	**−48,880**	**0.93**	**0.91**	**−48,952**	*0.93*	*0.92*	**−***49,082*
	**0.51**	**−0.91**	**−48,879**	0.33	**−**0.83	**−**48,888	0.82	0.92	**−**48,881	**0.93**	**0.91**	**−48,955**	*0.75*	*0.97*	**−***51,017*
	0.34	**−**0.88	**−**48,878							*0.91*	*0.92*	**−***48,890*	0.60	0.96	**−**49,438
	0.34	**−**0.90	**−**48,879							0.88	0.92	**−**48,883			
	0.13	**−**0.91	**−**48,880							0.67	0.92	**−**48,986			
										0.56	0.92	**−**48,901			
										0.56	0.92	**−**48,900			
5	**0.99**	**0.99**	**−935,449**	**0.99**	**0.99**	**−935,449**	**0.99**	**0.99**	**−935,449**	**0.99**	**0.99**	**−935,456**	**0.99**	**0.99**	**−935,527**
													**0.95**	**0.95**	**−953,078**
6	**0.90**	**1.00**	**−371,092**	**1.00**	**1.00**	**−371,094**	**1.00**	**1.00**	**−371,097**	**0.99**	**1.00**	**−371,100**	**0.99**	**1.00**	**−371,123**
				0.54	0.90	**−**266,122									
7	0.93	0.95	**−**171,629	**0.99**	**0.98**	**−195,823**	0.93	0.95	**−**171,629	0.94	0.95	**−**171,765	0.57	1.00	**−**168,163
	0.81	0.95	**−**171,731				0.81	0.95	**−**171,732	0.79	0.95	**−**171,820			
	0.55	1.00	**−**160,253				0.55	1.00	**−**160,255	0.55	0.99	**−**160,562			
8	0.78	0.88	**−**84,496	0.84	1.00	**−**84,497	0.87	1.00	**−**84,497	**0.95**	**1.00**	**−84,498**	0.85	1.00	**−**84,519
	0.49	**−**0.40	**−**84,497	0.65	0.88	**−**84,497	0.38	1.00	**−**84,498	0.85	1.00	**−**84,498	0.85	1.00	**−**84,520
	0.40	0.86	**−**84,497	0.36	1.00	**−**84,498				0.39	1.00	**−**84,514.5	0.42	1.00	**−**84,757
	0.11	**−**0.24	**−**84,877												
9	**0.97**	**0.97**	**−610,033**	**0.98**	**0.98**	**−610,035**	0.98	0.97	**−**610,033	**0.98**	**0.98**	**−610,036**	**0.98**	**0.98**	**−610,037**
	0.58	0.72	**−**610,126				0.58	0.72	**−**610,126	0.66	0.97	**−**611,245			
10	**1.00**	**1.00**	**−341,008**	**1.00**	**0.99**	**−368,697**	**1.00**	**0.99**	**−368,698**	0.94	1.00	**−**341,073	0.64	1.00	**−**341,314
	0.64	0.98	**−**341,117	0.63	0.99	**−**368,804				0.64	1.00	**−**341,073	0.59	1.00	**−**341,145

ID, species identifier; *w*, factor weighting the fit of the population densities; *r*_*m*_, mean correlation between the observed and reconstructed vital rates; *r*_*d*_, correlation between the observed and reconstructed population densities; *l*_*n*_, log-likelihood of the reconstructed size structures. Bold, correct solution; roman, biologically unrealistic (type-1) solution; italics, biologically realistic but incorrect (type-2) solution. See Appendix S3 for a graphical representation of these solutions.

When population densities were not considered (*w* = 0), the obtained solutions were often incorrect (Table 1). Low, nonzero *w*-values frequently produced the best results: after discarding type-1 incorrect results, a single correct solution remained in seven species with *w =* 1 (species 1, 4, 5, 6, 7, 9, and 10; Table 1). However, in species 3 with this *w-*value, a type-2 result arose which wrongly reconstructed the change through time of the survival probability (solution 3.2 in Appendix S3); although higher *w*-values produced good results, no criteria could be used to discriminate the wrong reconstructions from the correct ones. For species 2 and 8, no biologically realistic solutions were produced with *w* < 1000 and 100, respectively; with these values, a single realistic solution was obtained. Nevertheless, large *w*-values increased the chances of observing type-2 results in most species (Table 1).

**Figure 3:**
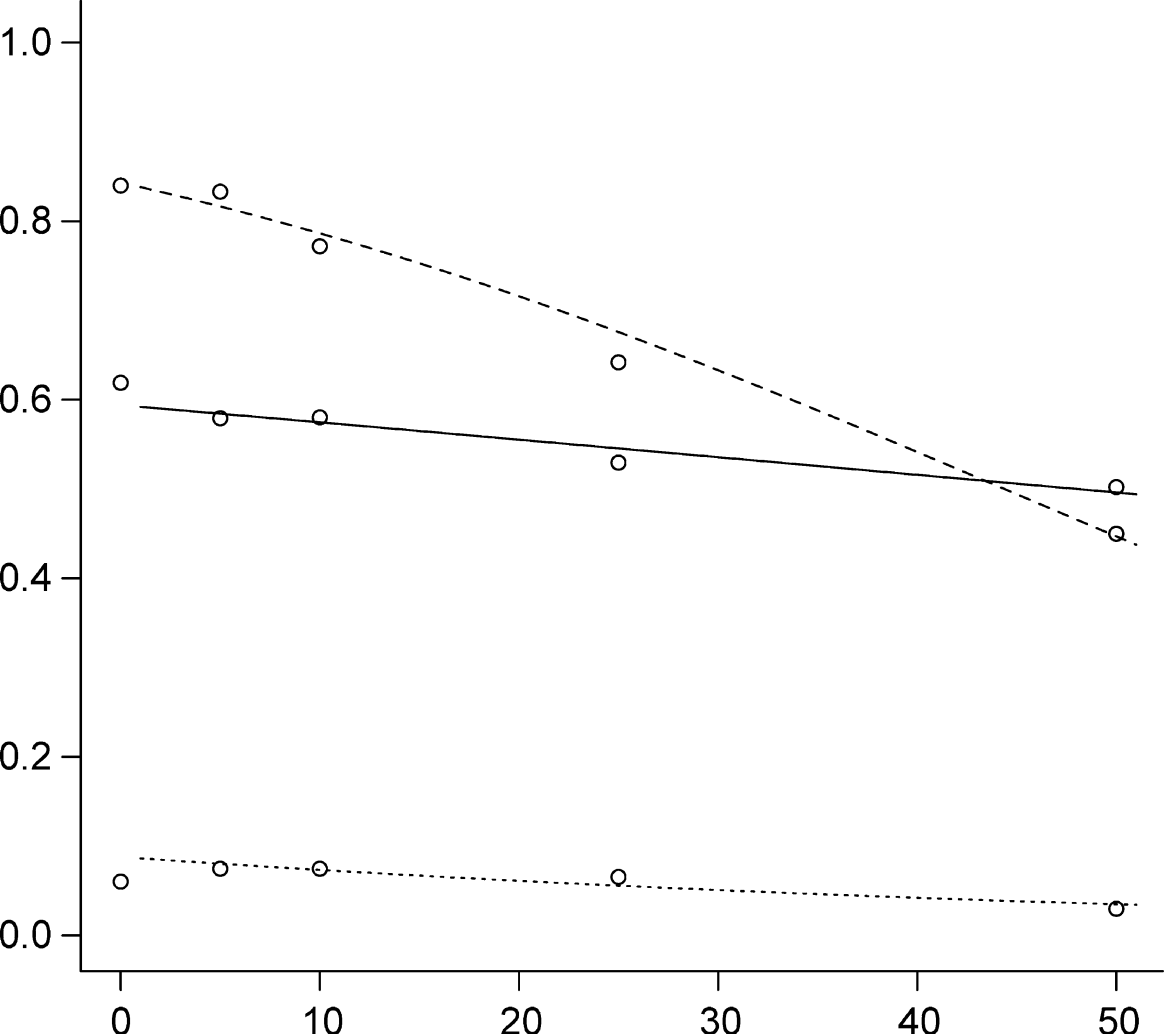
Probabilities of obtaining different results depending on the error in the initial parameters provided to the model. Dashed line: probability of finding versus not finding a solution. Dotted line: ratio of type-2 to type-1 incorrect solutions (see Results). Solid line: ratio of correct to incorrect solutions.

Increasing the error in the vector of starting parameter values diminished the probability of finding the correct solution (binary regression of the ratio of correct:incorrect solutions on the amount of error: *χ*^2^ = 5.51, *P* = 0.018). However, the model was relatively robust, as the probability of finding a correct solution changed from 0.59 if the initial parameter values have no error, to 0.49 if error is large (Fig. 3, solid line). The ratio of type-2:type-1 incorrect solutions did not depend on the amount of error in the initial guess (*χ*^2^ = 3.08, *P* = 0.079; Fig. 3, dotted line). In some cases, ADMB was unable to find any solution at all. This occurred more frequently when poor initial values were provided (*χ*^2^ = 198.79, *P* < 0.001; Fig. 3, dashed line), meaning that more computational time is required to find a solution when less biological knowledge is available.

### Validation with the real species

**Figure 4:**
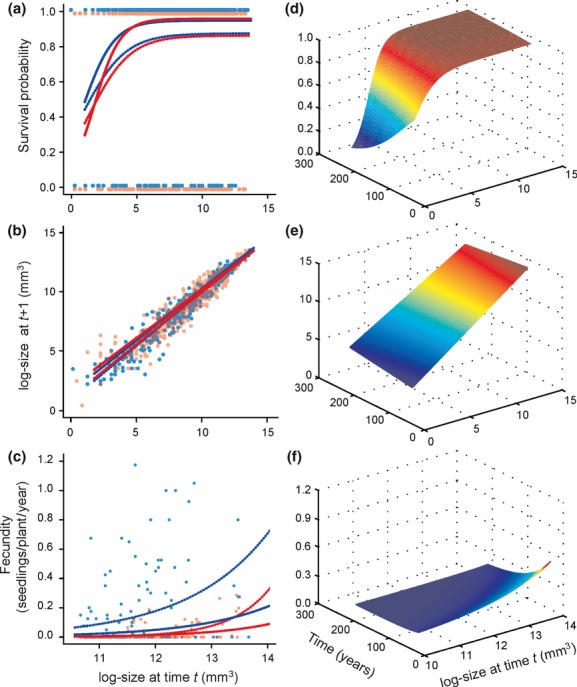
Reconstructed vital rates of *Mammillaria dixanthocentron* and their change due to chronic anthropogenic disturbance. Left panels: comparison of the observed (dotted lines) and reconstructed (solid line) rates at the less (blue) and more (red) disturbed sites. Right panels: reconstructed effect of disturbance on the vital rates along the entire time axis.

The application of the model to *M. dixanthocentron* resulted in a solution that was similar to the vital rates obtained from the two populations (Fig. 4a–c). The 500 ADMB runs (100 starting points with five *w-*values) resulted in the identification of 10 solutions: three with *w =* 0, two with *w =* 1, four with *w =* 100, and one with *w =* 1000. A visual inspection of the reconstructed vital rates allowed to discard nine of these solutions as biologically unrealistic (i.e., type-1 solutions; see Appendix S5): eight presented survival probabilities over 80% for seedlings, which are known to present very low survival probabilities in cacti (Godínez-Álvarez et al. 2003; Ortega-Baes et al. 2010). Another solution was discarded as its fecundity decreased with size, a pattern not expected in plants (Aarssen and Taylor 1992; Weiner et al. 2009). Therefore, only one solution was considered as biologically realistic. This solution reached the limits of seven parameter intervals; however, increasing such intervals did not result in better solutions, but in unrealistic ones such as cacti producing extremely large seedlings (not shown).

The demographic processes associated with the solution were similar to the ones obtained through the repeat survey of individuals (Fig. 4a–c). The model correctly reconstructed the effects of CAD: as disturbance increases, small individuals have lower survival, growth is not affected, and fecundity is reduced regardless of size (Fig. 4d–f). The relationship of size and vital rates was also correctly reconstructed, but the exact values of the latter were not identical to the known ones: the estimated survival of adults (Fig. 4a) and the size of seedlings were larger than the real ones, whereas the fecundity was smaller (Fig. 4c).

## Discussion

The model we present constitutes a viable alternative to model vital rates when repeat surveys of individuals are too costly, labor intensive, or simply impossible to conduct. Its major advantage is the data that it uses as input: if we want to understand the demographic response of a species to long-term environmental change, we only require a series of size structures and densities distributed over time. The model was successful using even very incomplete time series as input. Artificial data showed that the model is able to achieve its goal with a 90% success rate when its assumptions are met. Moreover, real data showed that our procedure is robust to violations of the assumptions. Nevertheless, a rough knowledge of the species biology is essential to discard biologically implausible (type-1) solutions.

### Artificial species: the ideal scenario

The simulation of artificial populations showed that the model successfully reconstructs the sought vital rates under a scenario where all assumptions were fulfilled (see Appendix S1 for a list of model assumptions). The demographic behavior of the artificial species was always correctly reconstructed. Thus, temporally sparse size structures and densities contain the necessary information to reconstruct the correct species' response to long-term environmental change. However, this information was not sufficient, as additional knowledge is required to discriminate among competing solutions, which were obtained in 60% of the cases. Nevertheless, even biological knowledge may sometimes not suffice, as type-2 solutions can also occur.

The question then arises on which solution represents the correct reconstruction. Traditional statistical methods used for this purpose were unsuccessful. From real data, the user can only calculate the correlation coefficient between the observed and estimated densities (*r*_*d*_) and the likelihood associated with the size structures (*l*_*n*_). However, these statistics did not always have the highest values associated with the correct solution, and thus should not (nor those statistics derived from likelihood, such as Akaike's information criterion) be used as a selection criterion. This called for a different selection strategy.

The successful identification of the correct solution can frequently be achieved through an assessment of the results using minimal biological knowledge. This was the case of the real dataset and in 90% of the artificial species (Table 1). Most of the incorrect solutions were discarded because the relationship between size and vital rates was opposite to that expected for the species. This is a very general level of knowledge: for instance, it is known that larger plants have higher fecundities (Aarssen and Taylor 1992; Weiner et al. 2009), and that survival is lower in seedlings than in established plants (Harper and White 1974). Also, the user would expect parameters to take certain values: for example, in mammals litter-size ranges are well known (Haysson et al. 1993). This is indeed a large percentage considering the limited information available to the model and the researcher. The success rate can also be increased if the values used to initiate the likelihood maximization procedure are accurate (Fig. 3). Finally, biological knowledge may be incorporated directly into the analysis through a Bayesian framework, which has been shown to be successful in fisheries models (Punt and Hilborn 1997). However, this requires a much more detailed knowledge of the species life history.

The identification of a correct solution also depends on the factor weighting the fit of the population densities versus that of the size structures (*w* in eq. (4)). In 80% of the cases, the lowest nonzero *w*-value for which a solution was obtained produced the correct reconstruction of the vital rates (Table 1). In the remaining 20%, increasing the value of *w* until a result is obtained increases the probability that a correct solution is identified without further intrusion of the researcher. Such increase will be expected to be necessary when the size structures are noisy or not sufficiently informative about the effect of the environmental driver. For example, in artificial species 2, a correct solution was found with *w =* 1000 but not with lower values (Table 1), probably because size structures did not change appreciably through time (not shown). However, large *w*-values often produce incorrect results, probably because they lead to overfitted solutions where even sampling errors in density are accounted for by the model. Empirically, it seems that keeping the biologically plausible solution with the lowest *w*-value (excluding zero) will usually be the correct choice. It may be a good idea to explore larger *w*-values; this increases (marginally) the probability of reconstructing the vital rates correctly at the cost of rising substantially the chances of finding erroneous type-2 solutions. This sets a trade-off that the researcher should consider depending on the aims of the research.

### Real species: a challenge

Once we showed that the model worked under ideal conditions, we used actual data to evaluate its performance under more realistic circumstances. The model correctly reconstructed the effect of disturbance on the vital rates of the two populations for which demographic data were available. Nevertheless, basic biological knowledge was again required to screen among different solutions.

The reconstructed vital rates were in agreement with what has been previously proposed for cacti sensitive to disturbance: their populations (including *M. dixanthocentron*'s) decline when seedling establishment is reduced by disturbance-driven changes in the environment (Hernández and Godínez-Álvarez 1994; Godínez-Álvarez et al. 2003; Ureta and Martorell 2009). In our case, the reconstructed vital rates showed that CAD negatively affects the number of seedlings that get established and seedling survival. Thus, the reconstruction correctly indicates that CAD diminishes seedling performance.

The results for *M. dixanthocentron* reveal a possible source for deviations in the reconstructed vital rates from the actual ones. Compared with the known rates, the estimated fecundity was smaller and seedling size was larger. It is possible to envisage several scenarios in which approximately the same population structure will result from the balance between the size and number of seedlings produced: given that small seedlings have a very low survival, producing few large seedlings will have a similar effect on population structure than producing many small ones. These solutions are likely to have similar likelihoods, so it would be difficult to favor one over another. However, it seems that this has no impact on the reconstructed change in vital rates, which is the main motivation for using our model.

Previous biological knowledge becomes more relevant when dealing with species for which poor data are available. Some parameters of the reconstructed vital rates were found at the limits of the parameter intervals. Extending the latter produced absurd results, suggesting that our initial selection was sound. Therefore, when data are scarce or violate model assumptions, special attention should be put when establishing parameter intervals or prior distributions as they may largely influence the results.

The system we studied was in fact a challenge for the model, as the data we used as input violated many of its assumptions (Appendix S1). First, time was measured with error: it was estimated from a disturbance index, an approximation to the actual CAD experienced by the population. Furthermore, the assumption that the CAD-time relationship was linear is not necessarily true (Singh 1998). Second, sites should ideally differ only in CAD, representing the different stages experienced by a population as disturbance increases. However, the actual populations differed in several aspects. To mention but one, altitude ranged from 640 to 2500 m a.s.l. Third, climate fluctuations are important in drylands (Schwinning et al. 2004), and thus vital rates are not likely to be deterministic (Fieberg and Ellner 2001; Martorell et al. 2012) as assumed by our model. Furthermore, given that we are working with an endangered species, the samples were relatively small (130 ± 97 individuals per site), limiting the amount of information supplied to the model. Despite these complications, the model was able to reconstruct correctly the change in the vital rates due to CAD, an element pointing toward the model's robustness.

### Advantages and problems

The use of easy-to-obtain information is the main asset of the model. Size structures and densities require only the count and measurement of the individuals in a population at a single point in time. Comparatively, as demographers have experienced, years and resources are required to repeatedly survey the demographic behavior of even a single population. Yet, we acknowledge that ours is a quick-and-dirty procedure (U.S. EPA 2002; Benton 2007), and that the results derived from it have a lower degree of confidence compared with repeat surveys of individuals.

Another asset is that the reconstructed vital rates are free of short-time environmental noise. Repeat surveys of individuals are highly dependent on the particular environmental conditions experienced by the individuals during the year(s) of study. In contrast, size structures and densities reflect a longer time span (Wiegand et al. 2000; Holmes and York 2003), where benign and adverse years have been evened out. In this way, the reconstructed vital rates represent the average behavior of the population as the environment changes.

However, the model will be expected to give poor results in two cases. First, when the species does not respond to environmental change, the model is faced with data that lack the effect of the phenomenon we want to model. If so, only sampling error is fitted, making the reconstruction difficult – if possible at all. In our case, for one artificial species (number 8; Table 1), no single maximum was found probably because its survival probability did not change over time. This situation has also been observed when estimating growth rates of populations that do not respond to environmental change (González et al. 2012).

Second, the reconstruction of the vital rates will be hindered when the functions that describe the behavior of the species are inappropriate. For instance, a simple logistic function for modeling survival in *M. dixanthocentron* caused the largest individuals to become immortal, affecting all the vital rates (not shown). The use of a modified survival function with an asymptote different from one successfully solved the problem, while depicting the biological process more realistically. The specific function employed is probably not so important (González et al. 2012) as long as it captures the key attributes of the vital rate.

### Future directions

Several aspects are in need of evaluation to further increase our confidence on the model. Biologically, providing as much information as possible to the model will probably reduce the number of alternative solutions obtained. For instance, the function relating size and fecundity could be easily known by synchronizing data collection with the reproduction period of the species. This information could serve to discard competing solutions or included directly in the model.

Some mathematical aspects of the model also require elaboration. While the case of *M. dixanthocentron* suggests that the procedure is robust, we are yet unaware of how the violation of different assumptions impacts the results. The researcher may be interested in whether some vital rates are significantly affected by environmental change, for which hypothesis testing and model simplification methods are required. However, we advise that the latter should be avoided, as discarding effects related with a relevant environmental driver when they do exist (type-II error) is, from a conservation biology perspective, more costly than keeping them (Haller 2000).

The modeling of vital rates based on time series of population structures has been developed over the last decades by fisheries researchers (Quinn 2003; Angelini and Moloney 2007; Hilborn 2012), and, in this sense, population ecologists are way behind them. The success of fisheries models is encouraging, showing that procedures based on time series have huge potential.

## Supporting Information

**Appendix S1.** Full description of the model.

**Appendix S2.** Simulation process description.

**Appendix S3.** Vital rates reconstructed for the artificial species.

**Appendix S4.** Model code for ADMB.

**Appendix S5.** Vital rates reconstructed for *Mammillaria dixanthocentron*.
