# Supplementary material for: Reconstructing shifts in vital rates driven by long-term environmental change: a new demographic method based on readily available data
Source: Ecol Evol. 2013 Jun 7;3(7):2273–84. doi: 10.1002/ece3.549 (PMC3728964; doi:10.1002/ece3.549)
Supplement: Supplementary file 1 [file ece30003-2273-SD1.docx]

***Appendix 1***

**Full description of the model**

The model describes the change of the rates of a size-structured population as time passes by. This was done using an integral projection model (IPM; Easterling, Ellner & Dixon 2000) with a time-variant kernel (Ellner & Rees 2007**)**. An IPM is an iterative model relating the vital rates of a population with its size structure. The mathematical description of the vital rates and their change through time is contained in a function *k* known as the kernel. This function establishes the sizes *y* that individuals of size *x* may reach from time *t* to *t*+1, as well as the number and sizes of their descendants. Note that, in our model, *k* is a time-variant because the vital rates are driven by environmental change.

Therefore, an IPM with a time-variant kernel is expressed through the equation

*n*(*y*,*t*+1) = ∫*_X_ k*(*y*,*x*,*t*)⋅*n*(*x*,*t*)*dx*, eqn. A1

where *n* is the size structure of the population and is a continuous probability density function, and *X* is the range of possible individual sizes. Given that we are interested in studying a directional process, *k*(*y*,*x*,*t*) is not ergodic. As a consequence, an IPM with such kernel does not stabilize and no asymptotic properties can be derived from it (Ellner & Rees 2007). Note that the size-structure at time *t*+1, *n*(*y*,*t*+1), will not necessarily integrate to one, but in fact will be the growth rate from time *t* to *t*+1 (*λ_τ_*). After each iteration of equation A1, we standardize the size-structure dividing *n*(*y*,*t*+1) by ∫*_X_ n*(*y*,*t*+1)*dx* to make it a probability density function, which we denote as *ñ*(*y*,*t*+1).

The kernel comprises the functions of survival, *s*(*x*,*t*), and growth, *g*(*y*,*x*,*t*), of existing individuals changing from size *x* to *y* from time *t* to *t*+1, and the functions for the number and sizes of newborns, *f*_1_(*x*,*t*) and *f*_2_(*y*,*x*,*t*), respectively, produced by such individuals. The relation between these functions is given by the equation

*k*(*y*,*x*,*t*) = *s*(*x*,*t*)⋅*g*(*y*,*x*,*t*) + *f*_1_(*x*,*t*)⋅*f*_2_(*y*,*x*,*t*). eqn. A2

We assumed that the size of the parent does not influence the size of its newborn, *i.e. f*_2_(*y*,*x*,*t*) = *f*_2_(*y*,*t*), as this effect is relatively small in the case of many species (Michaels *et al.* 1988). For species with asexual propagation this may not be the case (Méndez & Obeso 1993; Nicholls 2011), but the model can be modified accordingly.

The above functions (*s*, *g*, *f*_1_ and *f*_2_) are usually determined via generalized linear or additive models fitted to observed demographic data (Ellner & Rees 2006; Garcia, Dahlgren & Ehrlén 2011). As the identity of such models is not expected to exert great influence over the reconstructed vital rates (González, Rees & Martorell 2012), we used the following simple functions to determine the vital rates with 16 parameters:

Survival: logit(*s*(*x*,*t*)) = *β*_1_ + *β*_2_⋅*x* + *β*_3_⋅*t* + *β*_4_⋅*x*⋅*t* eqns. A3

Growth: *g*(*y*,*x*,*t*) = normal(*y*; μ = *β*_5_ + *β*_6_⋅*x* + *β*_7_⋅*t* + *β*_8_⋅*x*⋅*t*, σ^2^ = *β*_9_)

Fecundity: log(*f*_1_(*x*,*t*)) = *β*_10_ + *β*_11_⋅*x* + *β*_12_⋅*t* + *β*_13_⋅*x*⋅*t*

Seedling size: *f*_2_(*y*,*t*) = normal(*y*; μ = *β*_14_ + *β*_15_⋅*t*, σ^2^ = *β*_16_)

These functions have been previously used when modeling plant demography using IPMs (Easterling, Ellner & Dixon 2000; Metcalf, Rose & Rees 2003).

To assess the capability of any given set of 16 parameter values to reproduce the observed time series we developed a composite likelihood function. Let us first define the notation associated with the observed data that we will use hereafter: *M* is the number of times the population was sampled, *T*_1_,*T*_2_,…,*T_M_* the times (not necessarily consecutive) at which the populations were sampled, *N*_1_,*N*_2_,…,*N_M_* the number of individuals sampled each time, *X_i_* = {*X_i_*_1_*, X_i_*_2_*, …, X_iNi_*} the set of sizes of all the individuals sampled at time *i*, and *D_i_* the observed population density at time *i*, *i* = 1,…,*M*. Note that we are using capital letters to distinguish the observed data from the functions associated with the model.

For a given set of 16 parameter values, we first obtained the vital rates for each point in time (e.g. year) from *T*_1_ to *T_M_.* This was done by substituting the parameter values in equations A3, assembling the kernel (equation A2) and evaluating this function at times *t = T*_1_,*T*_1_+1,…,*T_M_*. Such evaluations resulted in the vital rates at each point in time. We then calculated the time series of size structures through the iteration of equation A1. To do so, the initial size structure, *n*(*x*,*T*_1_), is required. If no environmental change had occurred before the initial observed time (i.e., if the environment had remained constant), it would be safe to assume that the population was in its stable state (Caswell 2001). Therefore, in the first iteration of equation A1 we used the stable size-structure associated with the vital rates kernel at the initial time; i.e. *n*(*x*,*T*_1_) is the asymptotic size-structure associated with *k*(*y*,*x*,*T*_1_). In case such assumption would not hold (Yearsley 2004), other functions could be used as the initial size structure, such as the one associated with *X*_1_. Therefore, through the iteration of equation A1, we obtain a series of (reconstructed) size structures spanning from *T*_1_ to *T_M_*: *ñ*(*x*,*T*_1_),*ñ*(*x*,*T*_1_+1),…,*ñ*(*x*,*T_M_*). As we were interested in comparing the reconstructed size-structures with the observed data, we retained only those associated with the times of sampling, i.e. we retained *ñ*(*x*,*T*_1_),*ñ*(*x*,*T*_2_),…,*ñ*(*x*,*T_M_*).

The reconstructed population densities resulted from multiplying the growth rates obtained at each iteration by the initial observed density; i.e., for a particular time *t >T*_1_, the modeled density

*d*(*t*) *=* Π*_s_λ_s_D*_1_, eqn. A4

where *s = T*_1_,*T*_1_+1,...,*t* and *λ_s_* = ∫*_X_ n*(*x*,*s*)*dx*.

The composite log-likelihood function we developed combined two log-likelihoods: one comparing the fit of the reconstructed size structures to the observed individual’s sizes and one comparing the fit of the reconstructed densities to the observed ones. The first is defined as the natural logarithm of the probability of observing the sizes of the individuals at the different sampling times given the particular values of the parameters. Such probability is calculated using the probability distributions retained above: *ñ*(*x*,*T*_1_),*ñ*(*x*,*T*_2_),…,*ñ*(*x*,*T_M_*). Mathematically, this is defined as:

*l_n_*(*β*_1_, *β*_2_, …, *β*_16_) = ln(Pr(*X*_1_, …, *X_M_*⎪*β*_1_, *β*_2_, …, *β*_16_))

*=* Σ*_i_* Σ*_j_* ln(*ñ*(*X_ij_*,*T_i_*)), eqn. A5

where *β*_1_, *β*_2_, …, *β*_16_ are the 16 particular values of the parameters, and *X_ij_* is the size of the *j-*th observed individual at the *i-*th sampling point in time.

For the log-likelihood associated with the population densities we did not have a probability distribution associated with each reconstructed density. Therefore, we assumed that *d*(*t*) had a lognormal distribution (denoted lnN). We chose this distribution as density is a positive continuous variable and it would be a reasonable expectation to have ln(*d*(*t*)) normally distributed (Engen & Lande 1996). Again, we define the log-likelihood associated to the fit of the reconstructed densities to the observed ones as the natural logarithm of the probability of observing such densities at the different sampling times given the particular values of the parameters. Such probability is calculated using the lognormal distribution. Mathematically, this is defined as:

*l_d_*(*β*_1_, *β*_2_, …, *β*_16_) = ln(Pr(*D*_1_, *D*_2_, …, *D_M_*⎪*β*_1_, *β*_2_, …, *β*_16_))

*=* Σ*_i_* ln(lnN*_i_*(*d*(*T_i_*))), eqn. A6

where lnN*_i_* is the lognormal distribution associated with *d*(*T_i_*).

Finally, we combined the two log-likelihood functions (equations A5 & A6) into the composite function:

*l*(*p*_1_, *p*_2_, …, *p*_16_) = *l_n_*(*β*_1_, *β*_2_, …, *β*_16_) + *w*⋅*l_d_*(*β*_1_, *β*_2_, …, *β*_16_), eqn. A7

where *w* is a weighting factor of the relative importance on the estimation of the parameters of fitting the observed size-structures *vs.* fitting the observed population densities. This term was introduced because at each point in time (*T_i_)* there is only one datum for density (*D_i_*), but several for size structure ({*X_i_*_1_*, X_i_*_2_*, …, X_iNi_*}), and not using a weight would make density to have a minor contribution to *l*. The right value for *w* can be determined experimentally.

The values of the parameters that maximize equation A7 determine a kernel representing size-structured vital rates and their change through time that best fits the observed size structures and densities.

**Model assumptions**

The main assumption that the model relies on is that, as size-structured vital rates change directionally through time due to a long-term environmental process affecting them, the size structure and density that derive from them change accordingly.

This general assumption can be decomposed into particular easier-to-verify ones. These assumptions can be mandatory or optional. In this latter case, the work needed to change it is indicated in parenthesis.

1. The species recovery time is larger than the recurrence of the environmental process (mandatory).
2. A continuous variable, in our case size, structures the population (mandatory, but cf. Gross *et al.* 2006).
3. The environmental process is chronic and changes through time, be it in intensity or cumulative effect on the population (mandatory).
4. The form of the relation between time and the environmental process is known (linear in our case, but easy to change).
5. The time elapsed between population’s measurements is known without error (mandatory).
6. The form of the relationship between size and each demographic process is known (linear in our case, but easy to change).
7. The form of the relationship between time and each demographic process is known (linear in our case, but easy to change).

Two assumptions relate to the type of data required to use the model:

1. Sample sizes are sufficiently large for the data to contain the information on the effect of the environmental process on the size structure and density (mandatory).
2. Populations are randomly sampled (mandatory).

Another two relate to our decision to use IPMs:

1. Density-independence (easy to change).
2. Deterministic (easy to change will increase complexity and probably reduce confidence on the results, see Ellner & Rees 2007, Ghosh, Gelfand & Clark 2012).

Finally, additional assumptions are particular to the study system used to validate the model with real data:

1. Homogeneity of the environment among sites (violation reduces confidence on the results and other directional factors should be discarded as explanatory variables of the difference between size structures and densities in the different populations).
2. The intensity of the environmental process differs between populations (mandatory).
3. The relationship between chronic anthropogenic disturbance and time is known (linear in our case, but easy to change).
4. Seedling size is parent-size independent (easy to change).
5. Seedling establishment is proportional to flower production (easy to change).
6. The first observed population is in its stable state (easy to change).

**References**

Caswell, H. (2001) *Matrix Population Models: construction, analysis, and interpretation*. Sinauer Associates, Sunderland, MA.

Easterling, M.R., Ellner, S.P. & Dixon, P.M. (2000) Size-specific sensitivity: applying a new structured population model. *Ecology,* **81,** 694-708.

Ellner, S.P. & Rees, M. (2006) Integral projection models for species with complex demography. *The American Naturalist,* **167,** 410-428.

Ellner, S.P. & Rees, M. (2007) Stochastic stable population growth in integral projection models: theory and application. *Journal of Mathematical Biology,* **54,** 227-256.

Engen, S. & Lande, R. (1996) Population dynamic models generating the lognormal species abundance distribution. *Mathematical biosciences,* **132,** 169-183.

Garcia, M.B., Dahlgren, J.P. & Ehrlén, J. (2011) No evidence of senescence in a 300-year-old mountain herb. *Journal of Ecology,* **991,** 1424-1430.

Ghosh, S., Gelfand, A.E. & Clark, J.S. (2012) Inference for size demography from point pattern data using integral projection models. *Journal of Agricultural, Biological, and Environmental Statistics,* **17,** 641-677.

González, E.J., Rees, M. & Martorell, C. (2012) Identifying the demographic processes relevant for species conservation in human-impacted areas: does the model matter? *Oecologia*.

Gross, K., Morris, W.F., Wolosin, M.S. & Doak, D.F. (2006) Modeling vital rates improves estimation of population projection matrices. *Population Ecology,* **48,** 79-89.

Méndez, M. & Obeso, J.R. (1993) Size-dependent reproductive and vegetative allocation in *Arum italicum* (Araceae). *Canadian Journal of Botany,* **71,** 309-314.

Metcalf, J.C., Rose, K.E. & Rees, M. (2003) Evolutionary demography of monocarpic perennials. *Trends in Ecology & Evolution,* **18,** 471-480.

Michaels, H.J., Benner, B., Hartgerink, A.P., Lee, T.D., Rice, S., Willson, M.F. & Bertin, R.I. (1988) Seed size variation: magnitude, distribution, and ecological correlates. *Evolutionary Ecology,* **2,** 157-166.

Nicholls, A.M. (2011) Influences of environmental variability, genetics and plant size on variation in sexual and clonal reproduction and allocation of resources in three wetland plant species. PhD, Cleveland State University.

Yearsley, J.M. (2004) Transient population dynamics and short-term sensitivity analysis of matrix population models. *Ecological Modelling,* **177,** 245-258.
