# Supplementary material for: Reconstructing shifts in vital rates driven by long-term environmental change: a new demographic method based on readily available data
Source: Ecol Evol. 2013 Jun 7;3(7):2273–84. doi: 10.1002/ece3.549 (PMC3728964; doi:10.1002/ece3.549)
Supplement: Supplementary file 2 [file ece30003-2273-SD2.docx]

***Appendix 2***

**Simulation process description**

To generate artificial vital rates representing different species responses to a long-term environmental-change driver, we first had to limit the possible set of rates from where to randomly draw the ones we would use. We did this by setting intervals for the 16 parameters in the model ample enough to incorporate a wide set of possible demographic behaviors. We explored different parameter intervals and chose those that translated into vital rates that were as variable as possible but excluded as many unrealistic ones (see *Parameter Section* of Appendix 4 for these particular intervals). Nonetheless, even the most restricted intervals will contain such rates as certain combinations of values for different parameters can result in unrealistic rates.

We randomly selected parameter values from the intervals and verified if the rates associated with such values were biologically realistic. For a particular set of parameter values, we obtained the associated kernel (*k* in equation A2), and evaluated this function at times *t =* 1,…,100, a span of years over which environmental change will occur, to obtain the vital rates associated with such points in time. As in Appendix 1, we obtained from this kernel a series of (reconstructed) size structures and densities spanning from time 1 to 100 (i.e. *n*(*x*,1),…,*n*(*x*,100) and *d*(1),…,*d*(100)). As explained above, biologically unrealistic rates could be associated with such kernel. Therefore, we verified that the time series fulfilled the following criteria:

1. The population could not increase its density more than a 100 times its original density along the time interval (i.e. *d*(*t*) ≤ 100⋅*d*(1), for every *t* > 1).
2. Starting with 1000 individuals, the population could not arrive to a density of 50 individuals at any point in time to avoid having underrepresented populations (i.e. *d*(*t*) > 50, for every *t* > 1).
3. Standardized size structures (made to integrate to one) had to present a minimum value of 0.0001 in order for the size structures to contain information in all their range (i.e. *n*(*x*,*t*) > 0.0001, for every *t* > 0).

Also, we verified that the mortality of individuals associated with the kernel was larger than 20 % at some individual size and larger than 0.1 % at the largest size at all times in order to avoid having immortal individuals (i.e. *s*(*x*_1_,*t*) < 0.80 for some *x*_1_ in *X* = [0,1], and *s*(*x*_1_,*t*) < 0.999 for every *x* in *X* and every *t* > 0). Using these criteria, we randomly chose 10 biologically realistic kernels, representing the demographic behavior of 10 artificial species. Additionally, we randomly chose 10 points in time in the range 1–100 to simulate the likely scenario in which the population is not systematically sampled, and data are available for a few, sparse years (i.e. *M* = 10 and we chose *T*_1_,…,*T*_10_).

We then generated samples for each artificial species at the selected points in time. To introduce sampling error, the densities at each of the 10 points in time was simulated from a lognormal distribution with mean equal to the population density at that time, *d*(*t_i_*), and variance 0.1. We assumed that sampling effort was constant, so that sample size was proportional to density. Therefore, the new densities, *D_i_*, were used to generate samples of individual’s sizes in the population. For each point in time *T_i_*, a Monte Carlo simulation was performed to obtain a random sample of size *D_i_* of the size-structure *n*(*x*,*T_i_*). The 10 samples obtained in this way, along with densities *D_i_*, represent the type of time series required by the model for the study of a particular species.

For each artificial species, we used its associated time series as input to the model. To maximize the likelihood (equation A7), we used the ADMB. Given that we are dealing with a complex function, the program could reach different maxima depending on the starting parameter values. Therefore, we tried 41 different initial sets of values to explore this function. First, we used as a starting vector the parameter values from which the rates of the species were derived. Then, we obtained additional starting vectors by randomly adding/subtracting to each parameter an error of 5, 10, 25 and 50 % the length of its interval. For each of these percentages, the procedure was repeated 10 times, resulting in 40 random parameter vectors. This allowed us to evaluate how close the initial values had to be from the real ones to correctly reconstruct the kernel.

To assess the effect of the coefficient weighting the relative importance of fitting the population densities vs. that of the size-structures (*w* in equation A7), we ran ADMB under values of 0, 1, 10, 100, and 1000. As can be seen in equation A7, a zero *w*-value corresponds to a model where population densities are not considered in the maximization of the likelihood, whilst increasing *w*-values increase the relevance of fitting the densities in comparison with fitting the size-structures.

After running ADMB with these starting vectors and *w* values (205 runs per species), we usually did not obtained a single reconstruction, but a set of possible ones, which had to be evaluated. First, we evaluated whether these solutions were in fact a single one. To do this, we calculated the distance between them to detect maxima close enough to represent the same rates. We used the weighted distance *d*(*α,β*) = 1/16⋅Σ*_i_*(*α_i_* – *β_i_*)^2^(*max_i_* – *min_i_*)^-2^, where *α* and *β* are two parameter vectors and *max* and *min* are the limits of the parameter intervals. This distance ranges from 0 to 1. Two maxima where considered close to one another when the distance between them was less than 0.05, a value that warranted both maxima to show the same pattern of change of the demographic processes with size and time (not shown).

When two or more solutions were in fact different reconstructions of the vital rates, two different approaches could be used to evaluate them in a realistic scenario. First, we could compare the likelihood associated with the size structures (*l_n_* in equation A5) and the Pearson correlation coefficient between the reconstructed and observed densities (*r_d_*) to perform model selection. These two figures would be the only goodness-of-fit measures that a researcher could compute with a time series of size structures and densities as data. Second, evaluation could be based on relatively basic biological knowledge of the species: the expected relations between vital rates and size.

Additionally, given that for the artificial species we in fact knew their associated kernels, we calculated the goodness-of-fit between the known and reconstructed vital rates via the Pearson correlation coefficient between the known and obtained survival, growth and fecundity functions (*s*, *g*, and *f* in equations A3). These three values, together with *r_d_*, were averaged and a mean coefficient (*r_m_*) was calculated. We would expect that, if *l_n_* and *r_d_* were useful to discriminate among different solutions, they would be positively correlated with *r_m_.*
