# Supplementary material for: Reconstructing shifts in vital rates driven by long-term environmental change: a new demographic method based on readily available data
Source: Ecol Evol. 2013 Jun 7;3(7):2273–84. doi: 10.1002/ece3.549 (PMC3728964; doi:10.1002/ece3.549)

Appendix 3

Vital rates reconstructed for the artificial species

Known survival, growth and fecundity associated to the artificial species (rectangle) and solutions obtained by the model with different values of the coefficient weighting the fitting of the population densities vs. that of the size structures (w in equation 4). r_m is the mean Pearson correlation coefficient between the observed and reconstructed demographies. Bold r_m: correct reconstruction, roman r_m: incorrect reconstruction that could be discarded (type-1 solution), italics r_m: erroneous reconstruction that was biologically feasible but wrongly estimated the change over time of the vital rates (type-2 solution).

Artificial species 1

Known vital rates

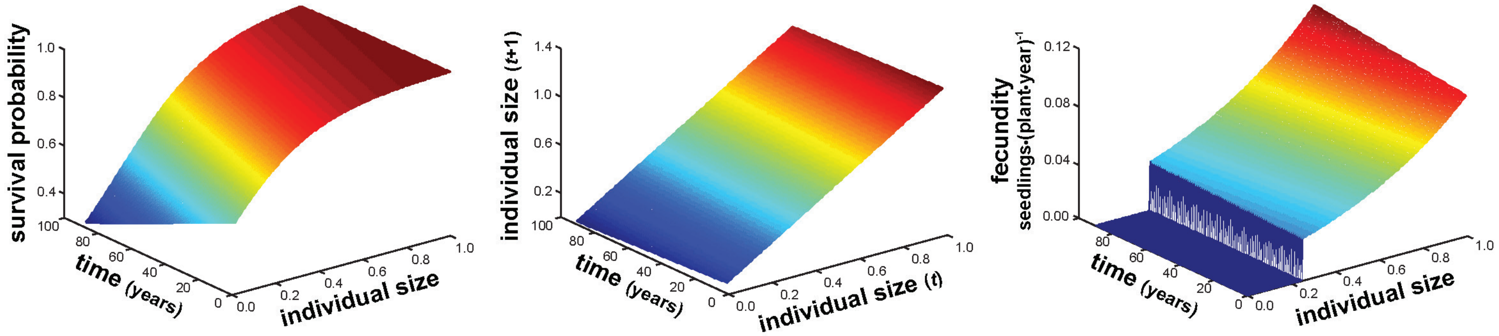

w = 0

r_m = **0.82**

Solution 1.1

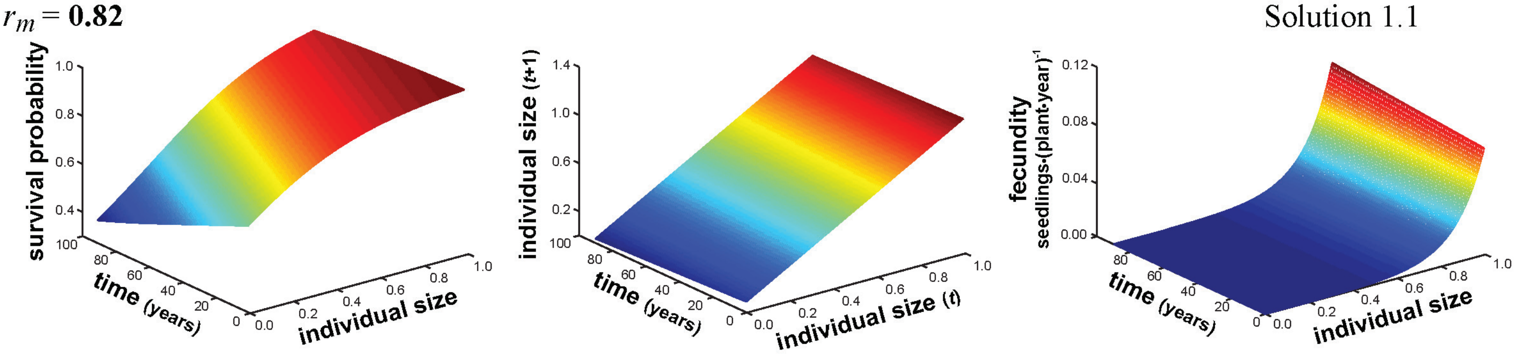

w = 1

r_m = **0.94**

Solution 1.2

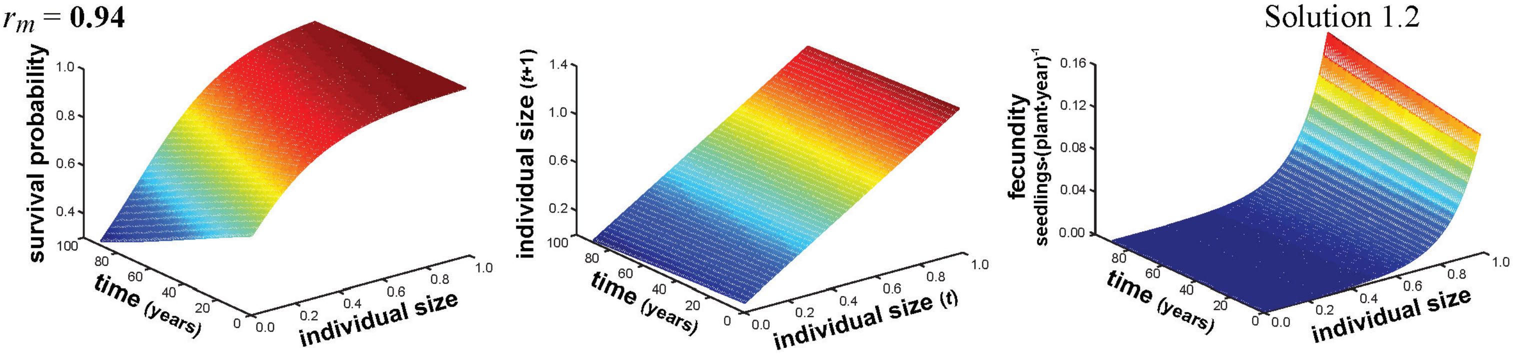

r_m = 0.78

Solution 1.3

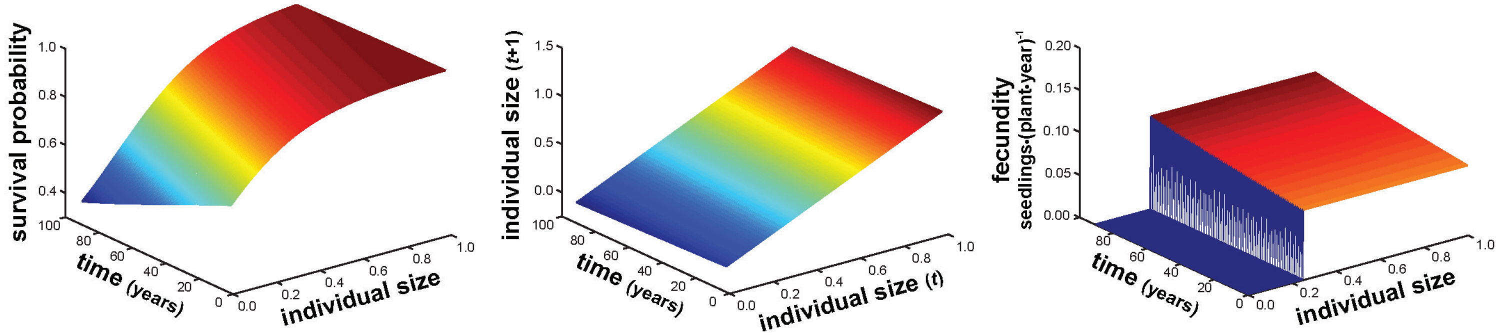

Appendix 3

Vital rates reconstructed for the artificial species

Artificial species 1 (*cont.*)

w = 10

r_m = 0.79

Solution 1.4

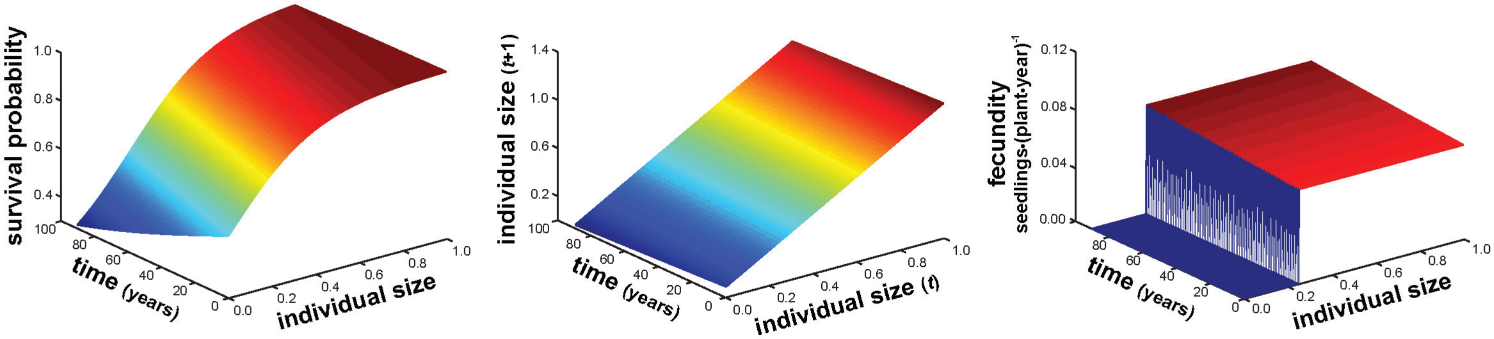

w = 100

r_m = 0.73

Solution 1.5

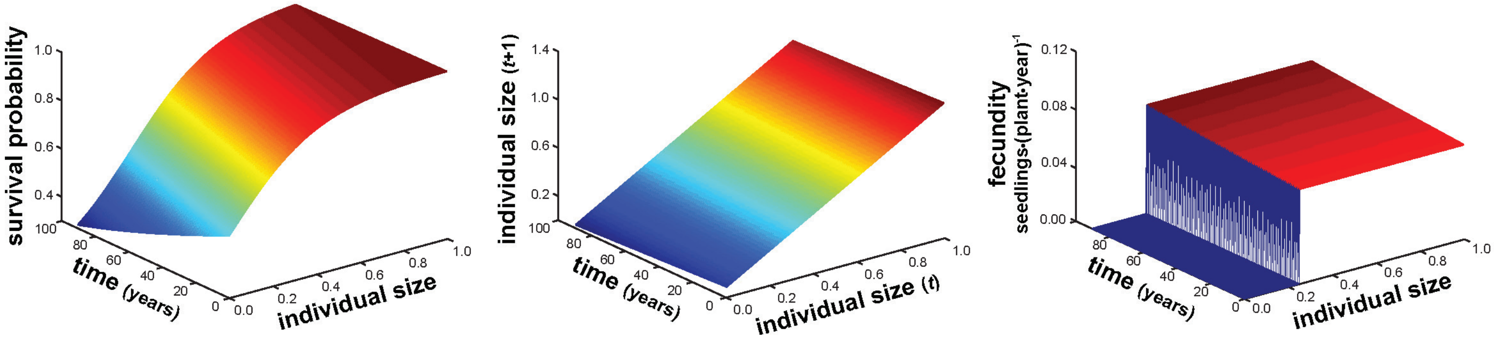

w = 1000

r_m = 0.73

Solution 1.6

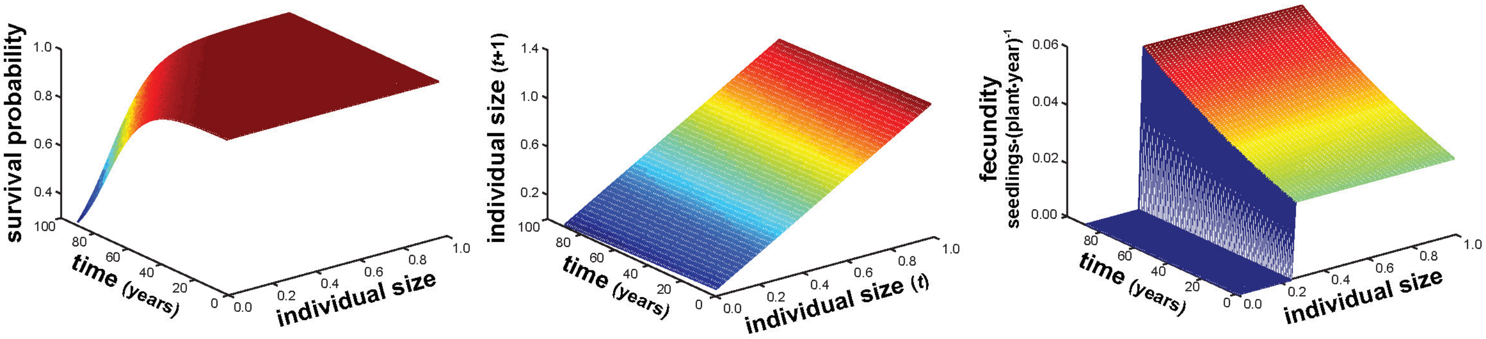

Appendix 3

Vital rates reconstructed for the artificial species

Artificial species 2

Known vital rates

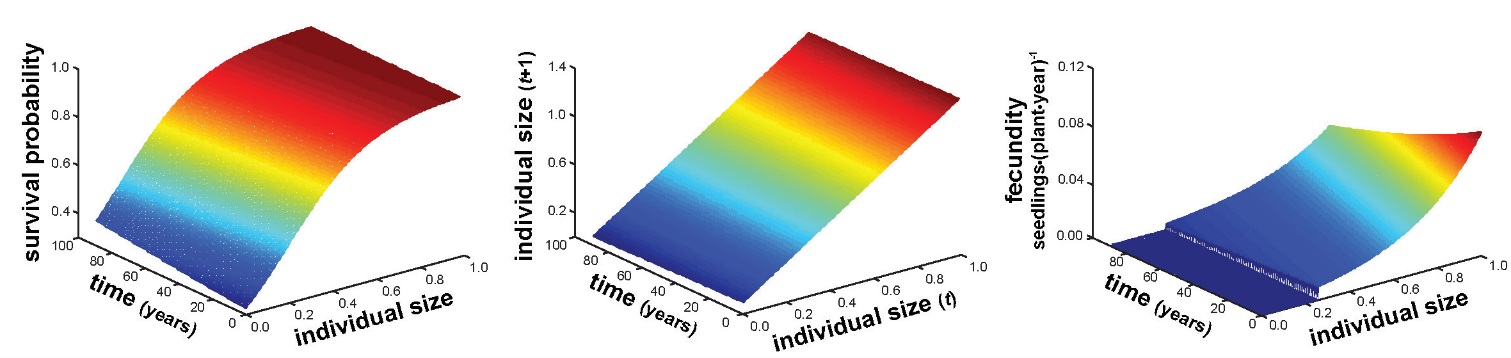

$w = 0$

$r_m =$ **0.51**

Solution 2.1

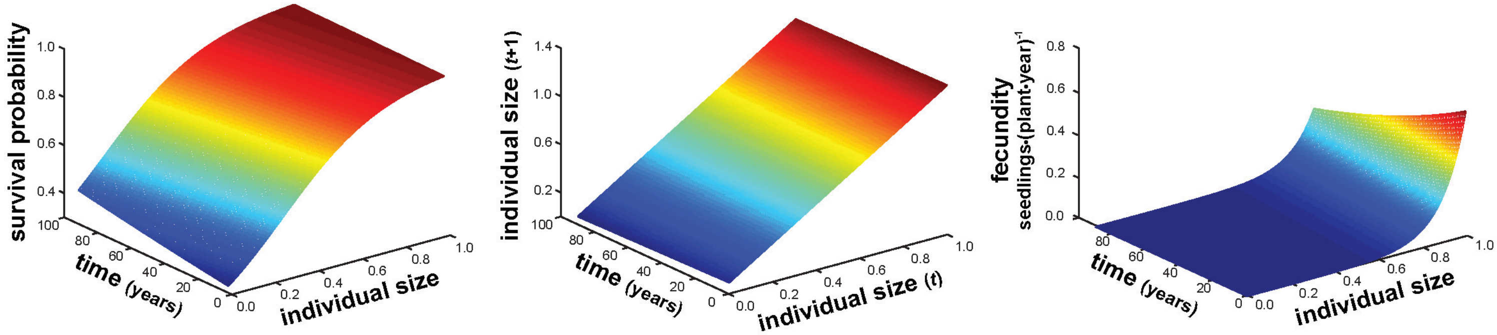

$r_m = 0.38$

Solution 2.2

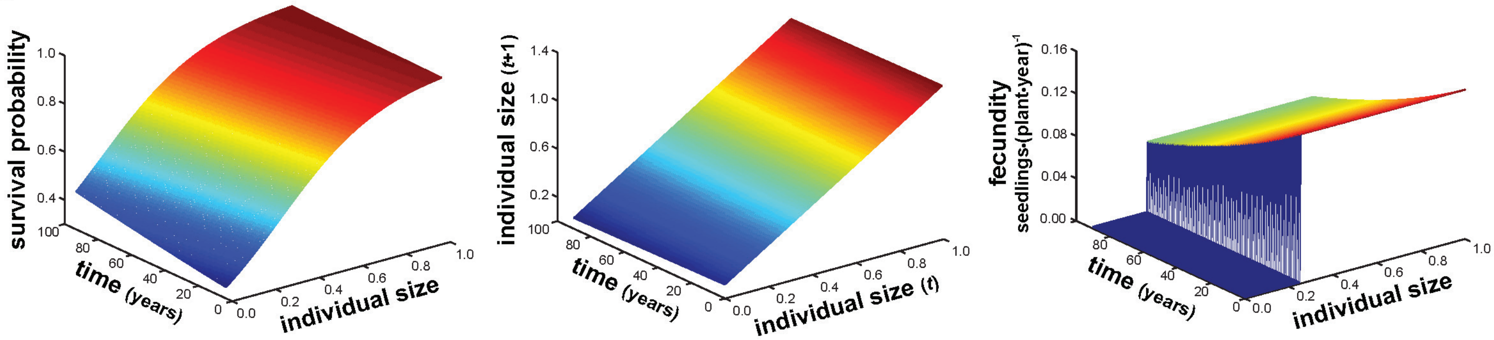

$w = 1$

$r_m = 0.84$

Solution 2.3

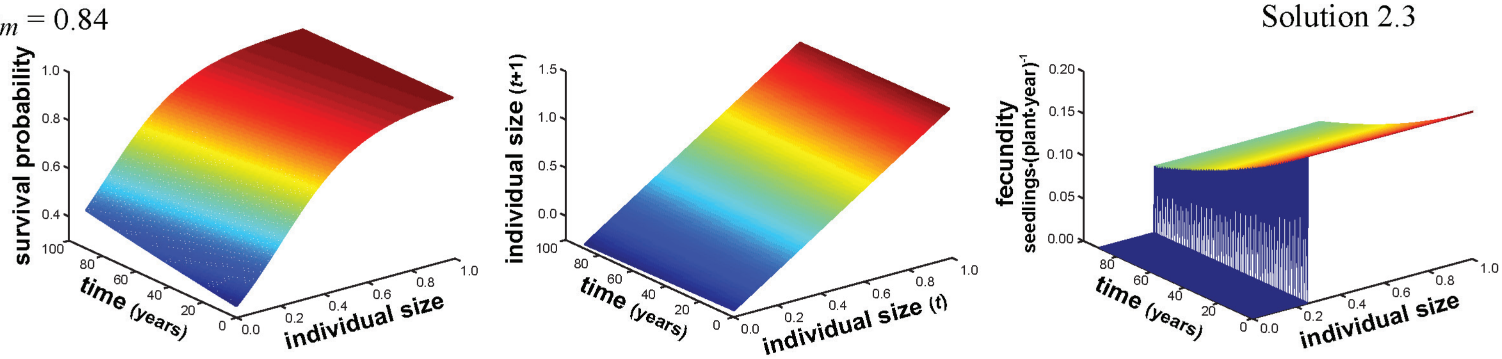

Appendix 3

Vital rates reconstructed for the artificial species

Artificial species 2 (*cont.*)

w = 1

r_m = 0.82

Solution 2.4

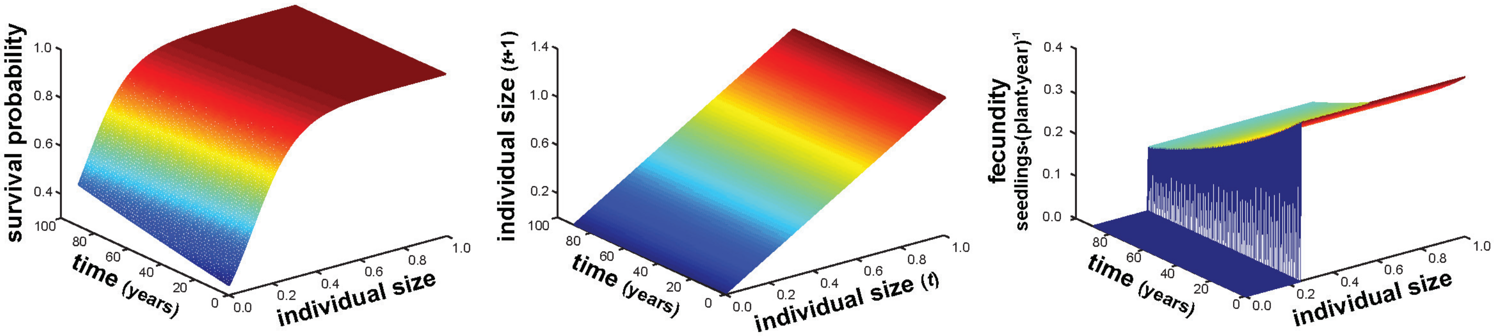

w = 10

r_m = 0.84

Solution 2.5

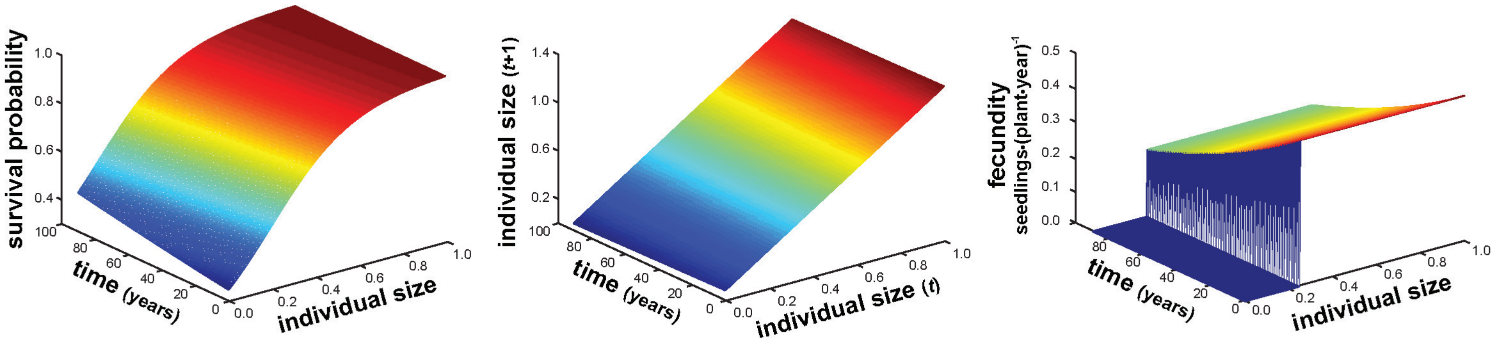

r_m = 0.82

Solution 2.6

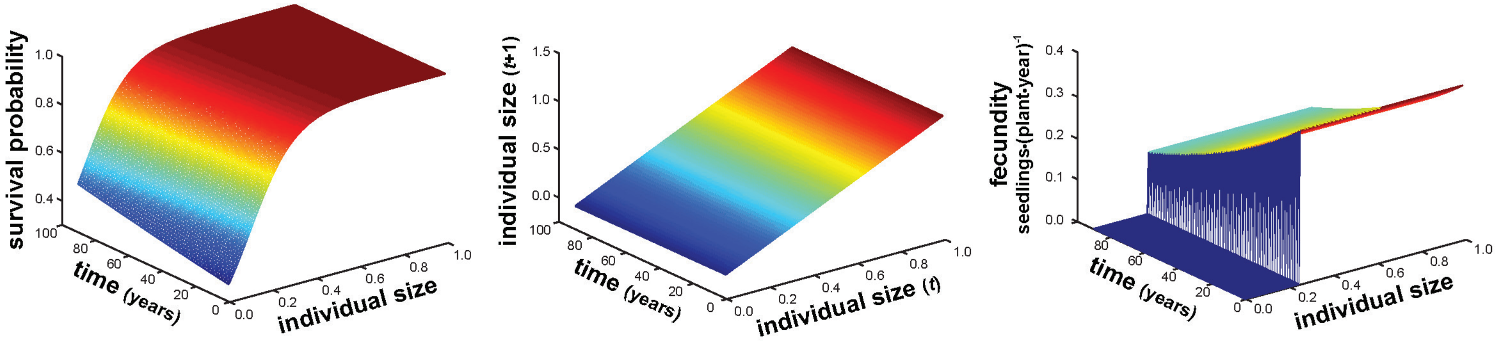

w = 100

r_m = 0.84

Solution 2.7

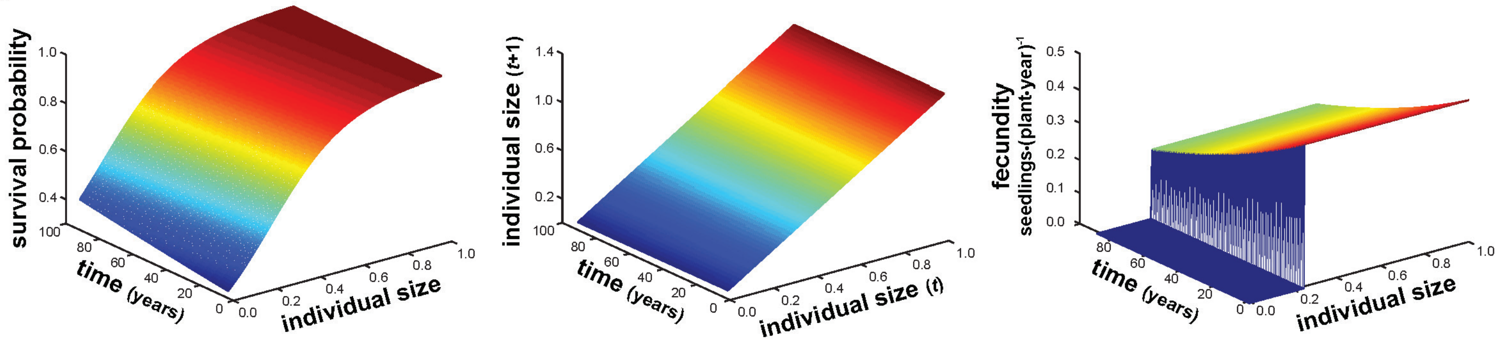

Appendix 3

Vital rates reconstructed for the artificial species

Artificial species 2 (*cont.*)

w = 1000

r_m = **0.95** Solution 2.8

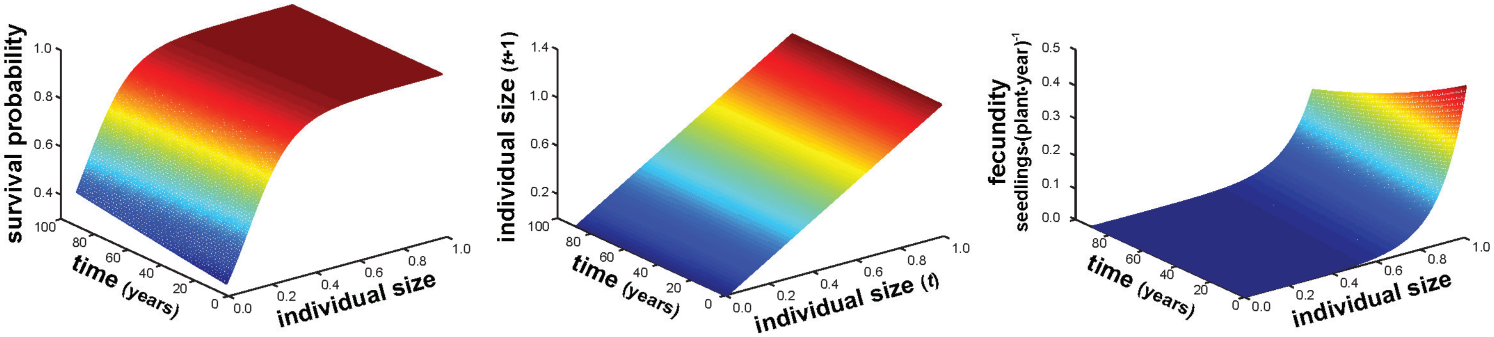

r_m = 0.84 Solution 2.9

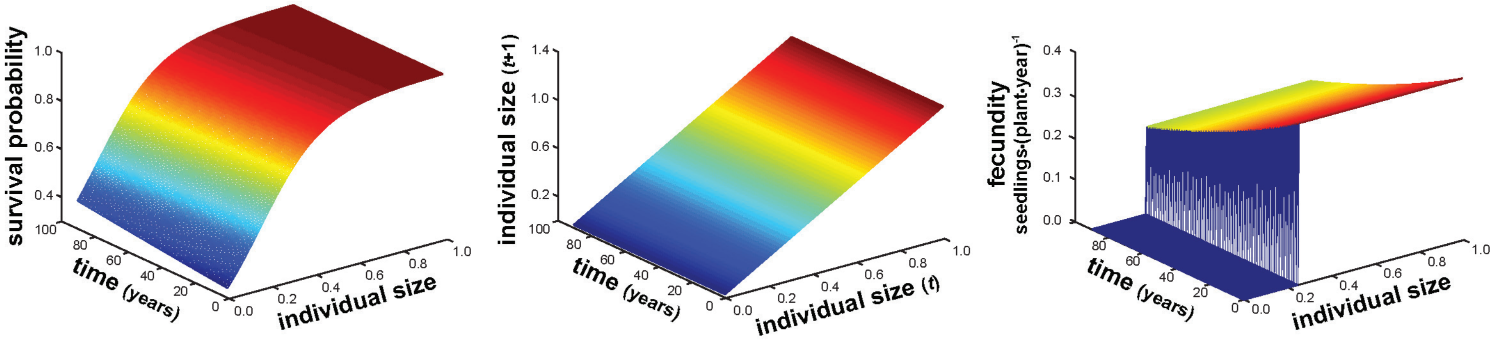

r_m = 0.49 Solution 2.10

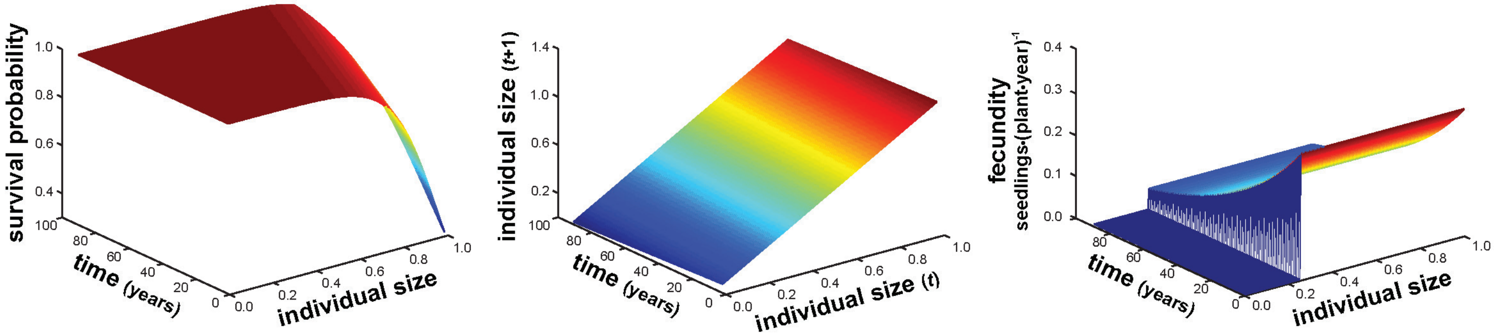

Appendix 3

Vital rates reconstructed for the artificial species

Artificial species 3

Known vital rates

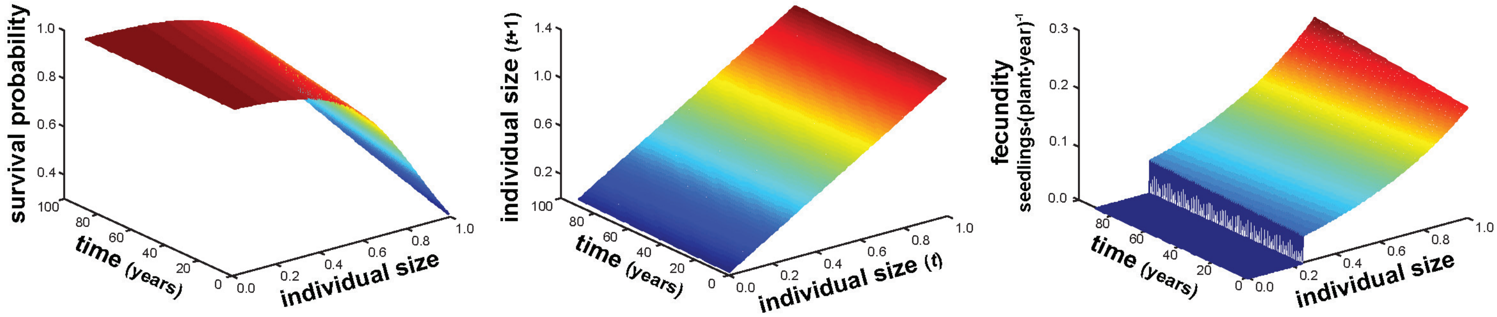

w = 0

$r_m = 0.67$

Solution 3.1

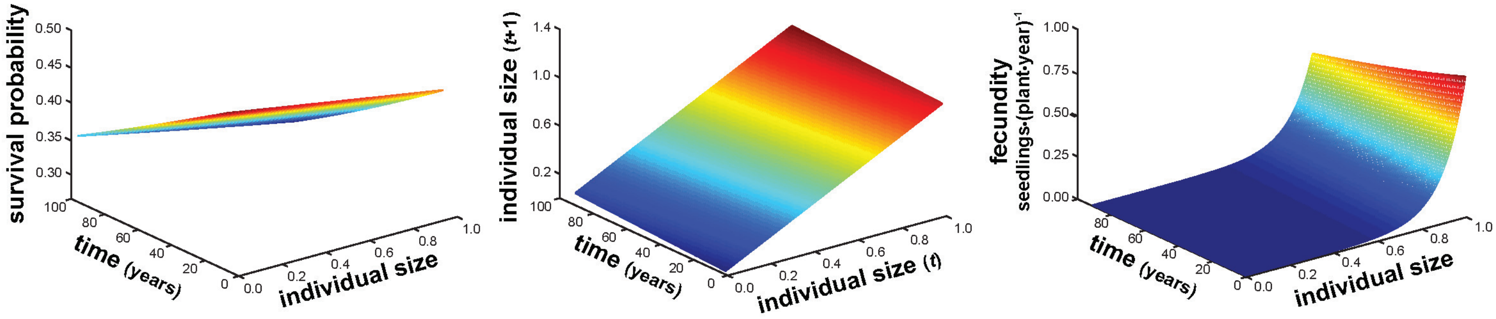

w = 1

$r_m = 0.84$

Solution 3.2

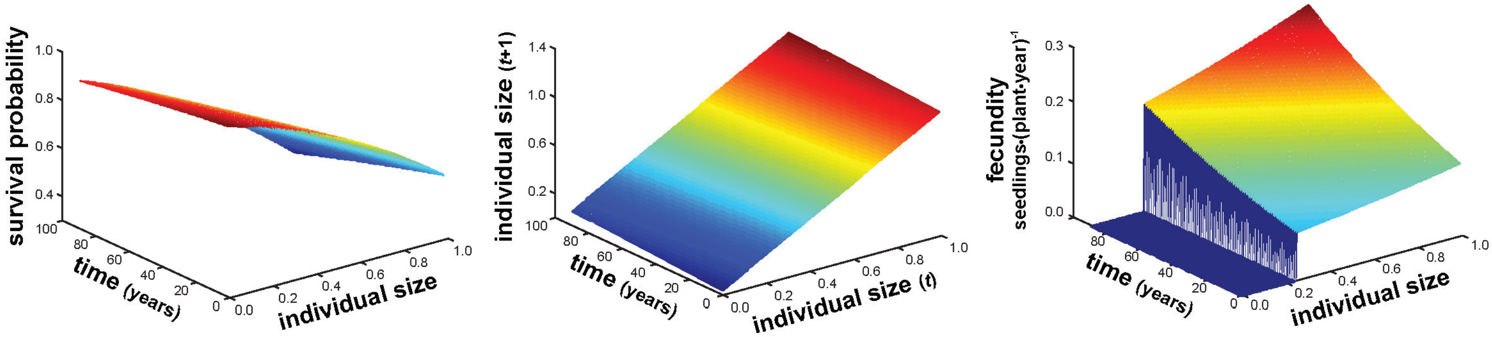

w = 10

$r_m =$ **0.97**

Solution 3.3

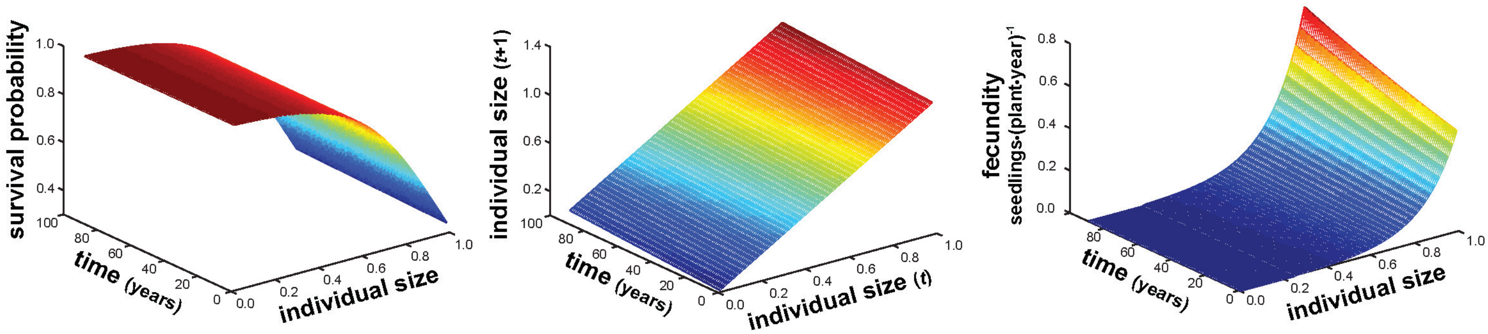

Appendix 3

Vital rates reconstructed for the artificial species

Artificial species 3 (*cont.*)

w = 10

r_m = 0.69

Solution 3.4

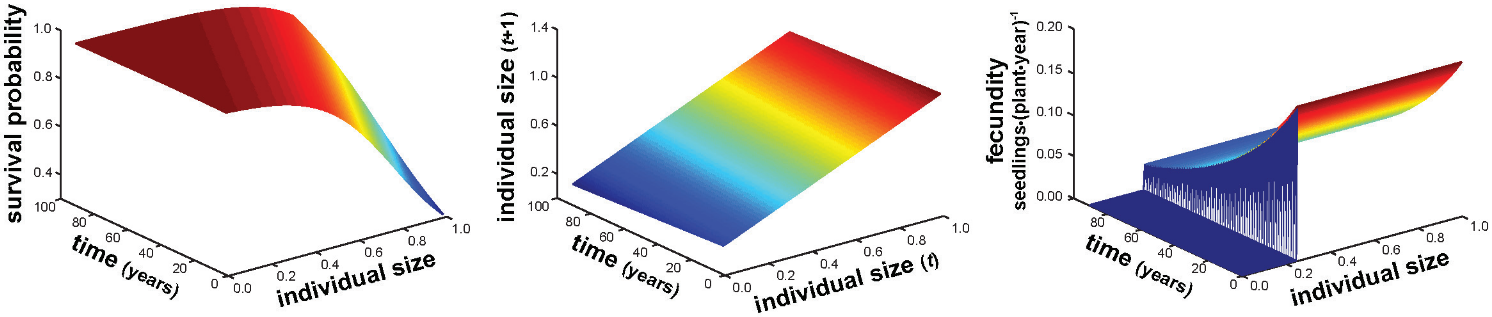

w = 100

r_m = **0.96**

Solution 3.5

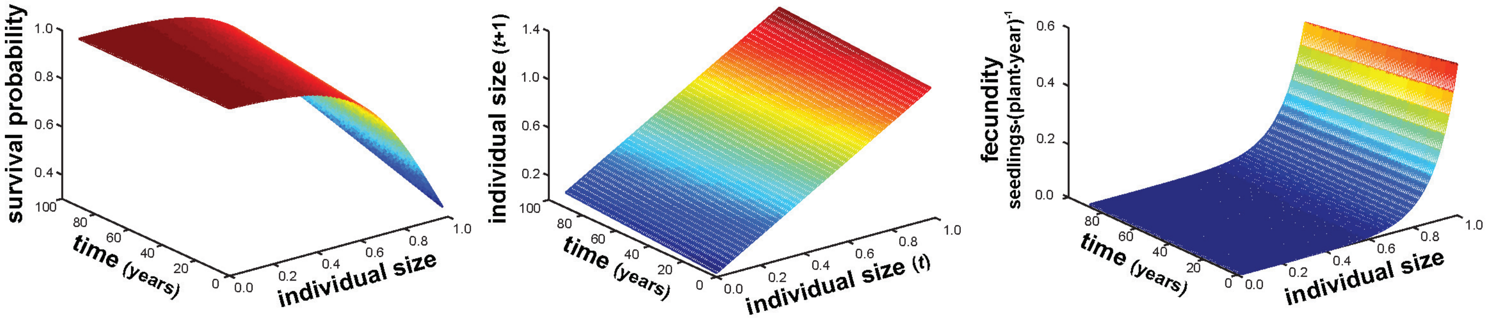

r_m = 0.85

Solution 3.6

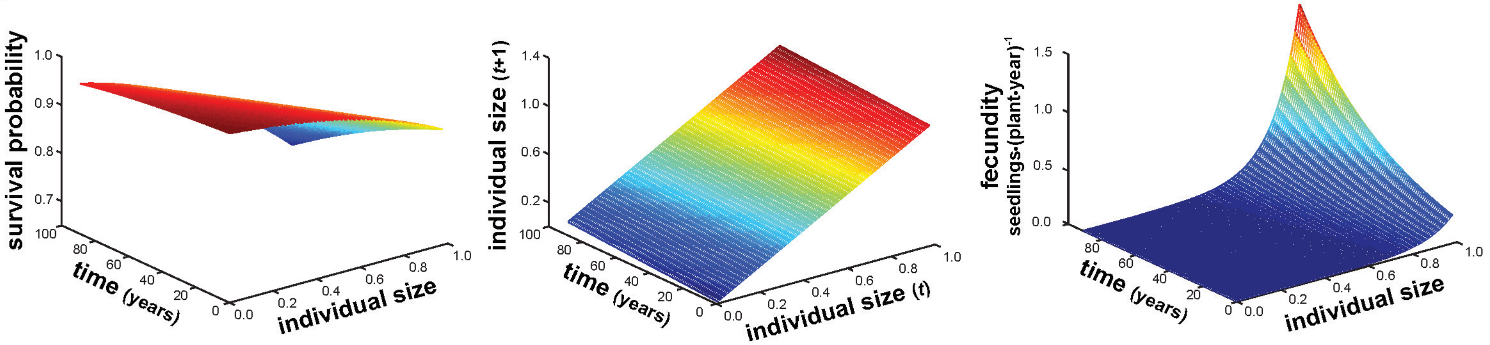

r_m = 0.69

Solution 3.7

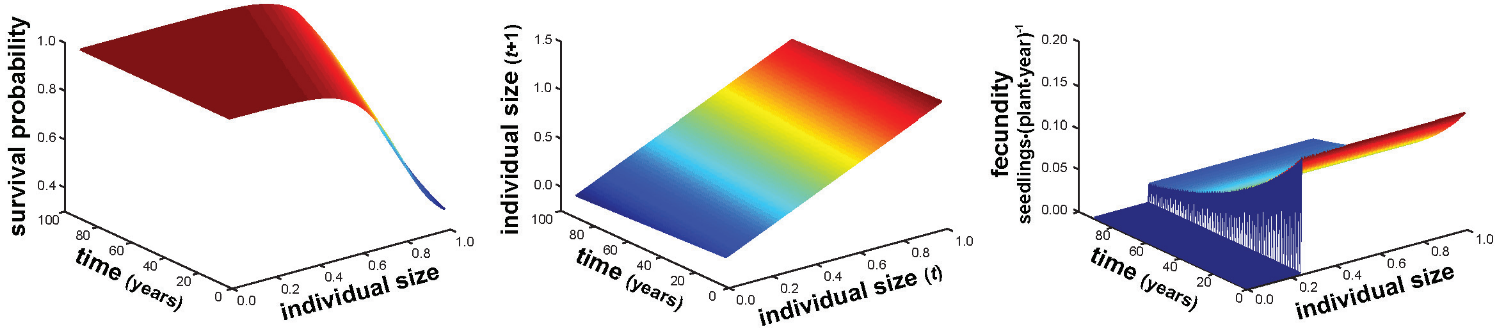

Appendix 3

Vital rates reconstructed for the artificial species

Artificial species 3 (*cont.*)

w = 100

r_m = 0.59

Solution 3.8

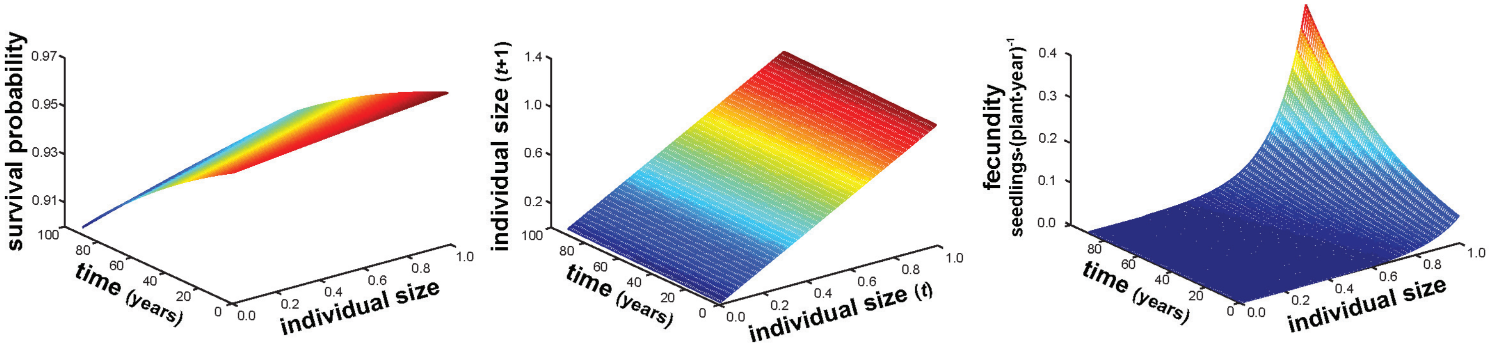

w = 1000

r_m = **0.99**

Solution 3.9

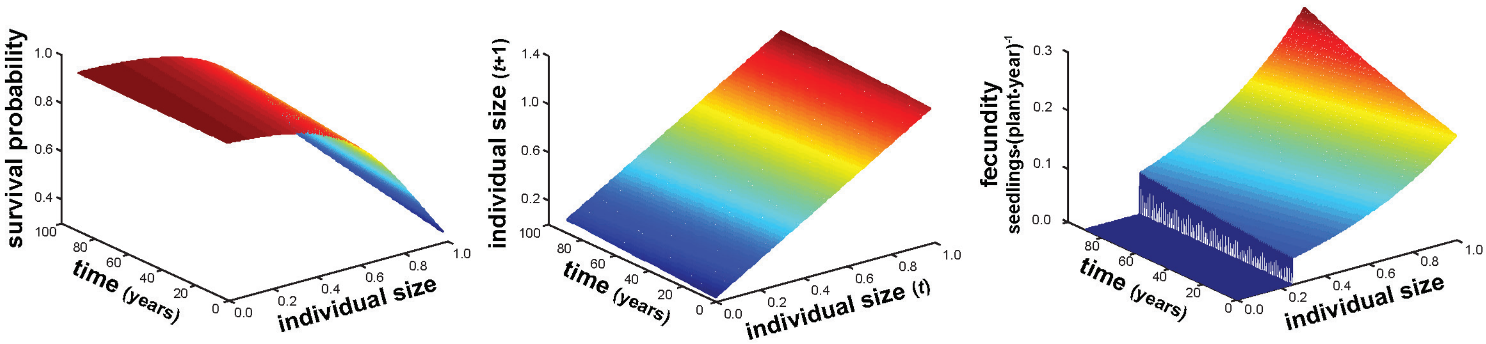

r_m = 0.94

Solution 3.10

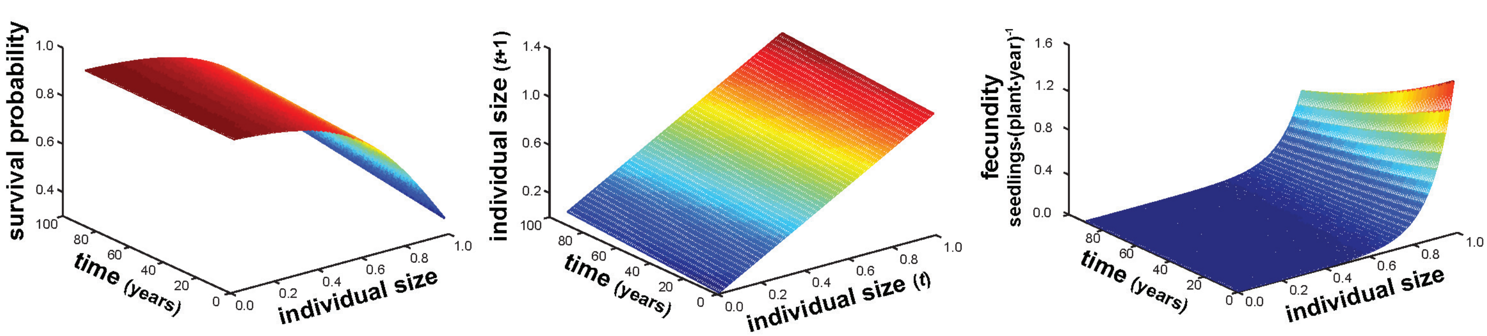

r_m = 0.68

Solution 3.11

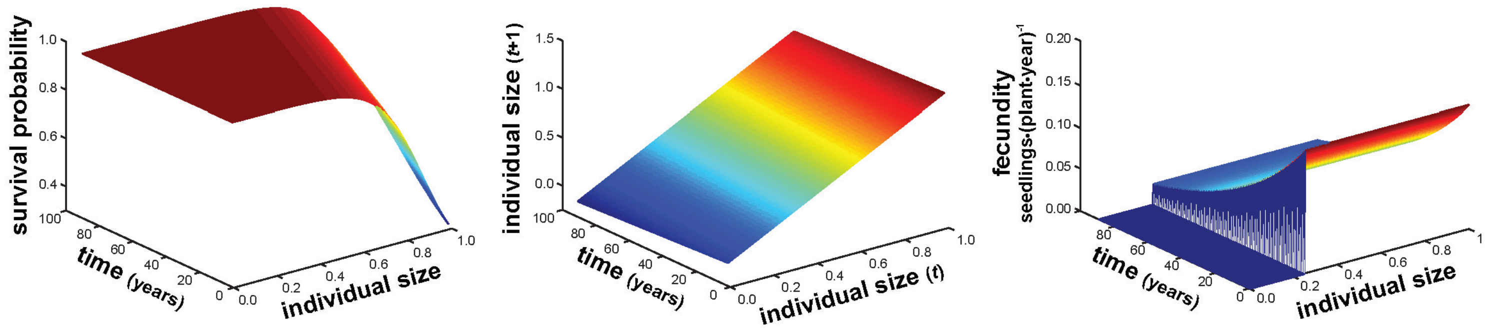

Appendix 3

Vital rates reconstructed for the artificial species

Artificial species 3 (*cont.*)

w = 1000

r_m = 0.59

Solution 3.12

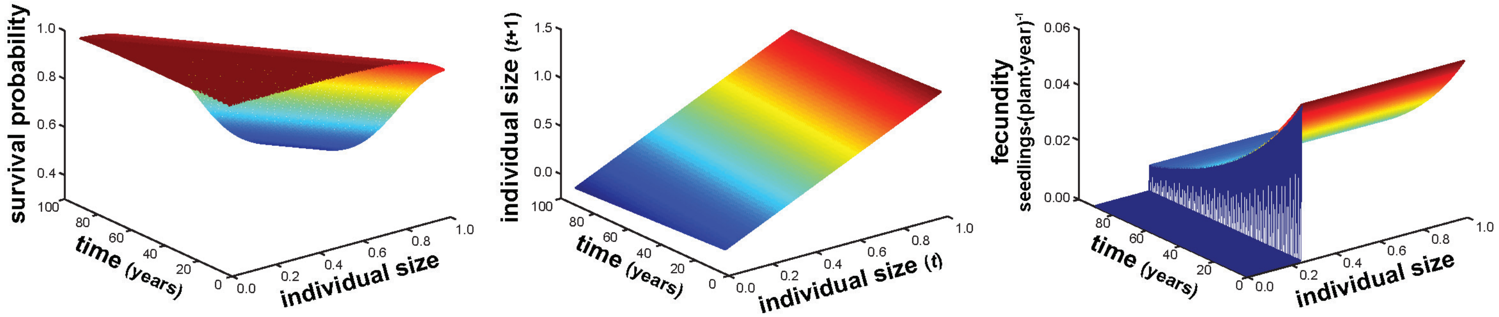

r_m = 0.44

Solution 3.13

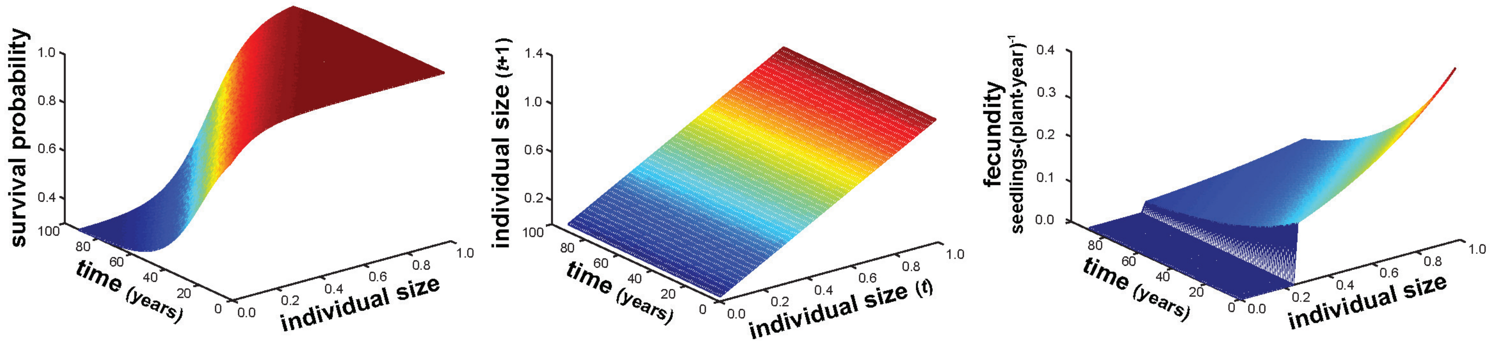

Appendix 3

Vital rates reconstructed for the artificial species

Artificial species 4

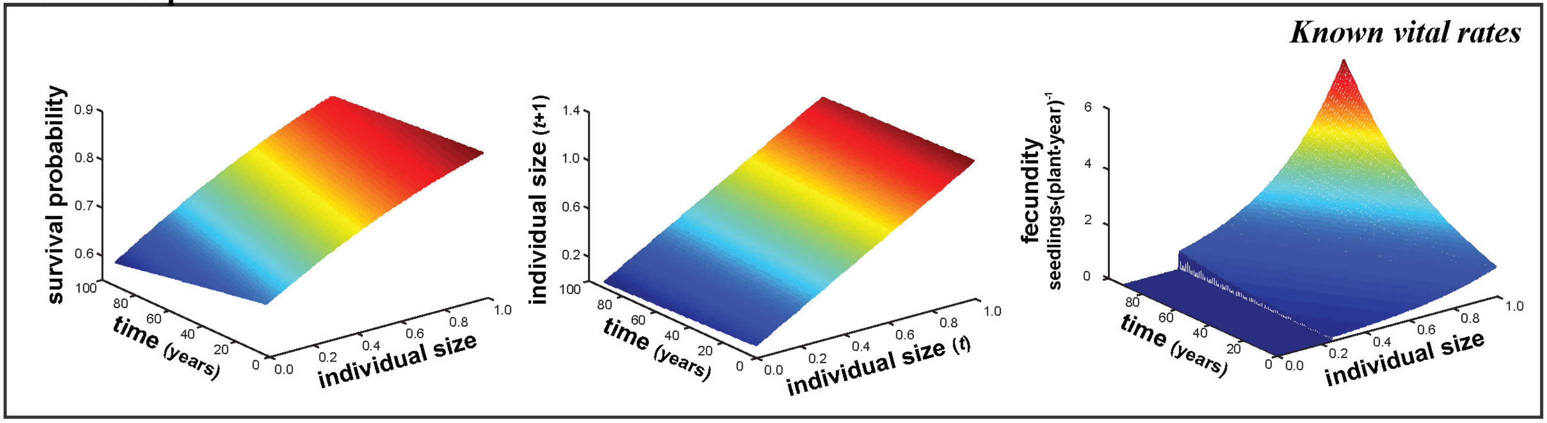

$w = 0$

$r_m = 0.60$

Solution 4.1

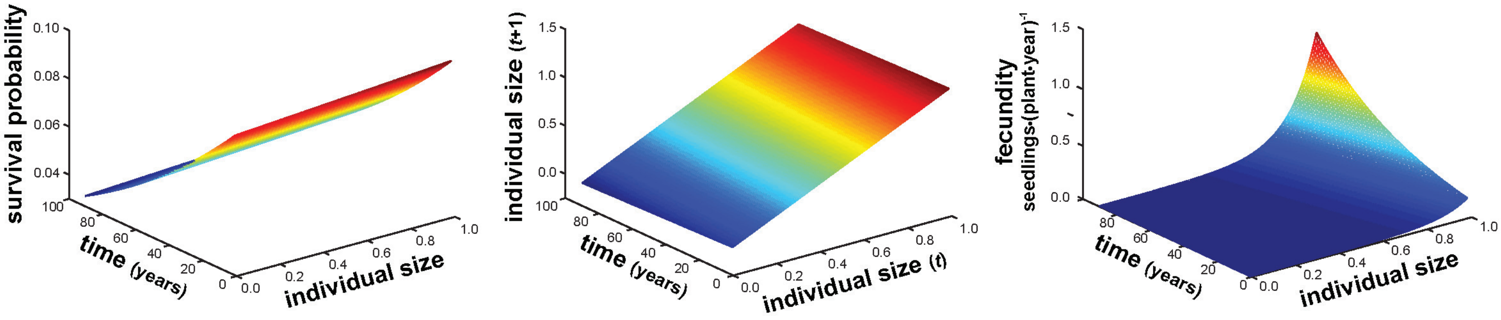

$r_m =$ **0.51**

Solution 4.2

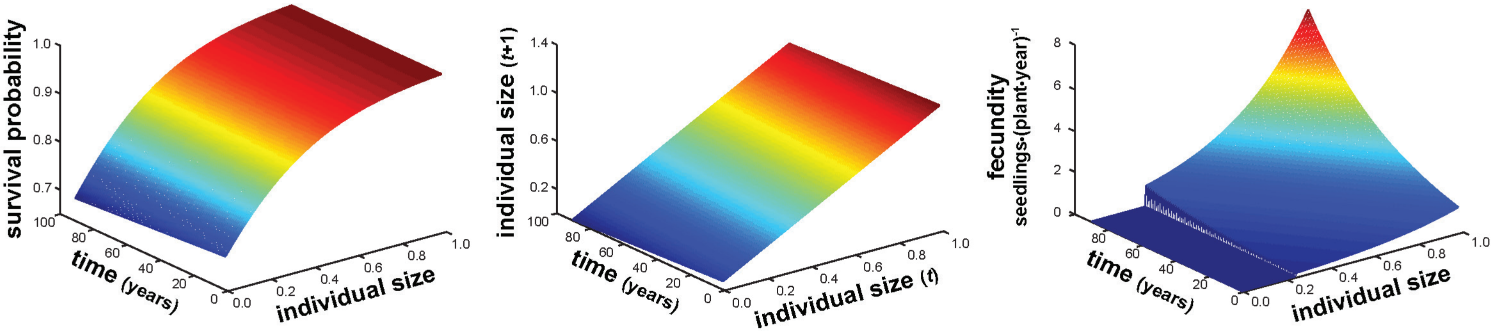

$r_m = 0.34$

Solution 4.3

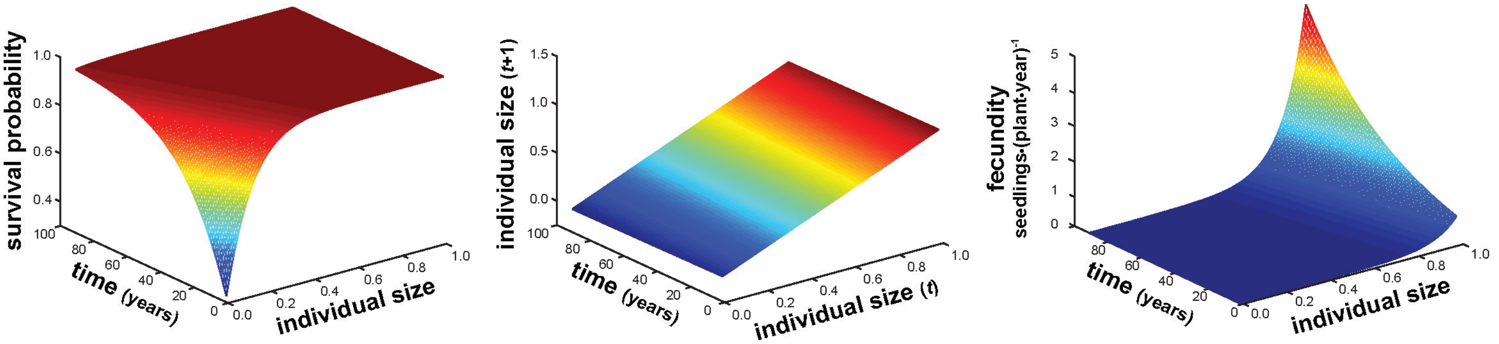

Appendix 3

Vital rates reconstructed for the artificial species

Artificial species 4 (*cont.*)

$w = 0$

$r_m = 0.34$

Solution 4.4

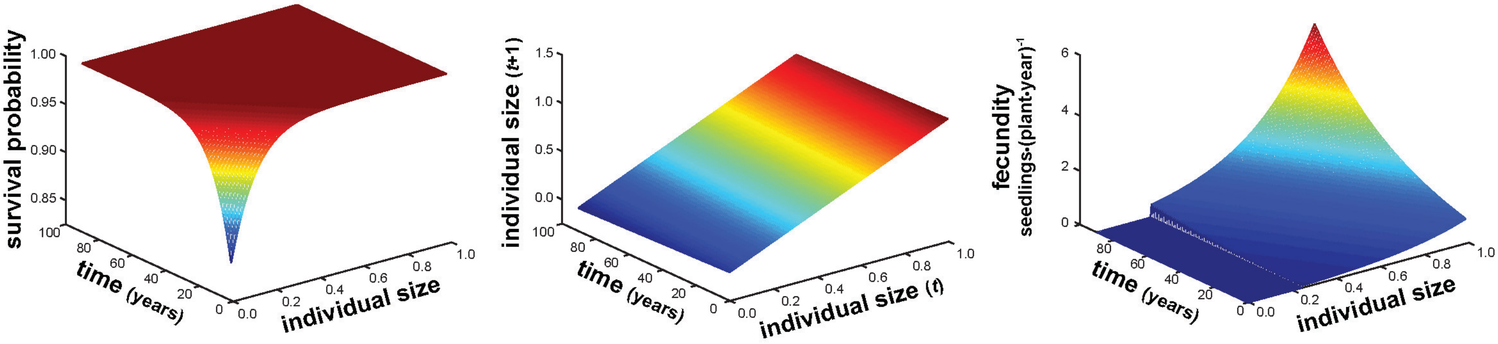

$r_m = 0.13$

Solution 4.5

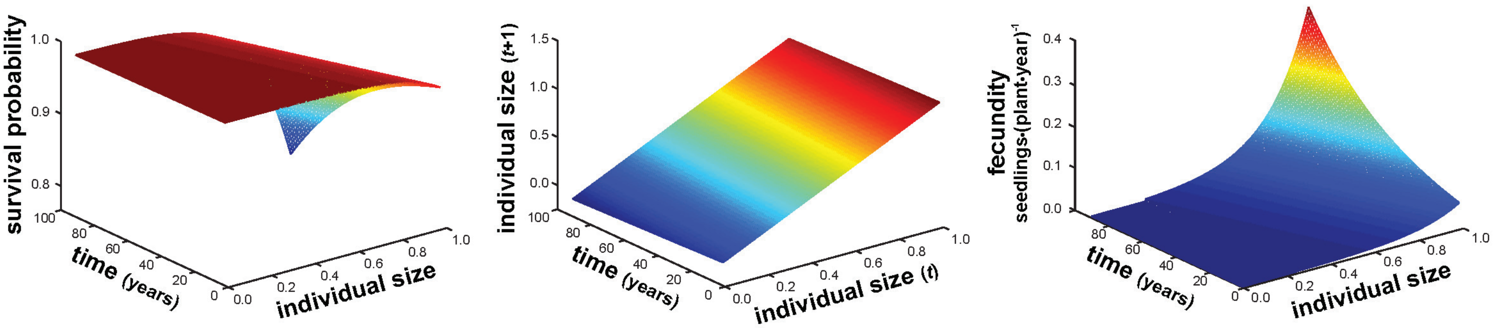

$w = 1$

$r_m =$ **0.98**

Solution 4.6

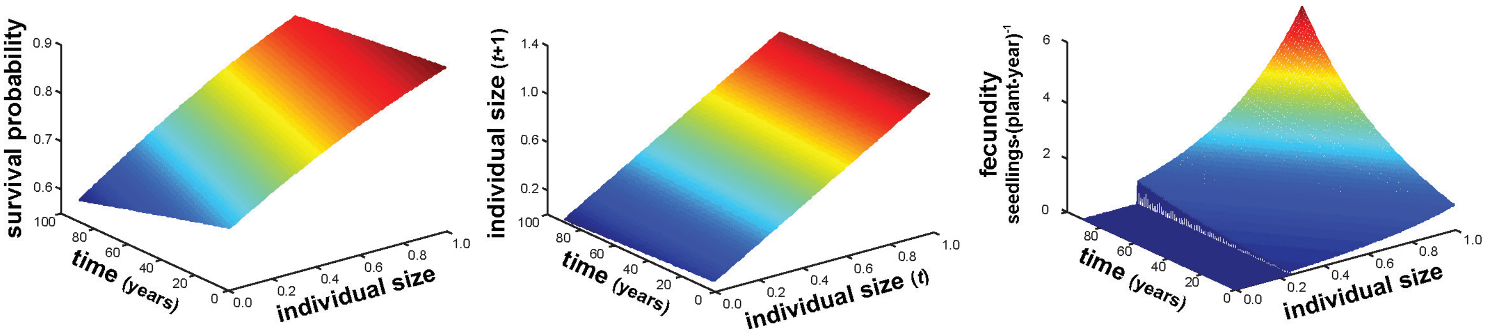

$r_m = 0.32$

Solution 4.7

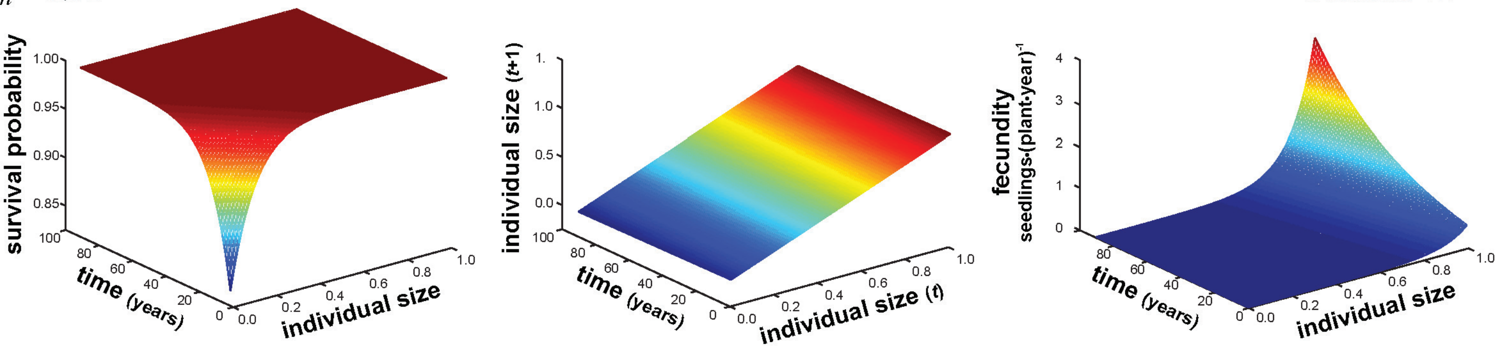

Appendix 3

Vital rates reconstructed for the artificial species

Artificial species 4 (*cont.*)

$w = 10$

r_m = **0.98** Solution 4.8

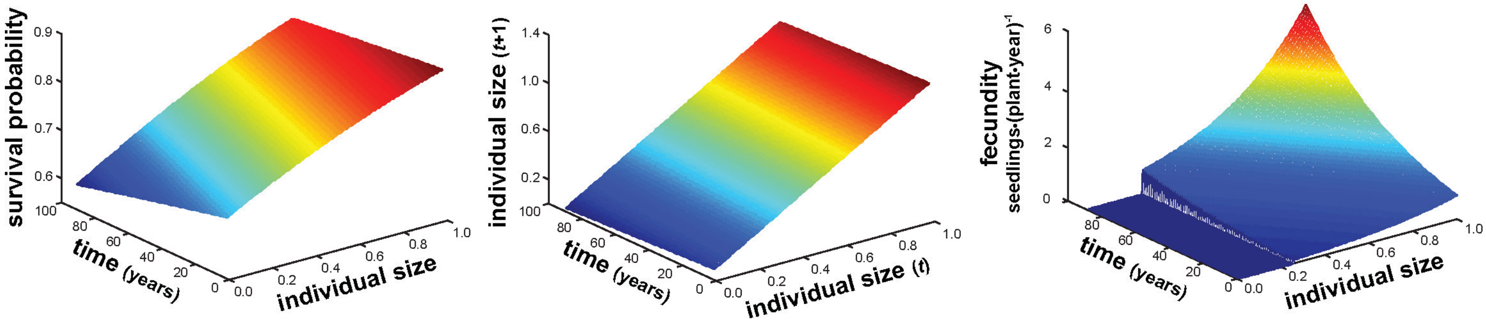

$r_m = 0.82$ Solution 4.9

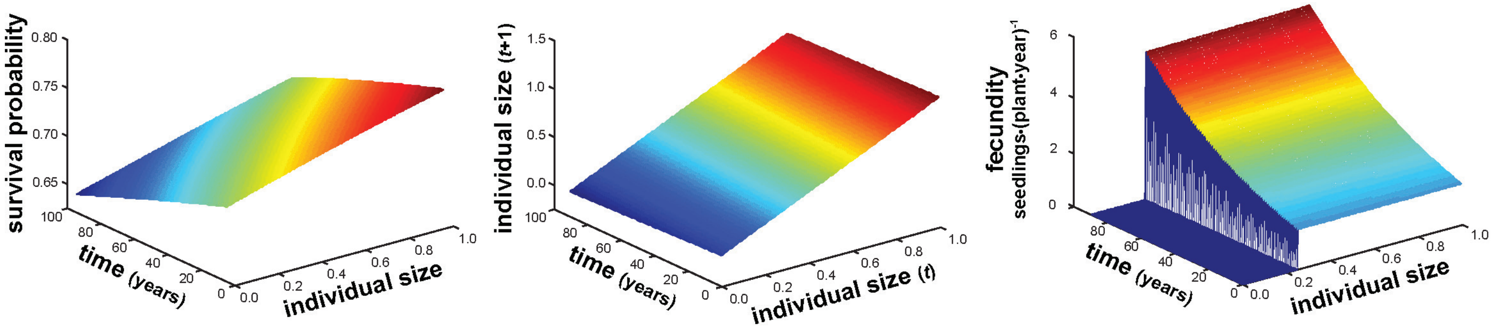

$w = 100$

r_m = **0.93** Solution 4.10

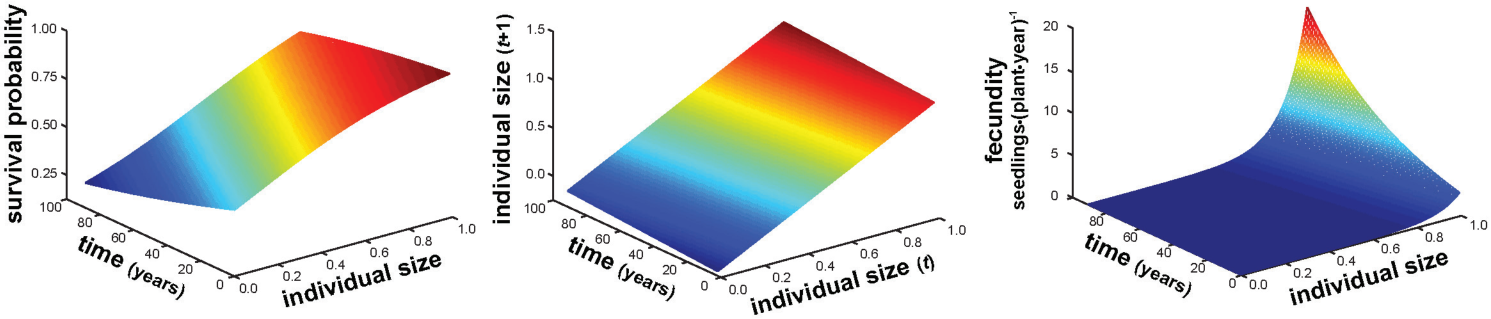

r_m = **0.93** Solution 4.11

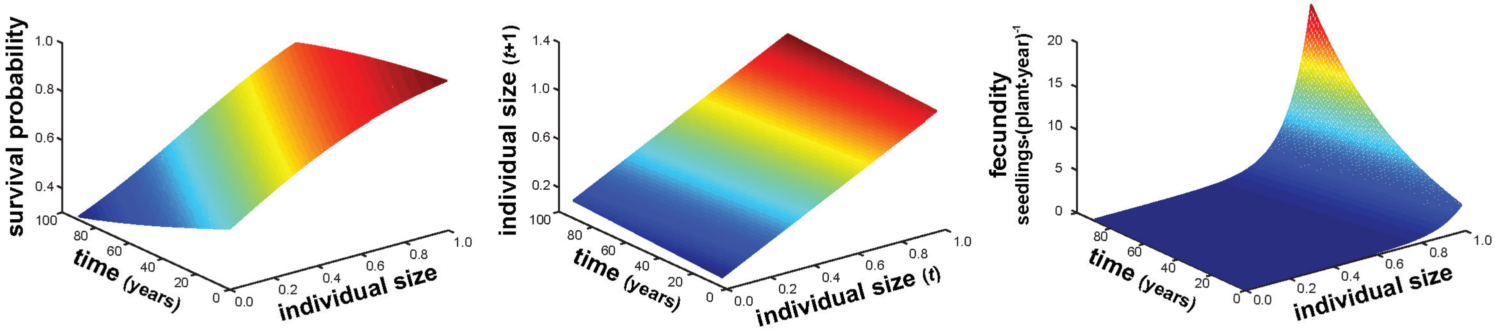

Appendix 3

Vital rates reconstructed for the artificial species

Artificial species 4 (*cont.*)

w = 100

$r_m = 0.91$

Solution 4.12

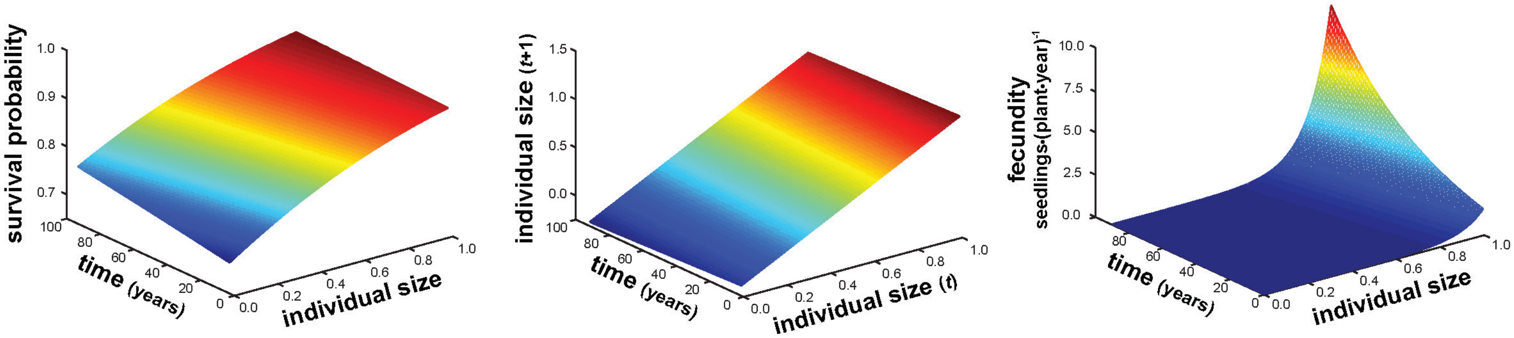

r_m = 0.88

Solution 4.13

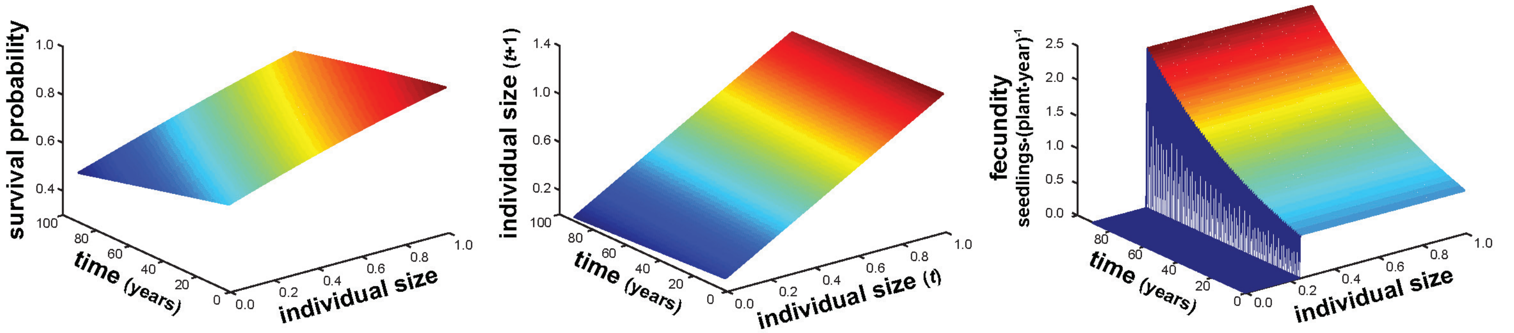

r_m = 0.67

Solution 4.14

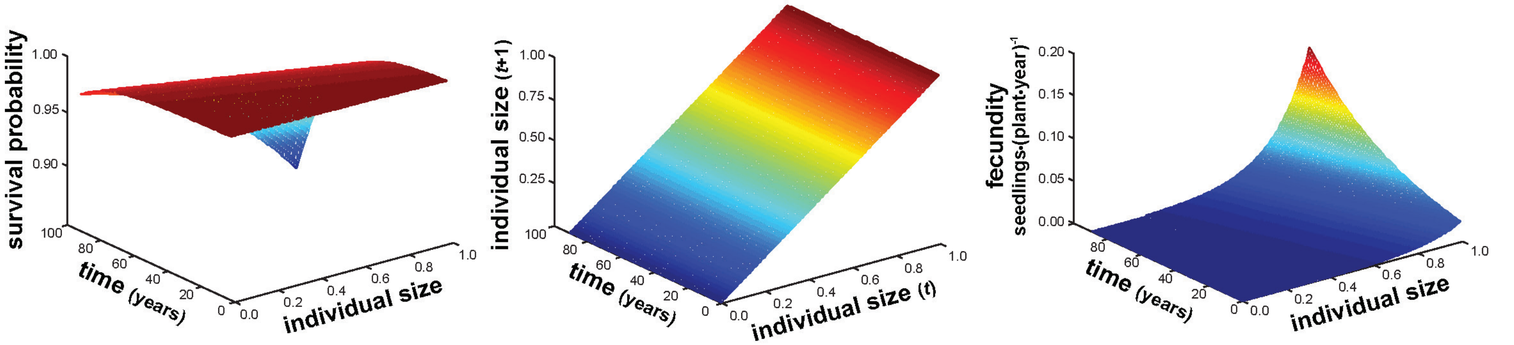

r_m = 0.56

Solution 4.15

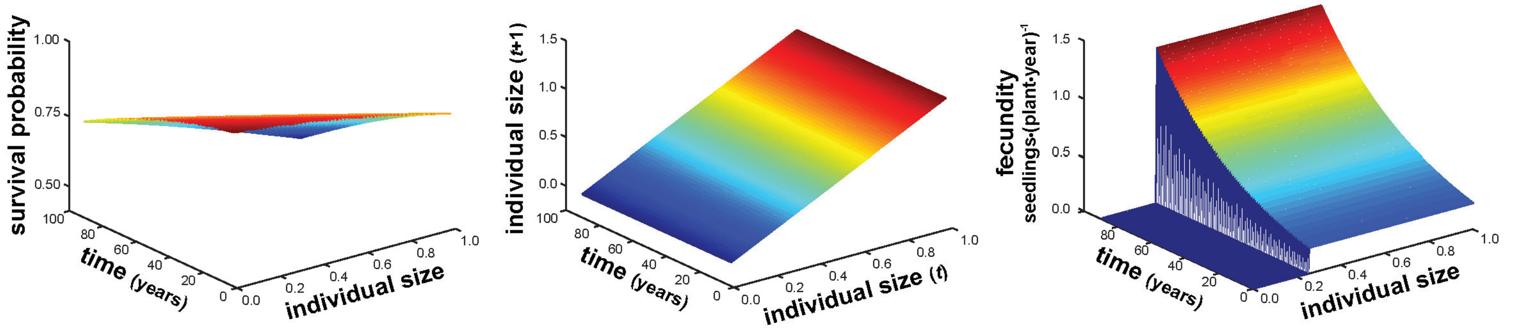

Appendix 3

Vital rates reconstructed for the artificial species

Artificial species 4 (*cont.*)

w = 100

r_m = 0.56

Solution 4.16

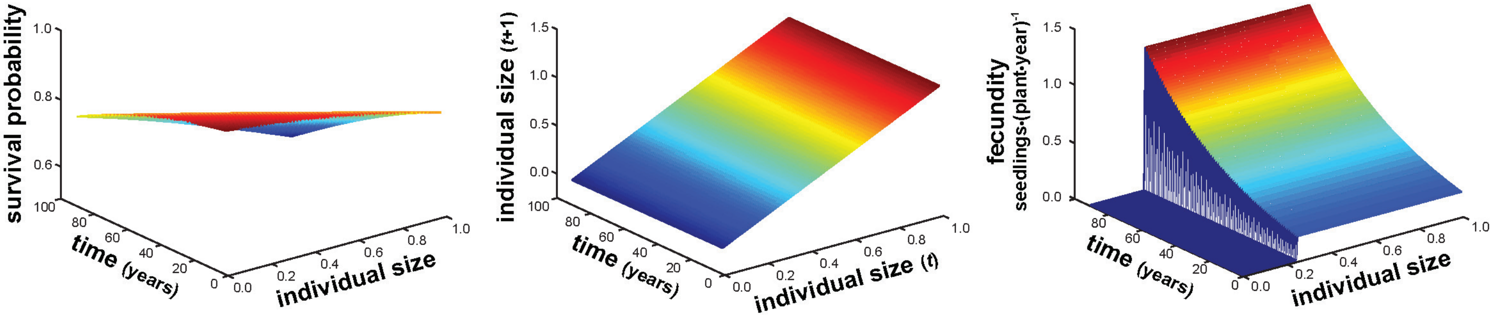

w = 1000

r_m = *0.93*

Solution 4.17

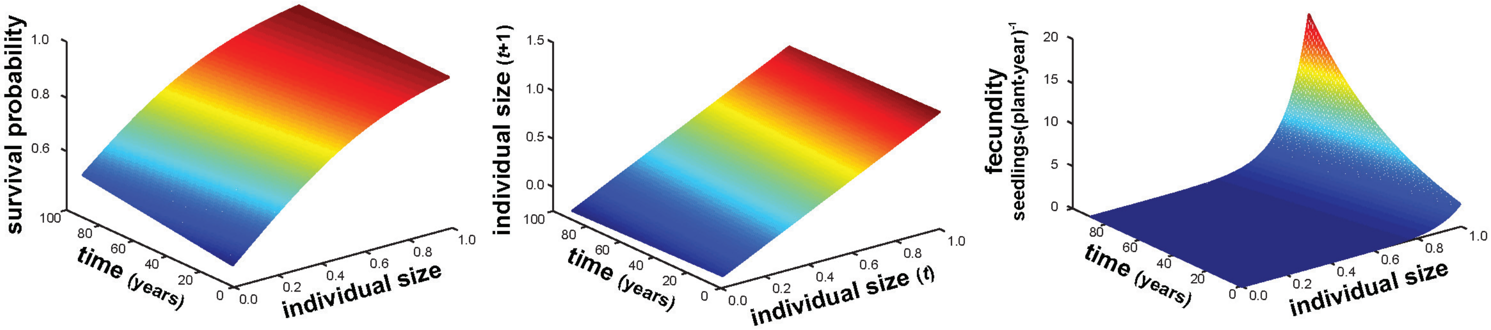

r_m = *0.75*

Solution 4.18

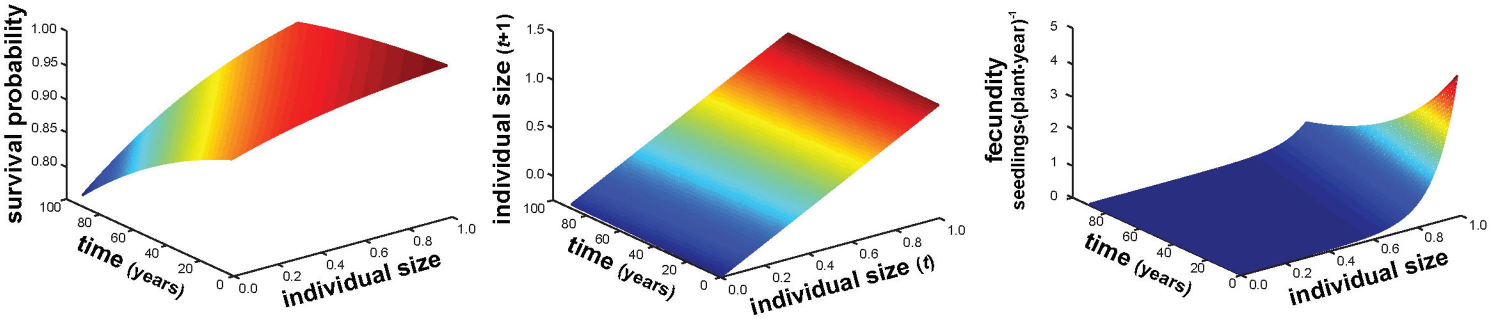

r_m = 0.60

Solution 4.19

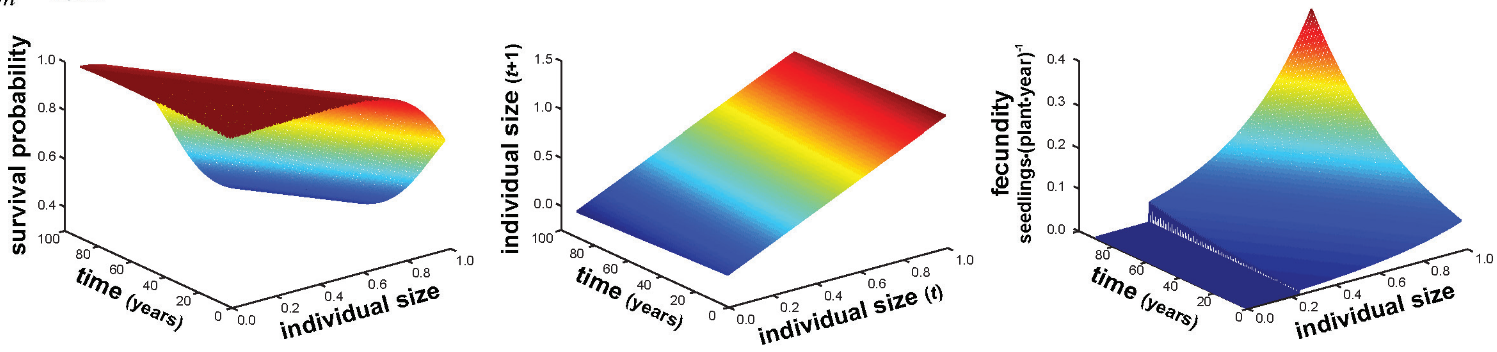

Appendix 3

Vital rates reconstructed for the artificial species

Artificial species 5

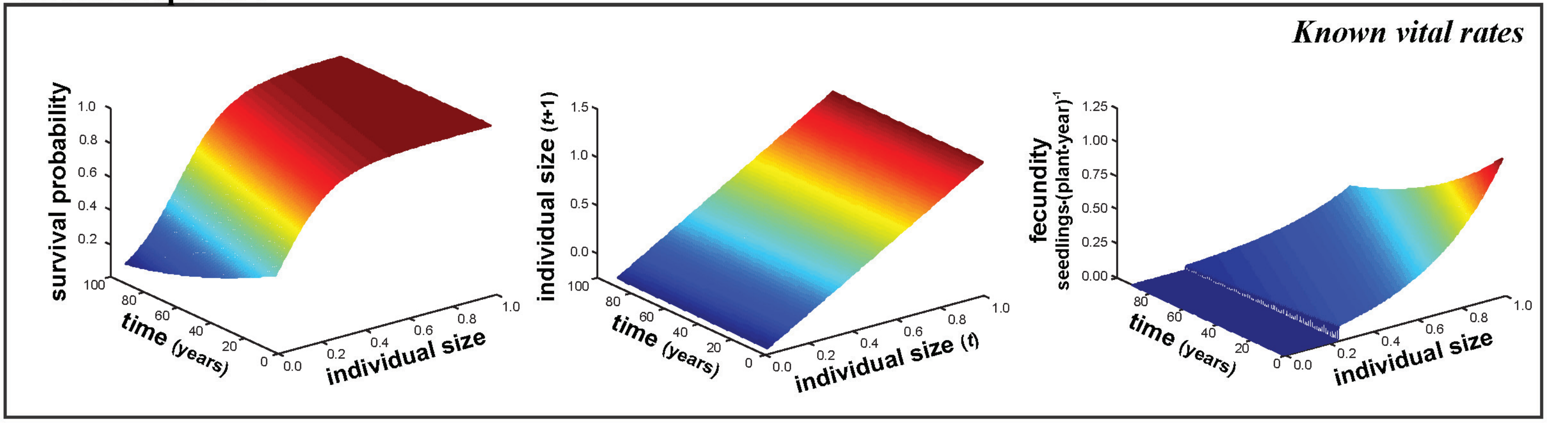

w = 0

r_m = **0.99**

Solution 5.1

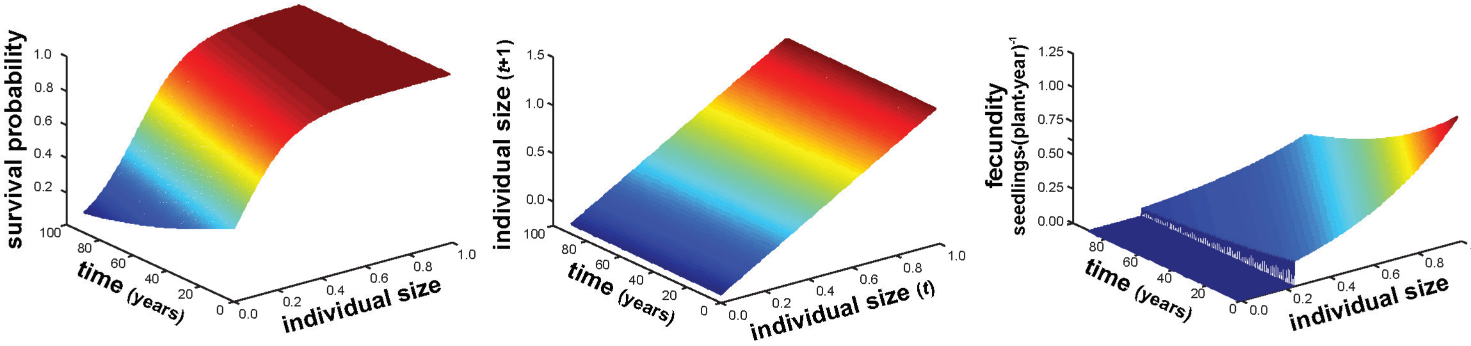

w = 1

r_m = **0.99**

Solution 5.2

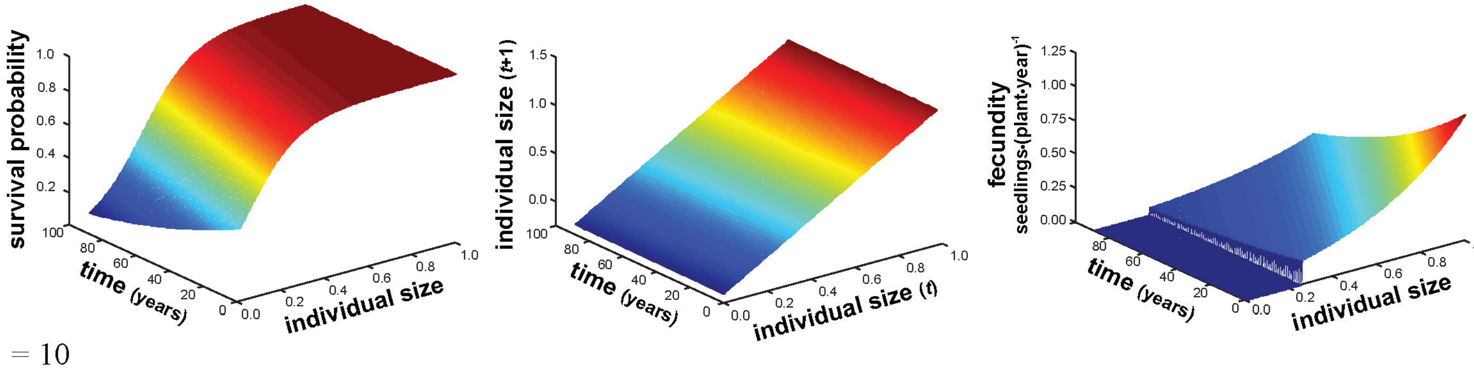

w = 10

r_m = **1.00**

Solution 5.3

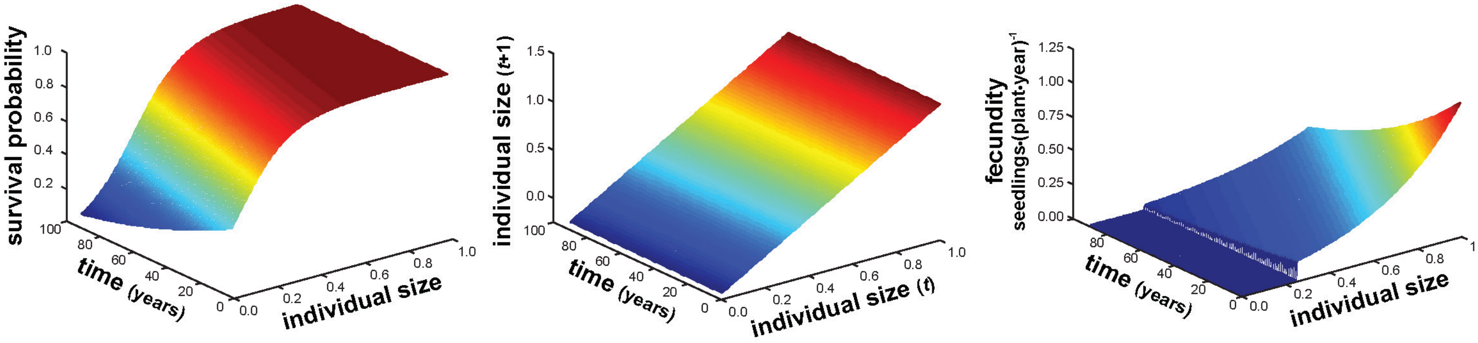

Appendix 3

Vital rates reconstructed for the artificial species

Artificial species 5 (*cont.*)

w = 100

r_m = **0.99** Solution 5.4

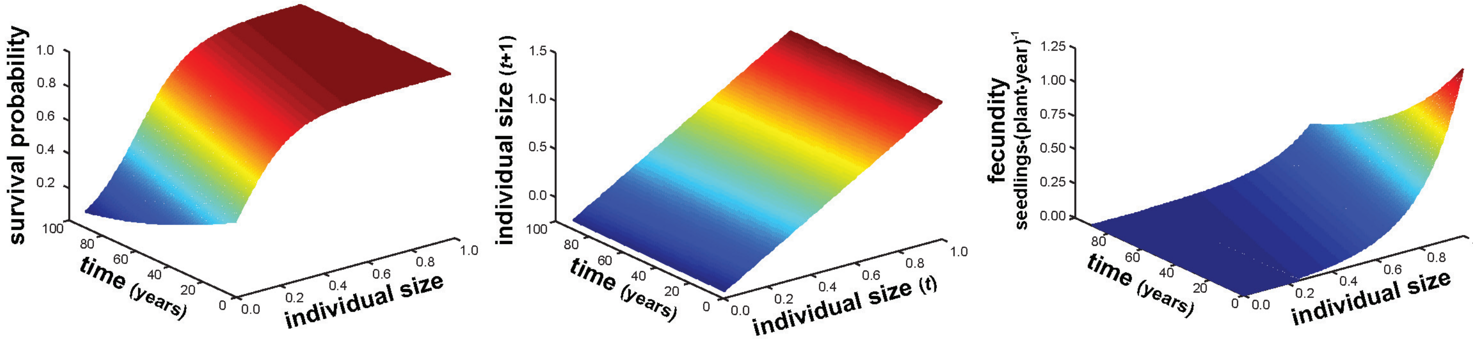

w = 1000

r_m = **0.99** Solution 5.5

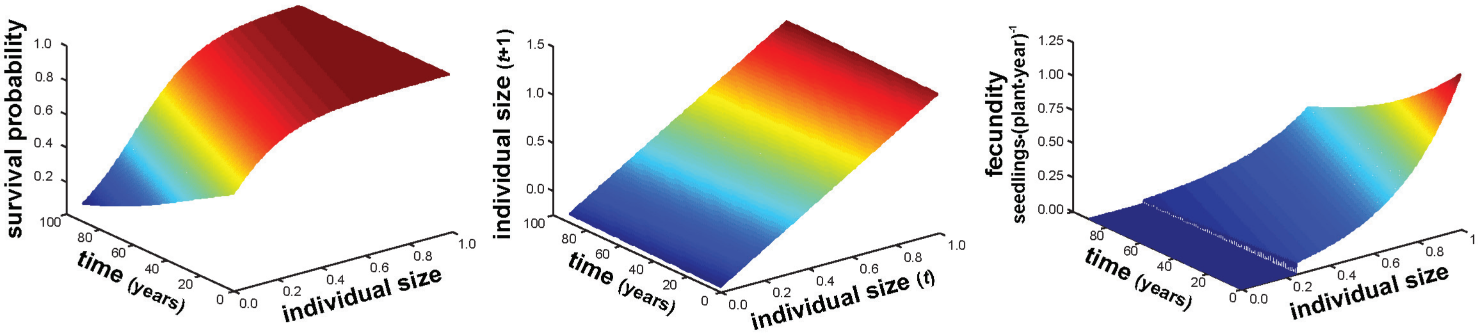

r_m = **0.95** Solution 5.6

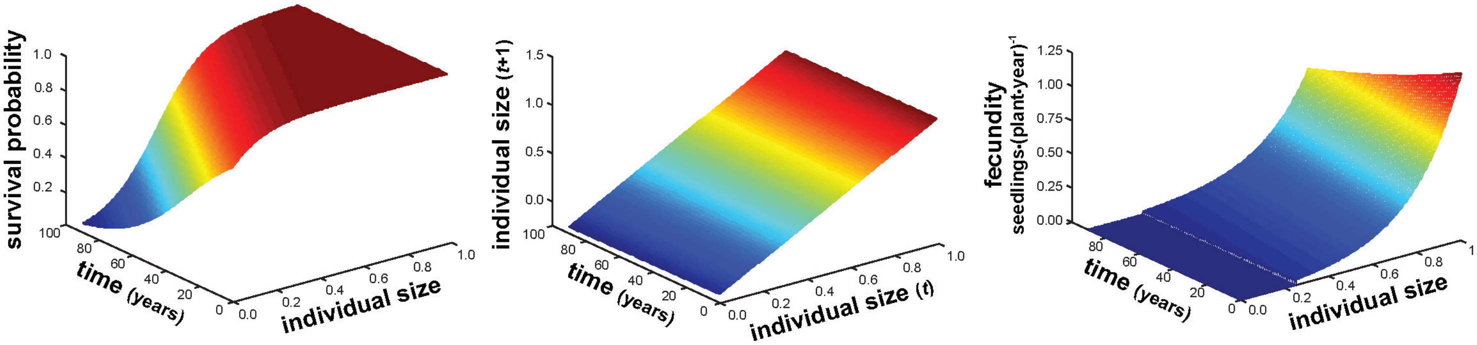

Appendix 3

Vital rates reconstructed for the artificial species

Artificial species 6

Known vital rates

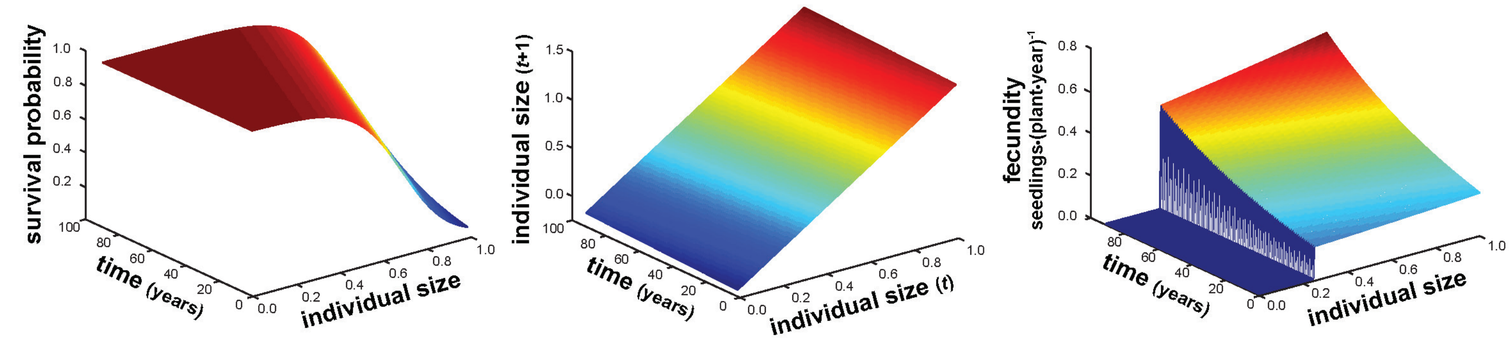

w = 0

r_m = **0.90**

Solution 6.1

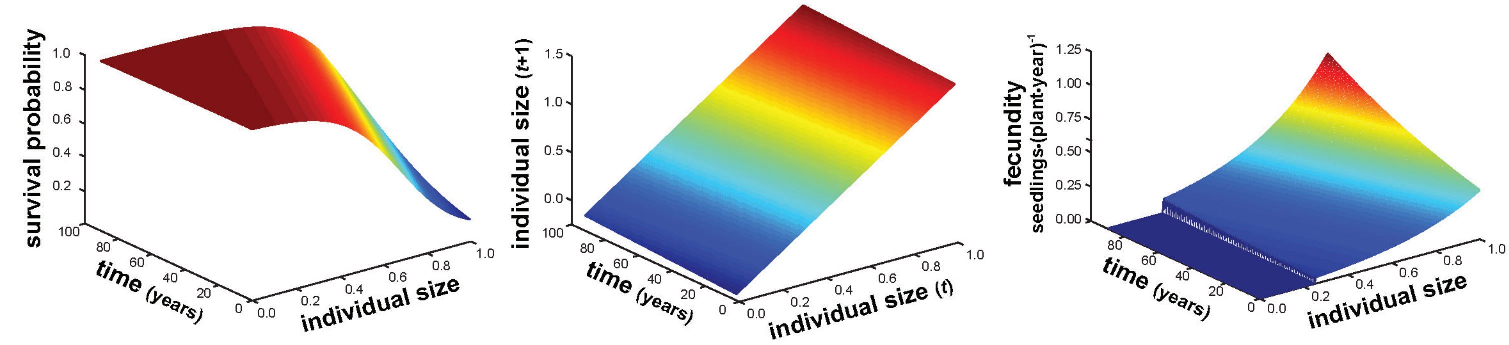

w = 1

r_m = **1.00**

Solution 6.2

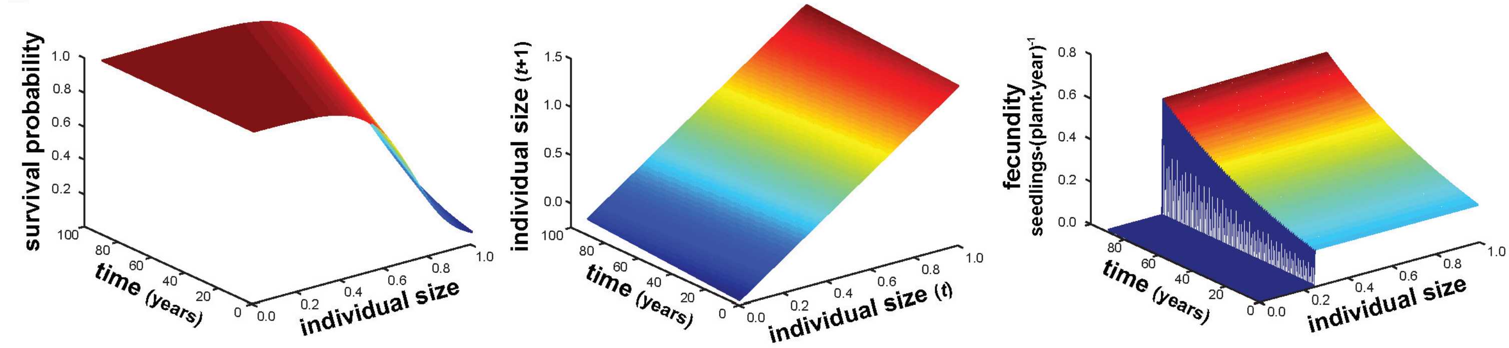

r_m = 0.54

Solution 6.3

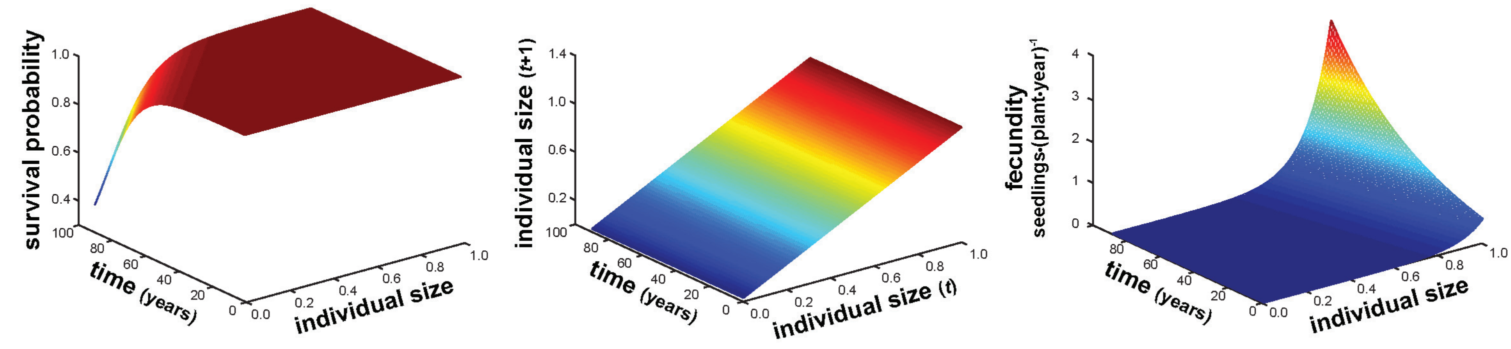

Appendix 3

Vital rates reconstructed for the artificial species

Artificial species 6 (*cont.*)

w = 10

r_m = **1.00**

Solution 6.4

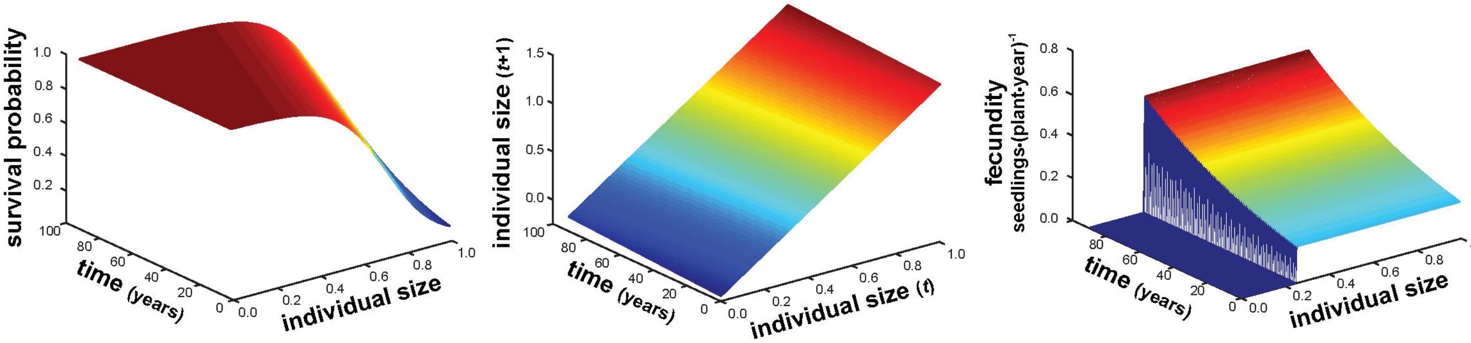

w = 100

r_m = **0.99**

Solution 6.5

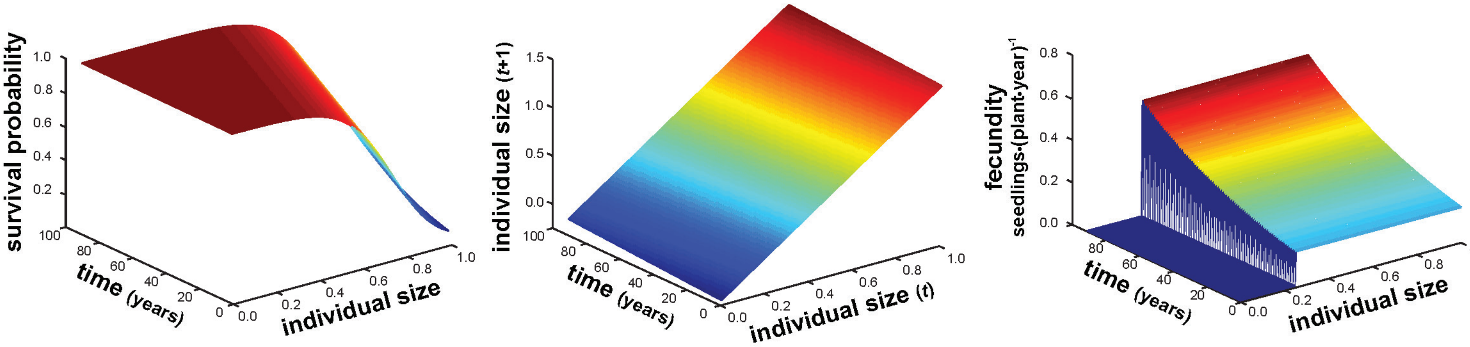

w = 1000

r_m = **0.99**

Solution 6.6

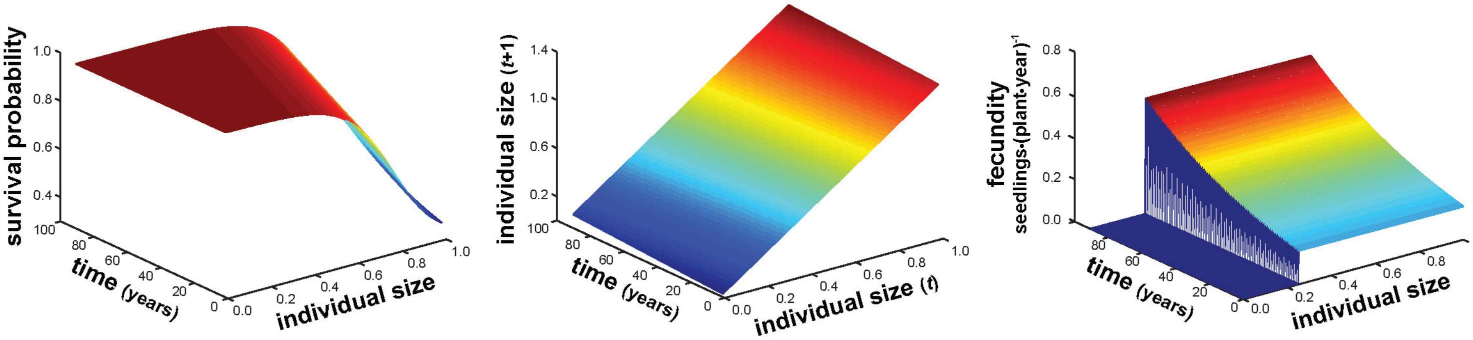

Appendix 3

Vital rates reconstructed for the artificial species

Artificial species 7

Known vital rates

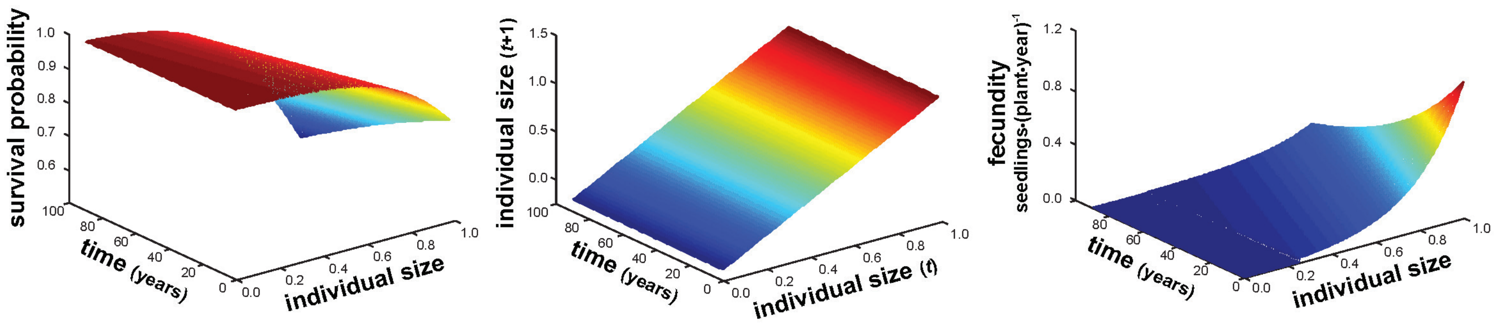

$w = 0$

$r_m = 0.93$

Solution 7.1

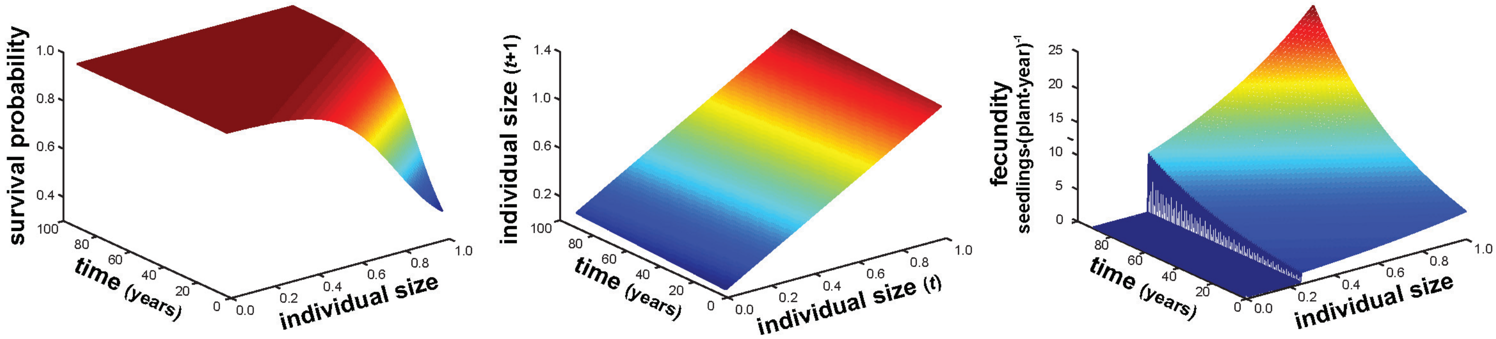

$r_m = 0.81$

Solution 7.2

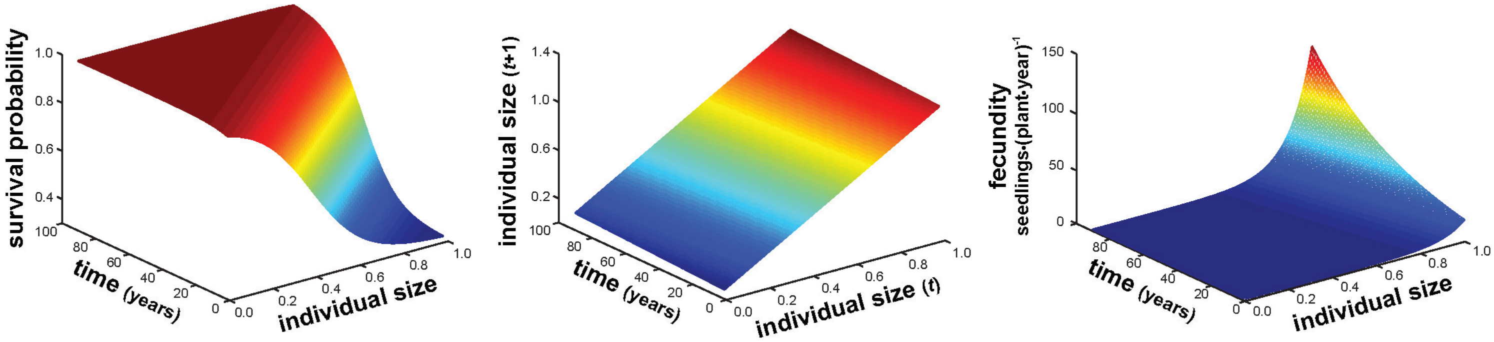

$r_m = 0.55$

Solution 7.3

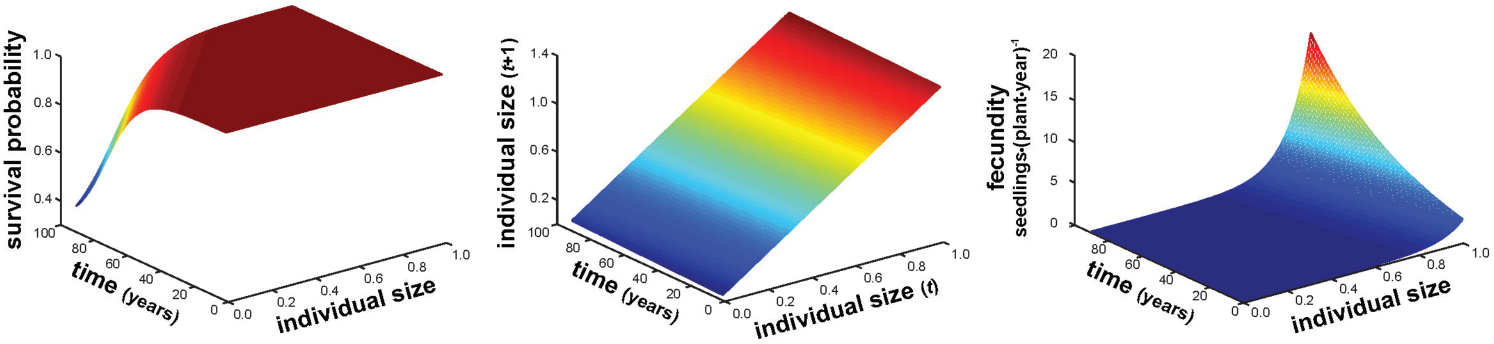

Appendix 3

Vital rates reconstructed for the artificial species

Artificial species 7 (*cont.*)

$w = 1$

$r_m =$ **0.99** Solution 7.4

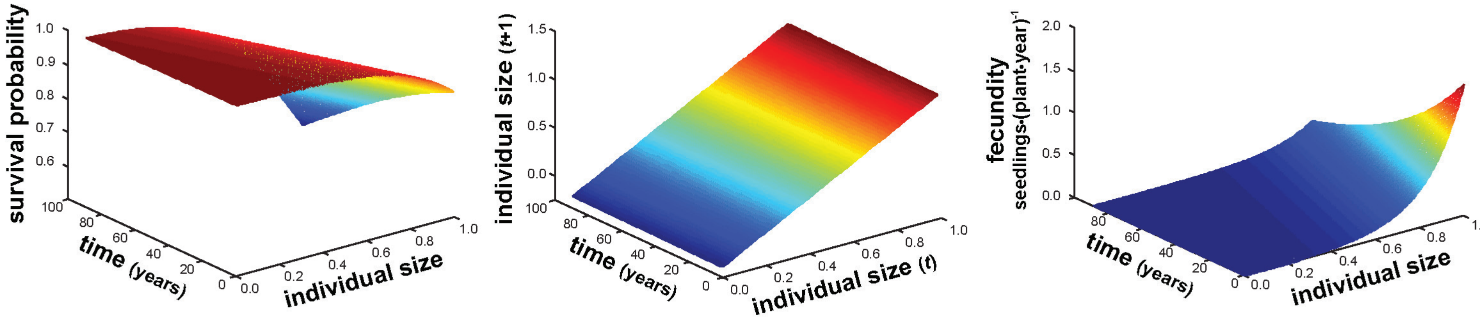

$w = 10$

$r_m = 0.93$ Solution 7.5

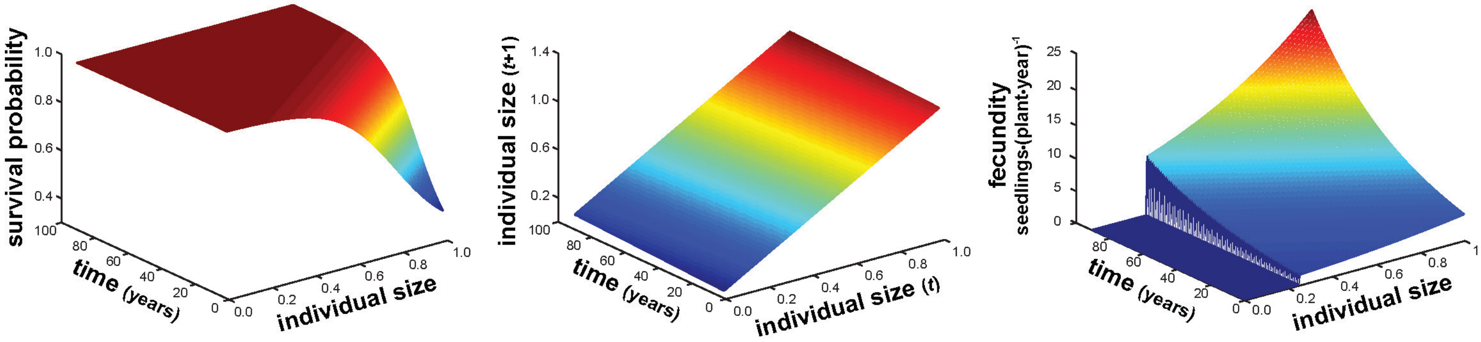

$r_m = 0.81$ Solution 7.6

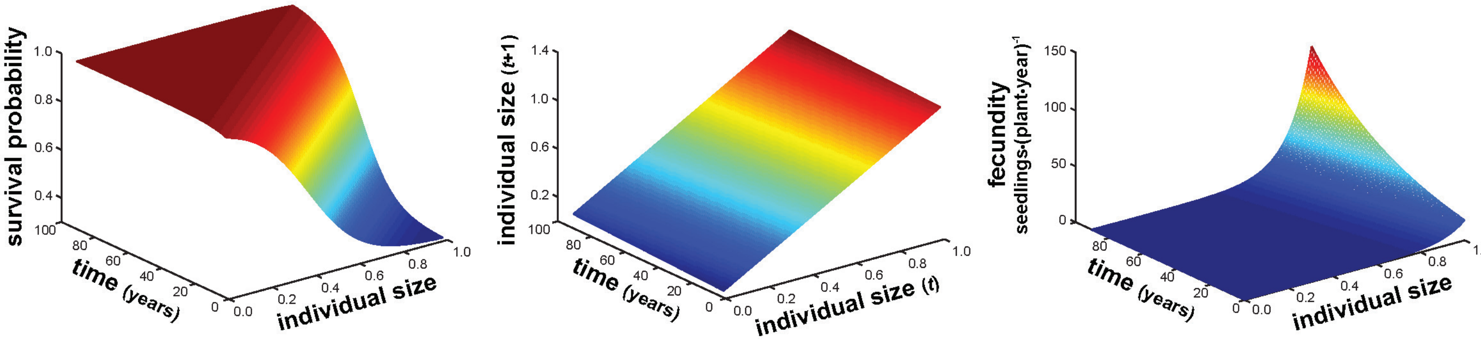

$r_m = 0.55$ Solution 7.7

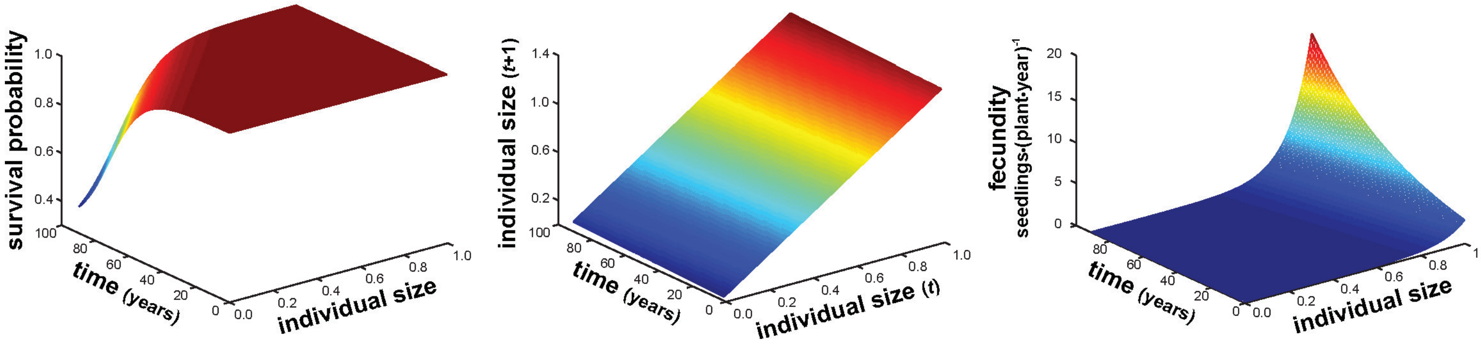

Appendix 3

Vital rates reconstructed for the artificial species

Artificial species 7 (*cont.*)

w = 100

r_m = 0.94

Solution 7.8

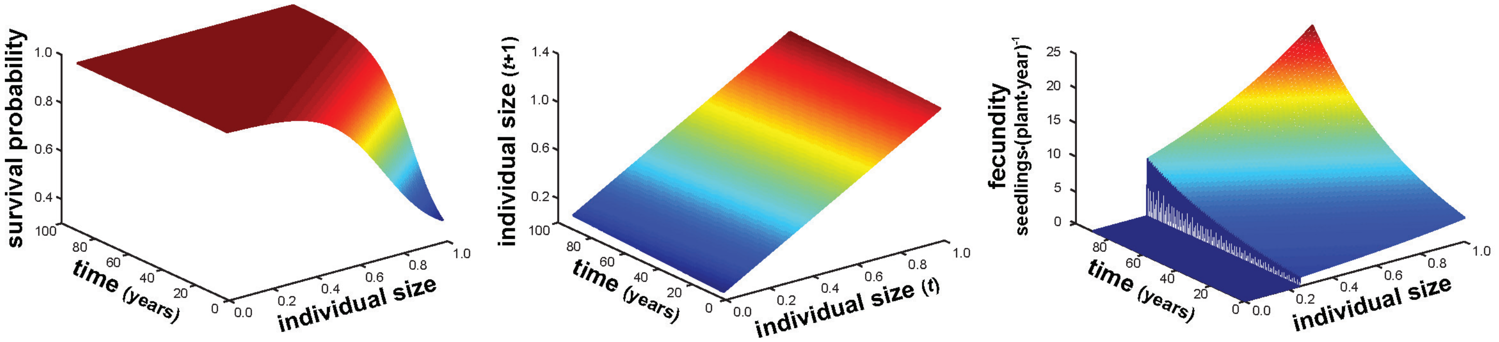

r_m = 0.79

Solution 7.9

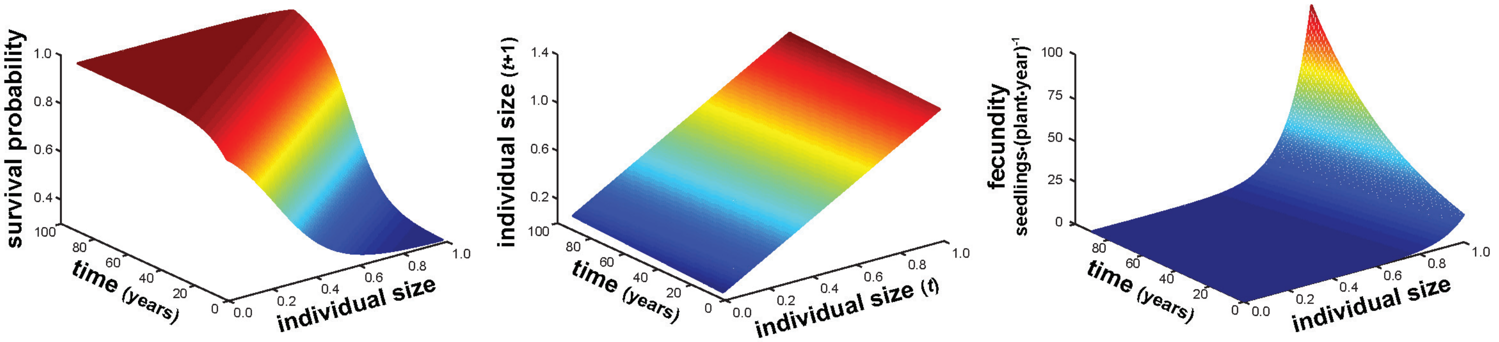

r_m = 0.55

Solution 7.10

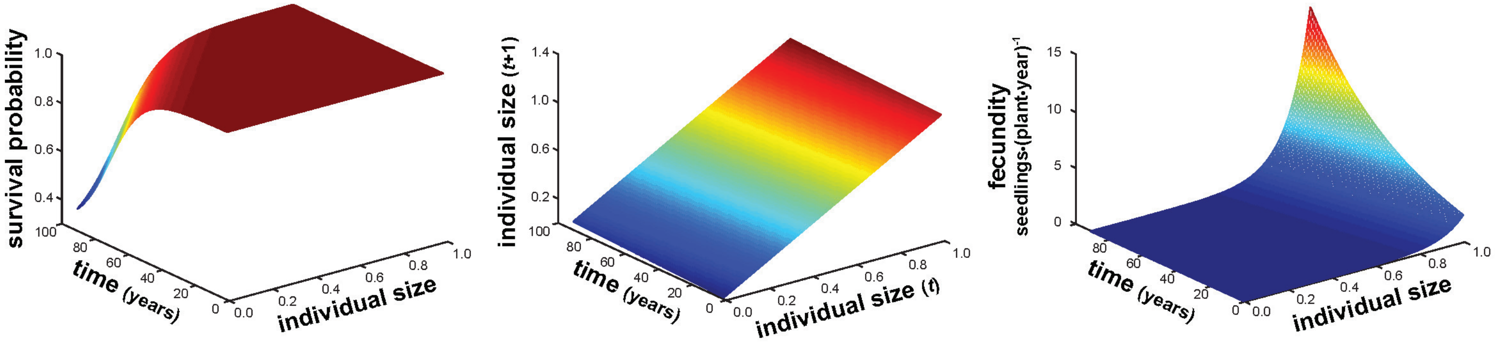

w = 1000

r_m = 0.57

Solution 7.11

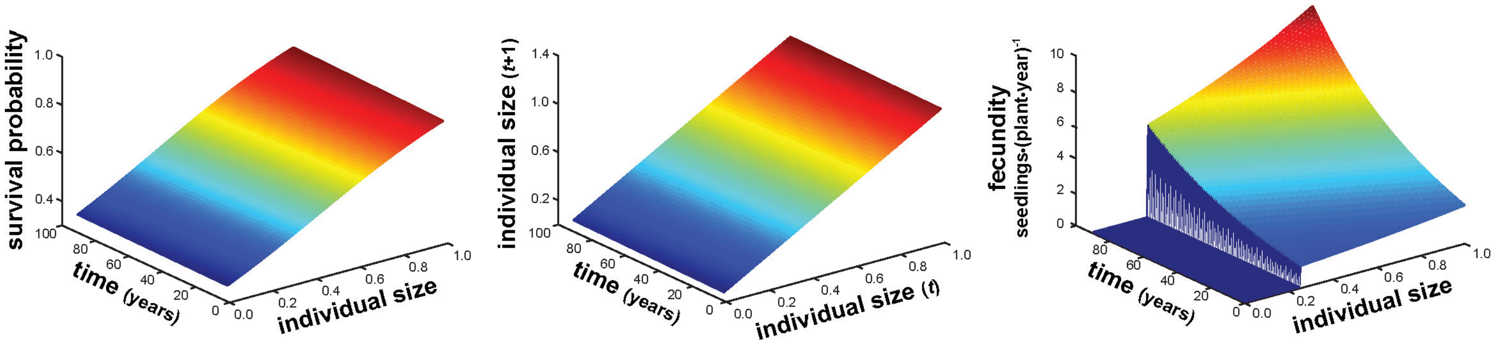

Appendix 3

Vital rates reconstructed for the artificial species

Artificial species 8

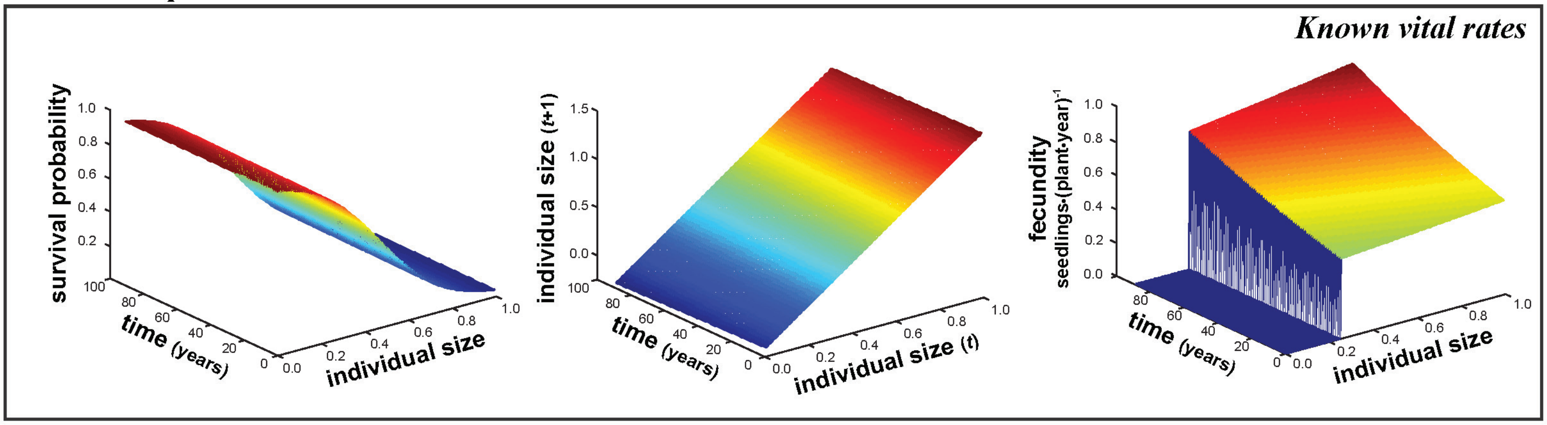

$w = 0$

$r_m = 0.78$

Solution 8.1

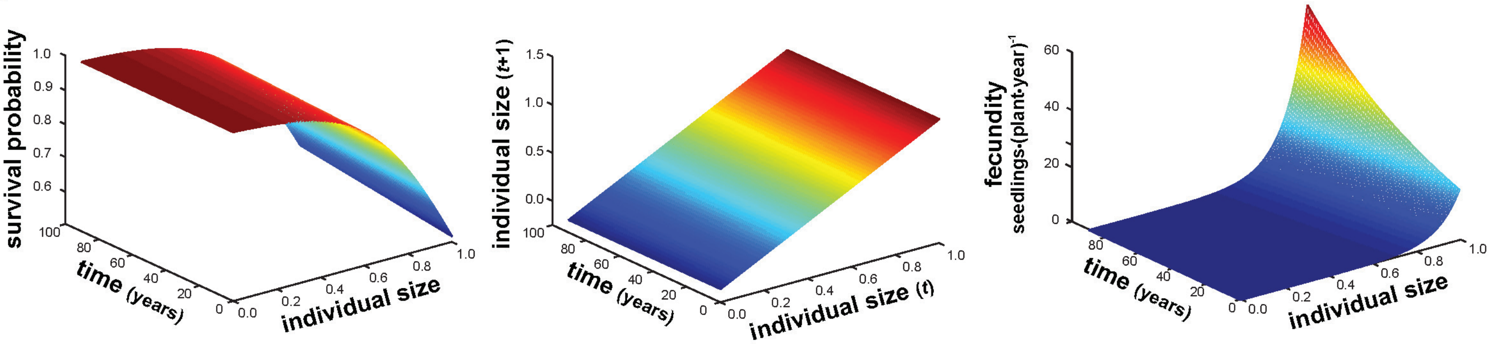

$r_m = 0.49$

Solution 8.2

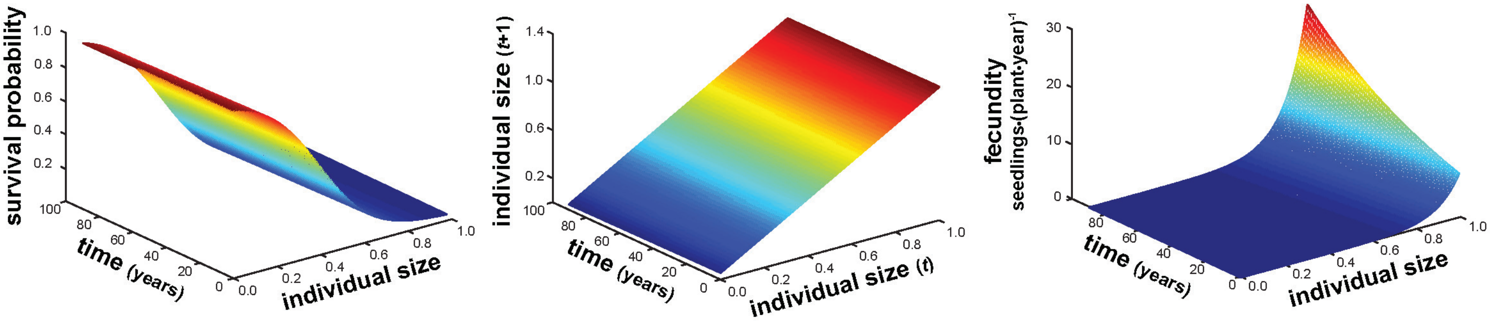

$r_m = 0.40$

Solution 8.3

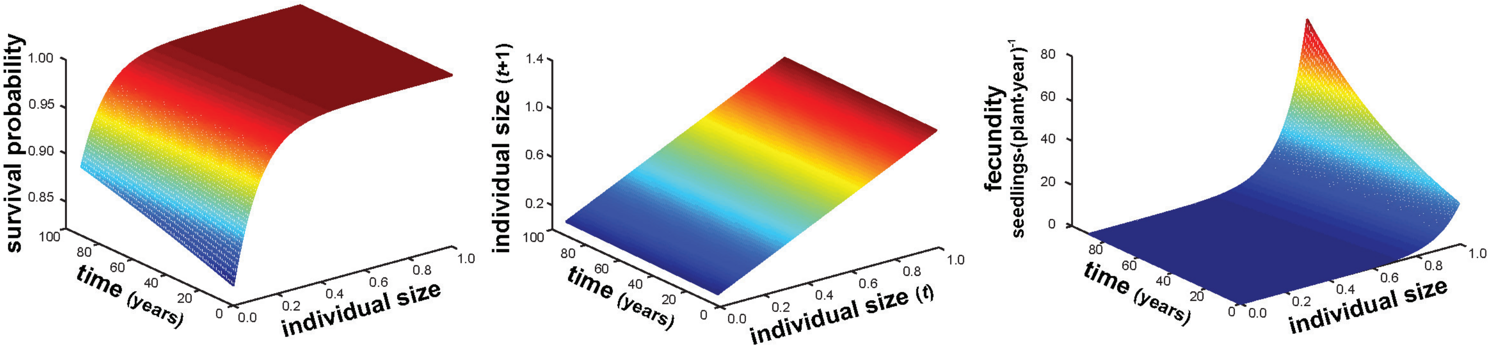

Appendix 3

Vital rates reconstructed for the artificial species

Artificial species 8 (*cont.*)

$w = 0$

$r_m = 0.11$

Solution 8.4

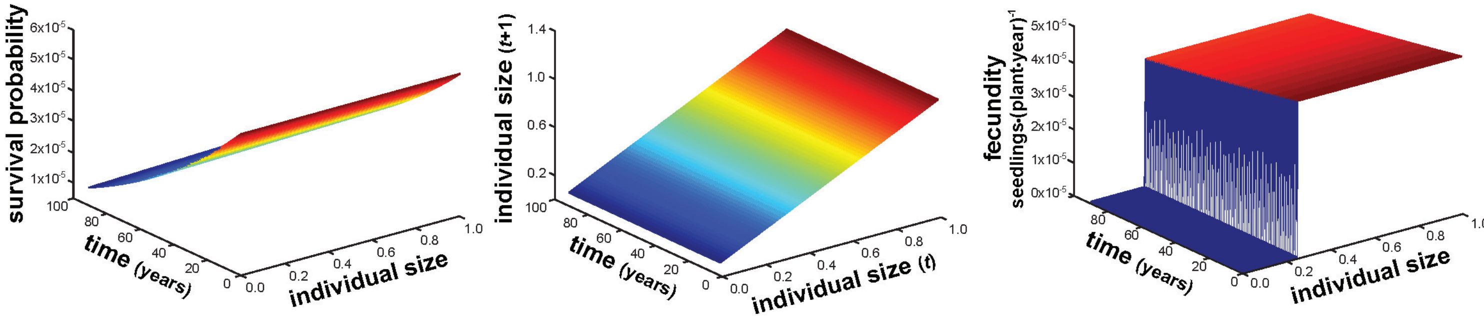

$w = 1$

$r_m = 0.84$

Solution 8.5

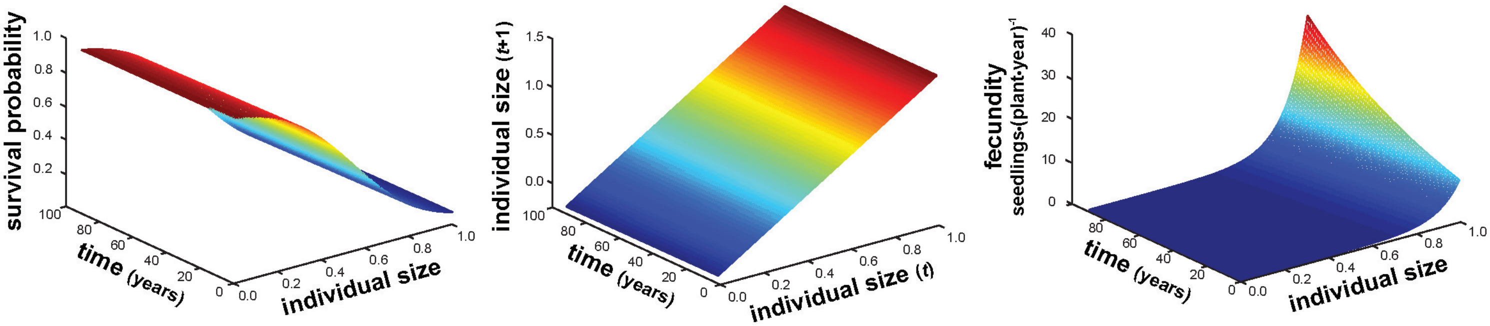

$r_m = 0.65$

Solution 8.6

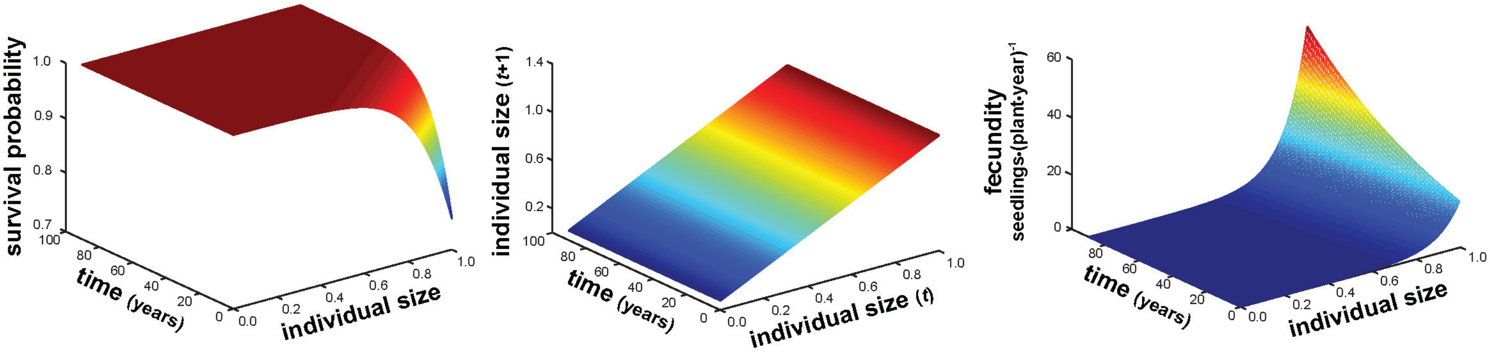

$r_m = 0.36$

Solution 8.7

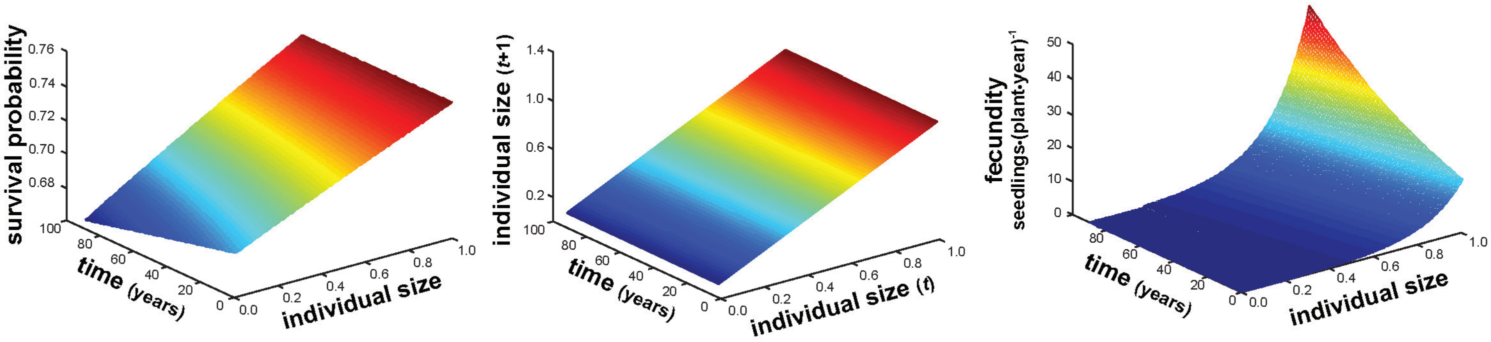

Appendix 3

Vital rates reconstructed for the artificial species

Artificial species 8 (*cont.*)

w = 10

r_m = 0.87

Solution 8.8

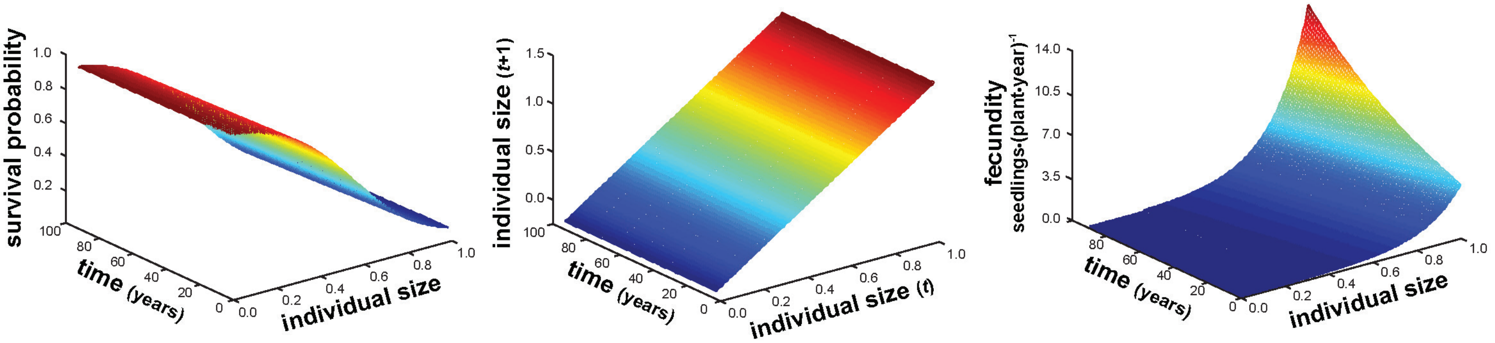

r_m = 0.38

Solution 8.9

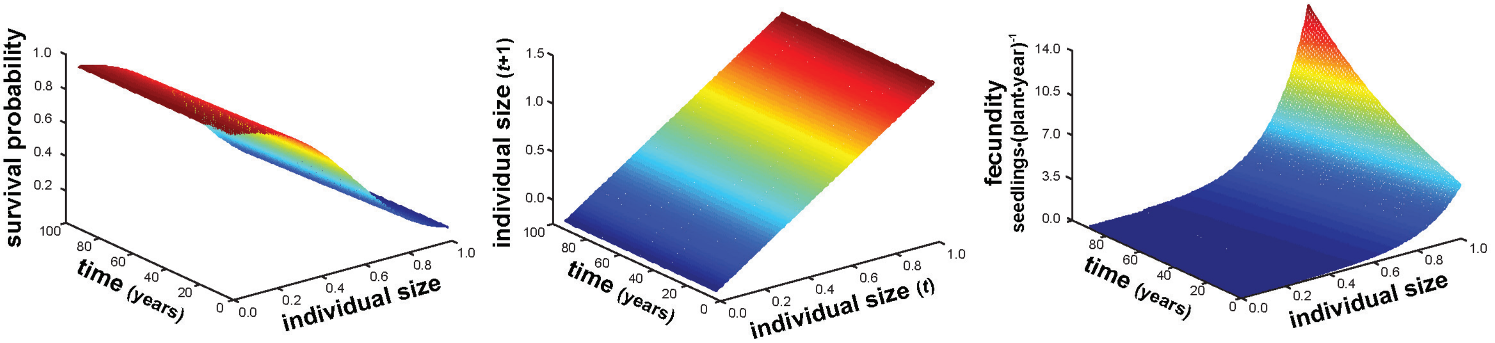

w = 100

r_m = **0.95**

Solution 8.10

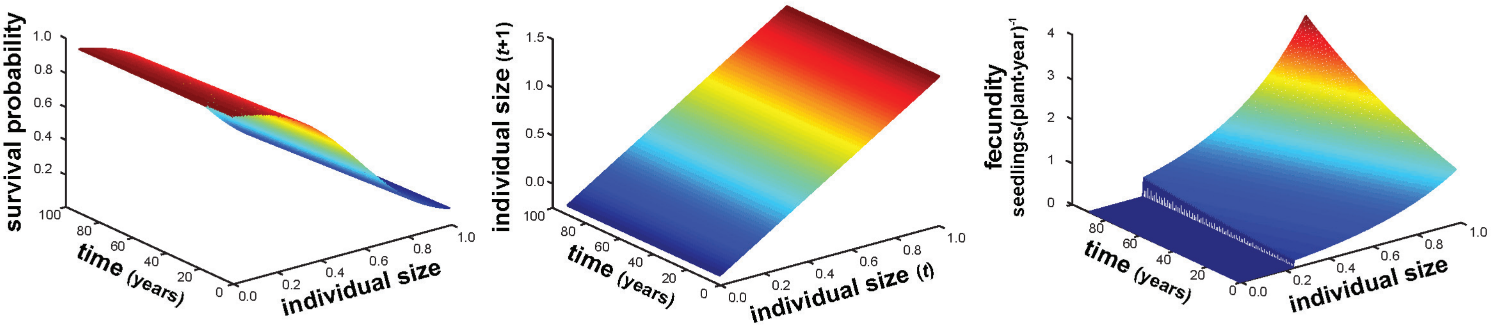

r_m = 0.85

Solution 8.11

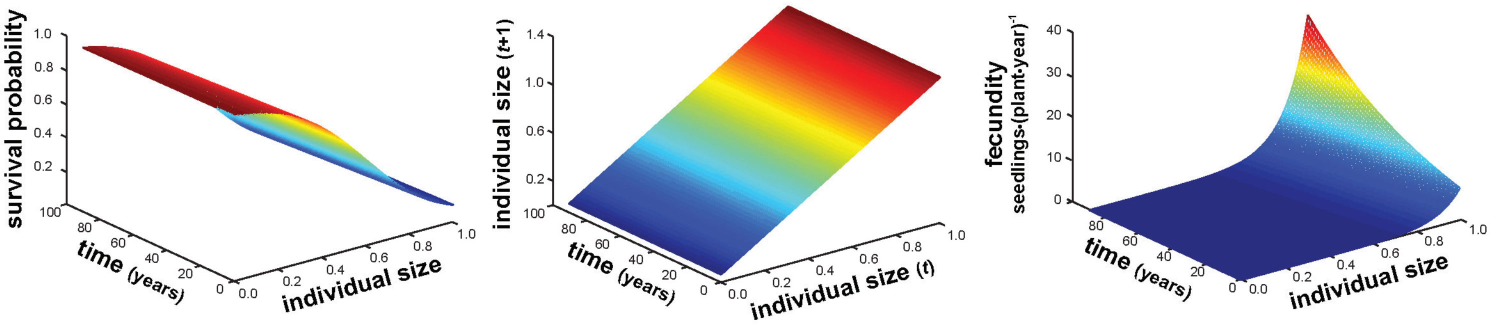

Appendix 3

Vital rates reconstructed for the artificial species

Artificial species 8 (*cont.*)

w = 100

r_m = 0.39

Solution 8.12

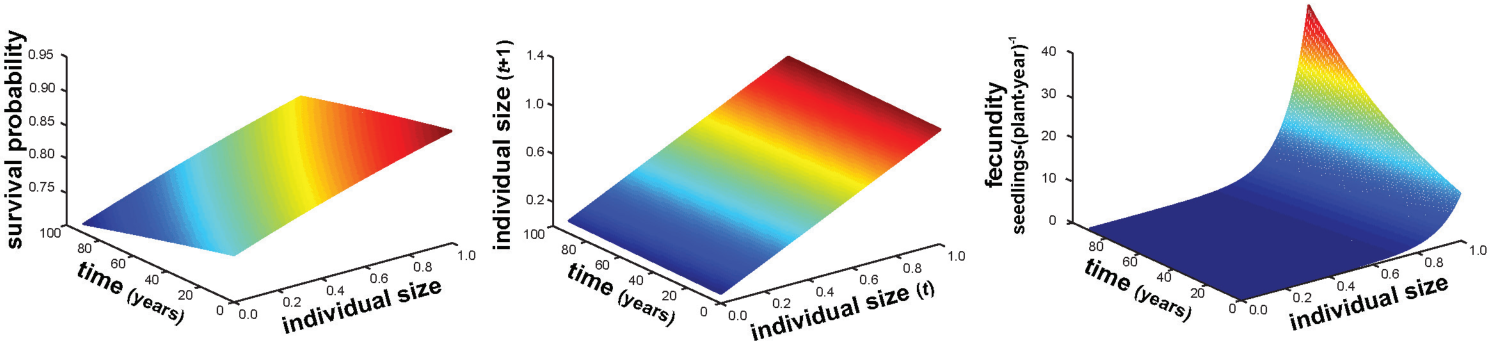

w = 1000

r_m = 0.85

Solution 8.13

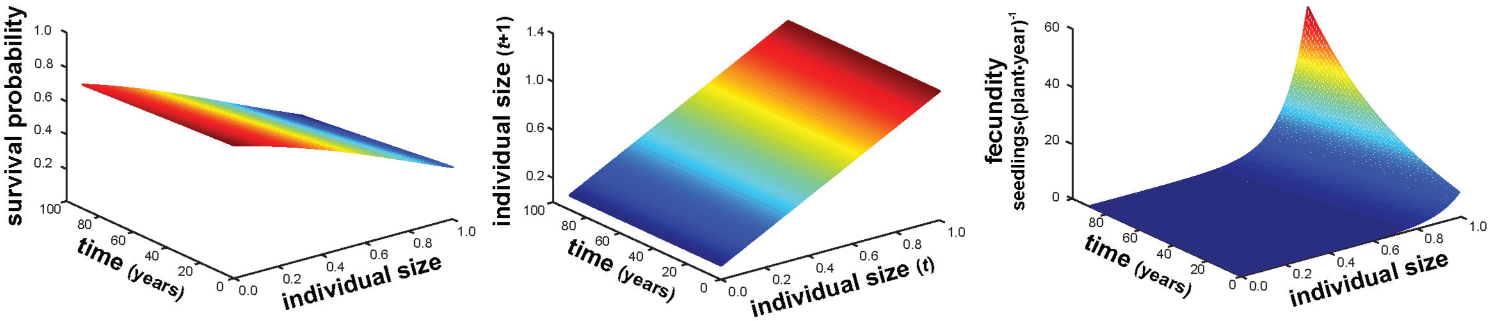

r_m = 0.85

Solution 8.14

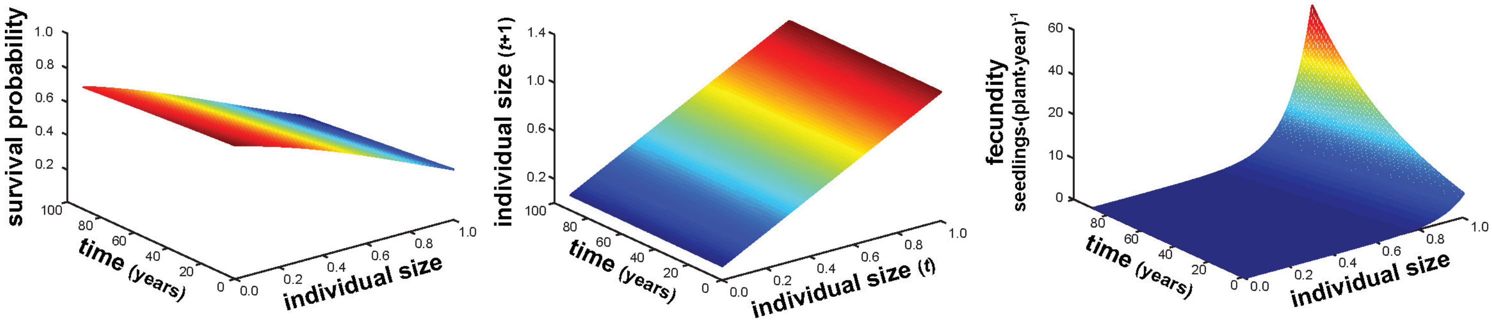

r_m = 0.42

Solution 8.15

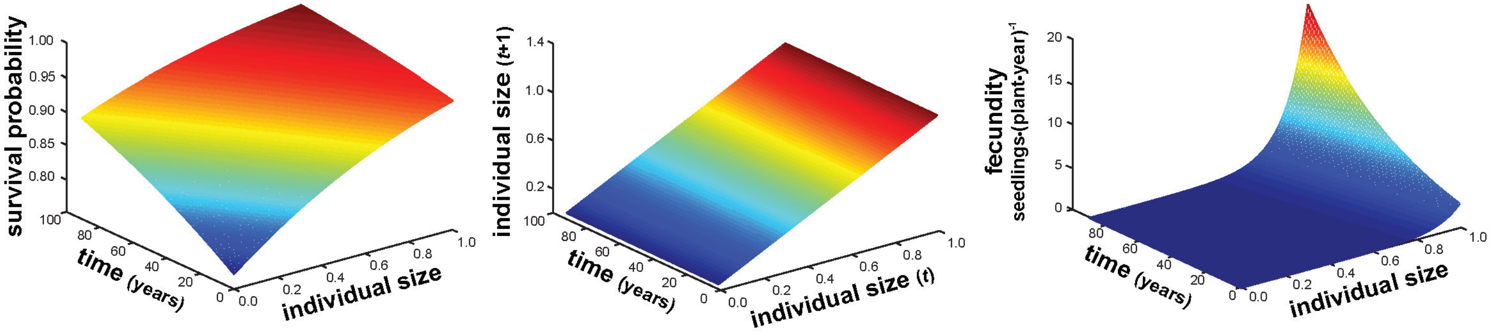

Appendix 3

Vital rates reconstructed for the artificial species

Artificial species 9

Known vital rates

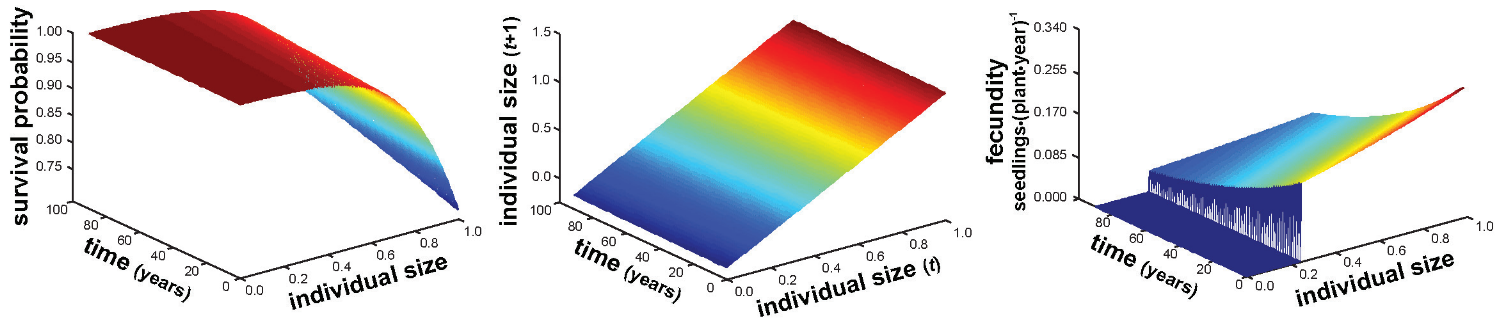

w = 0

r_m = **0.96**

Solution 9.1

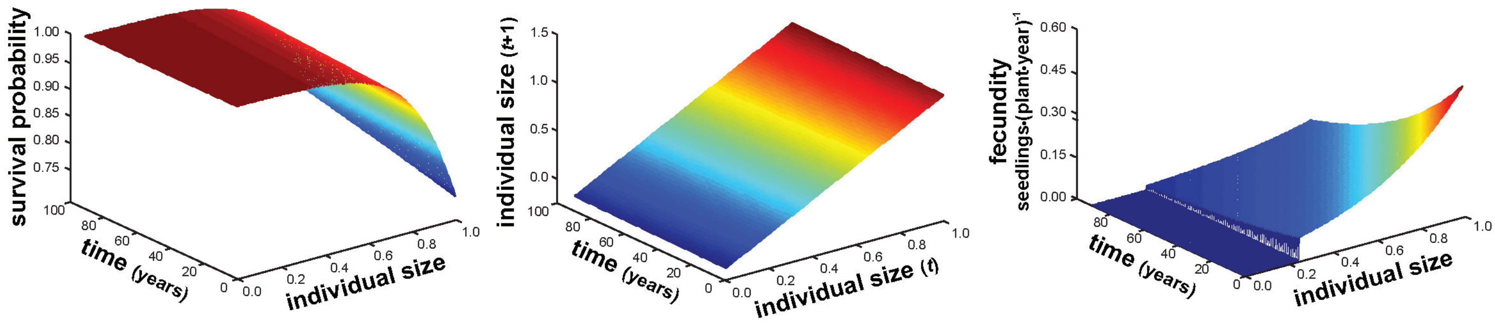

r_m = 0.58

Solution 9.2

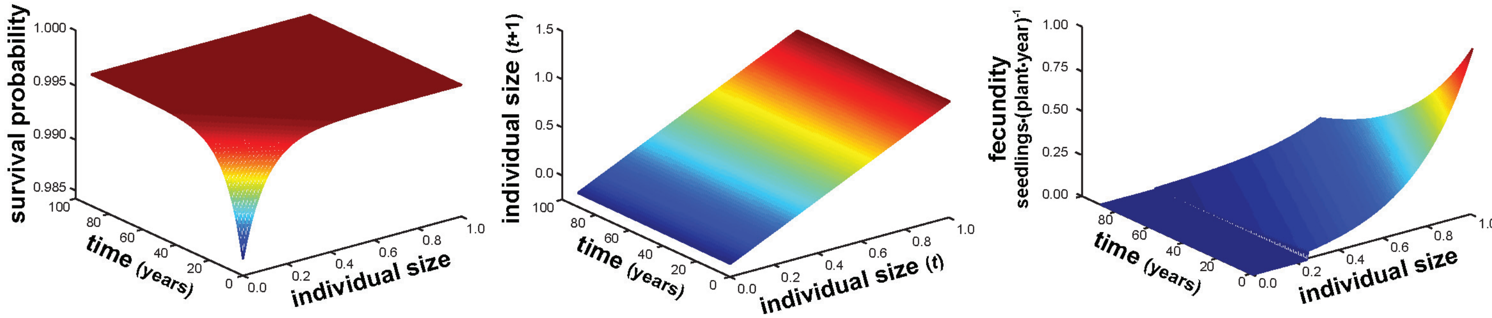

w = 1

r_m = **0.98**

Solution 9.3

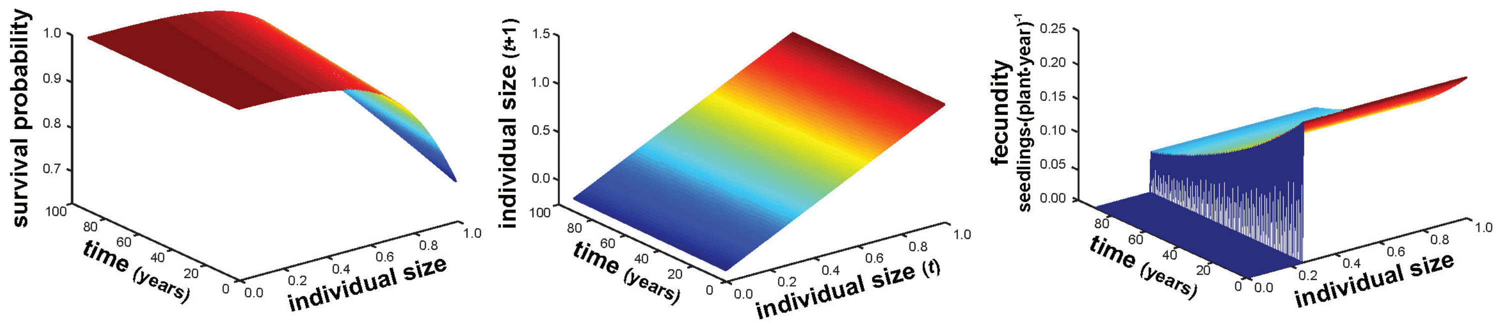

Appendix 3

Vital rates reconstructed for the artificial species

Artificial species 9 (*cont.*)

w = 10

r_m = 0.98 Solution 9.4

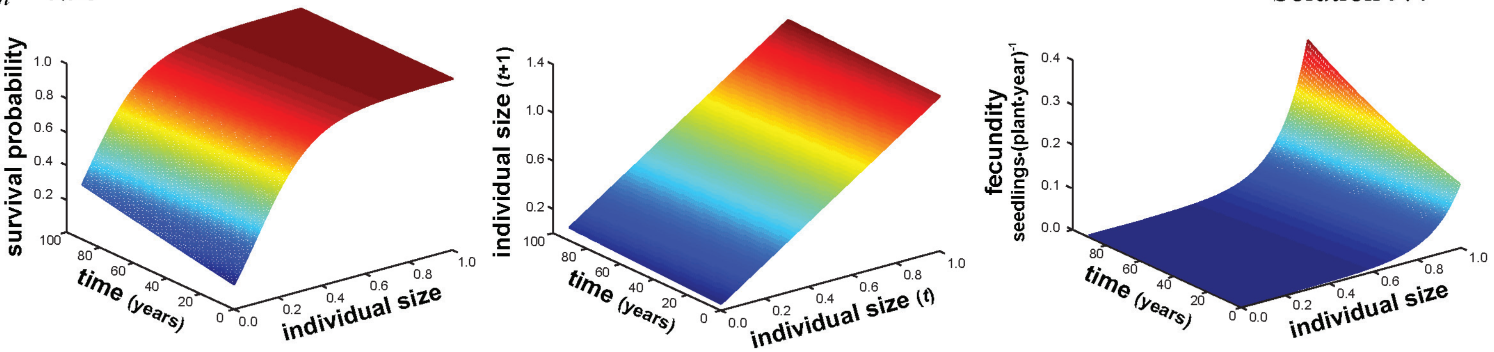

r_m = 0.58 Solution 9.5

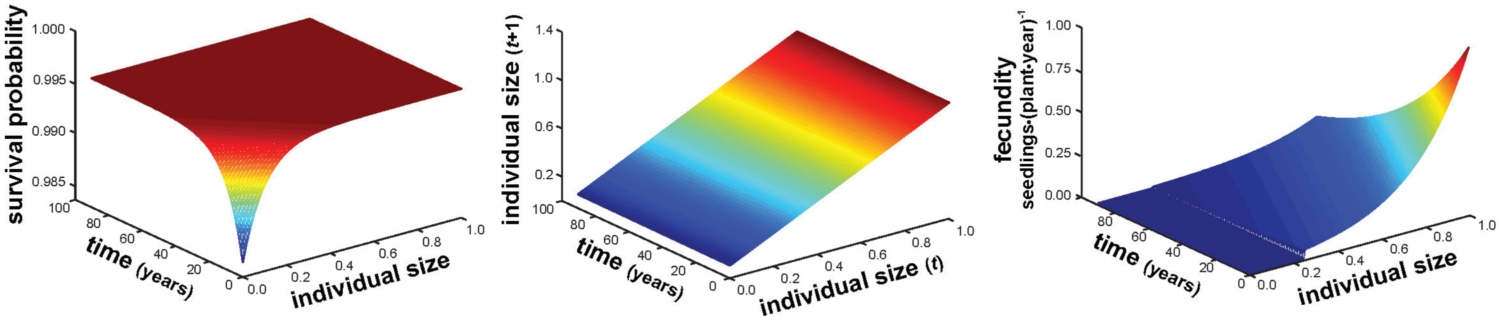

w = 100

r_m = **0.98** Solution 9.6

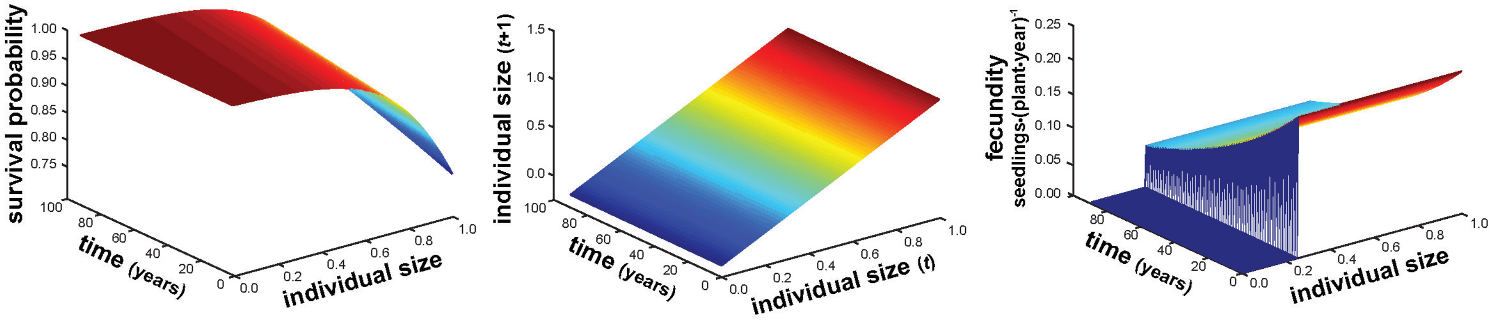

r_m = 0.66 Solution 9.7

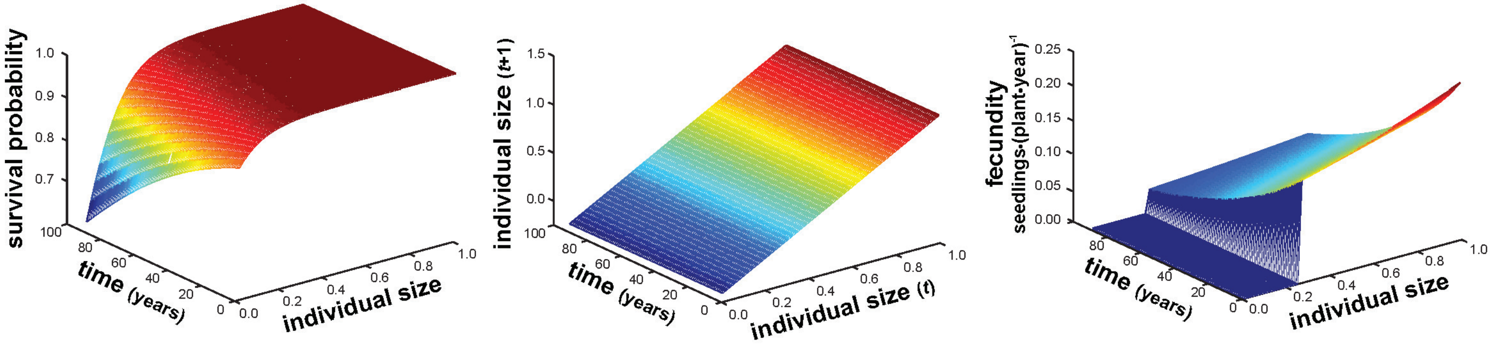

Appendix 3

Vital rates reconstructed for the artificial species

Artificial species 9 (*cont.*)

w = 1000

r_m = **0.98**

Solution 9.8

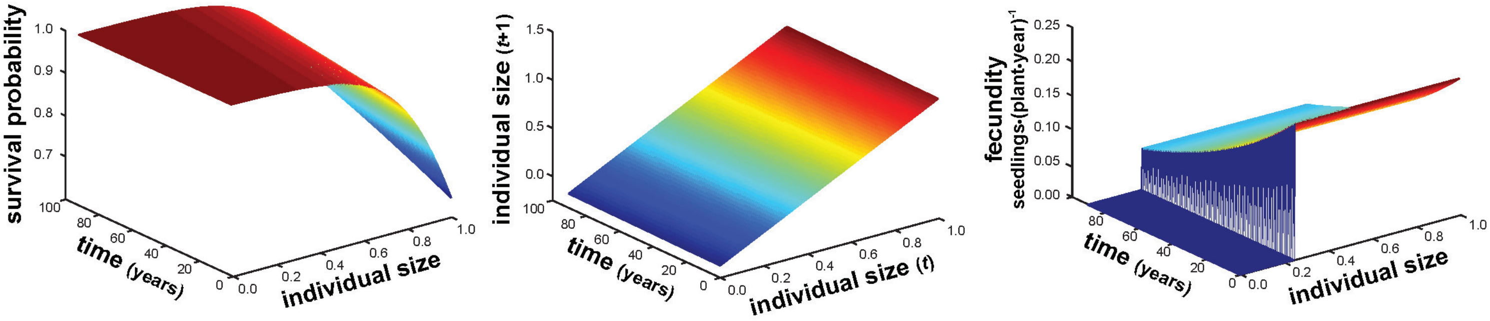

Appendix 3

Vital rates reconstructed for the artificial species

Artificial species 10

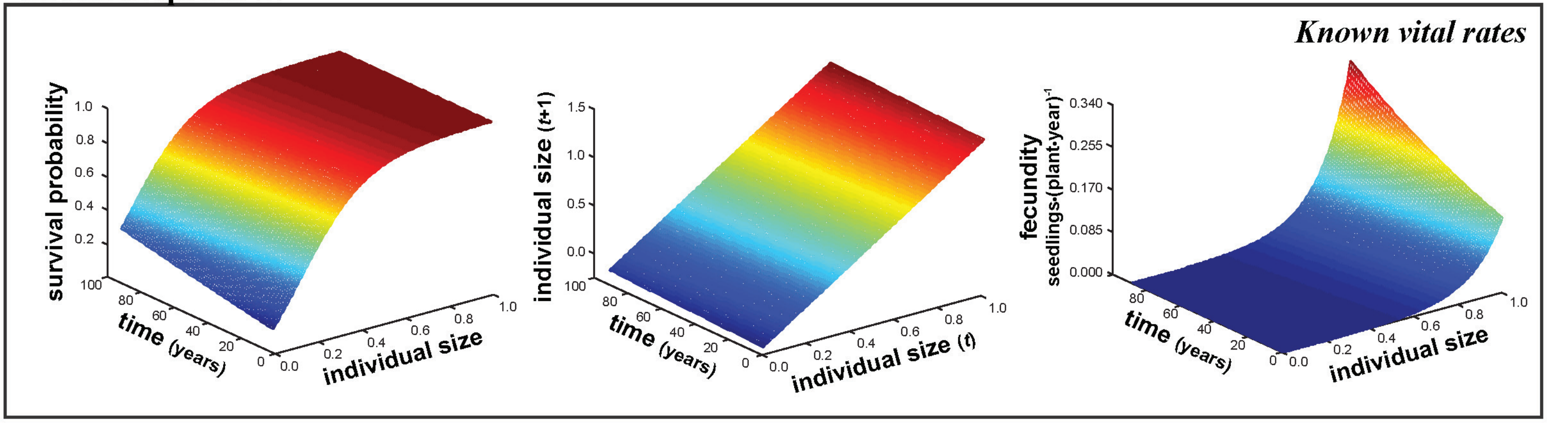

w = 0

r_m = **1.00**

Solution 10.1

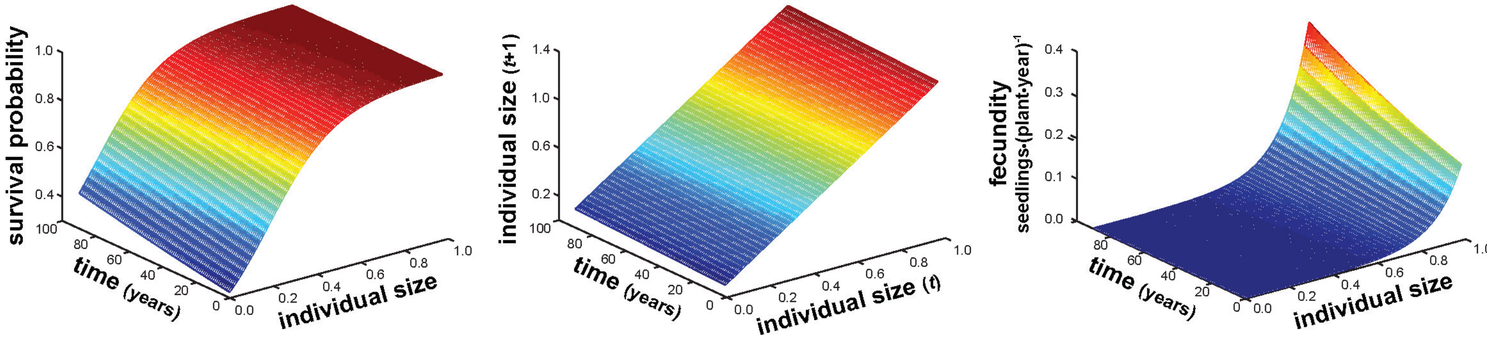

r_m = 0.64

Solution 10.2

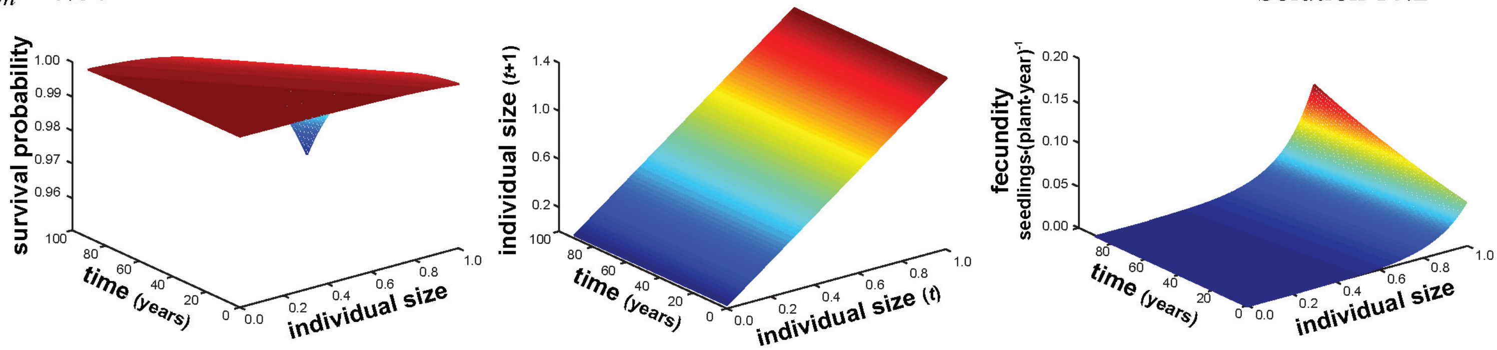

w = 1

r_m = **1.00**

Solution 10.3

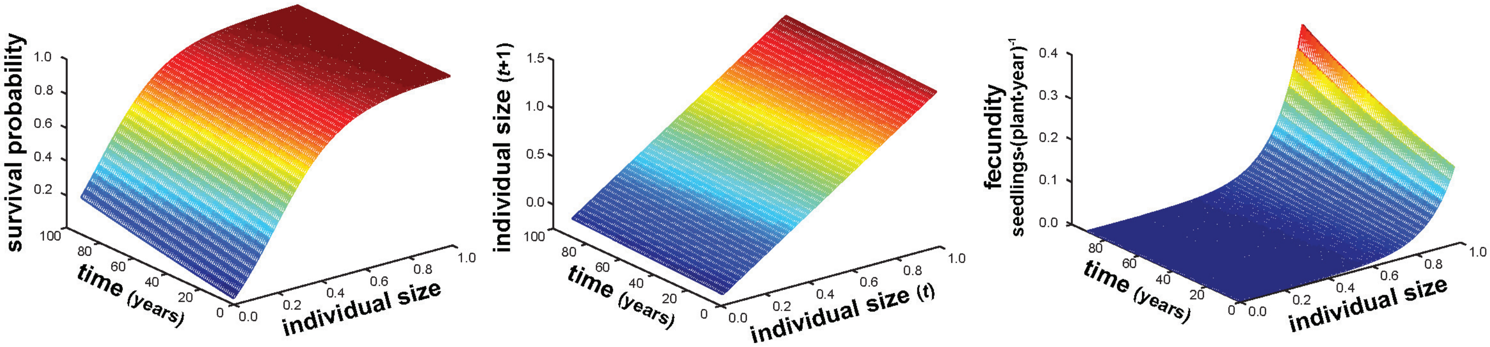

Appendix 3

Vital rates reconstructed for the artificial species

Artificial species 10 (*cont.*)

w = 1

r_m = 0.63

Solution 10.4

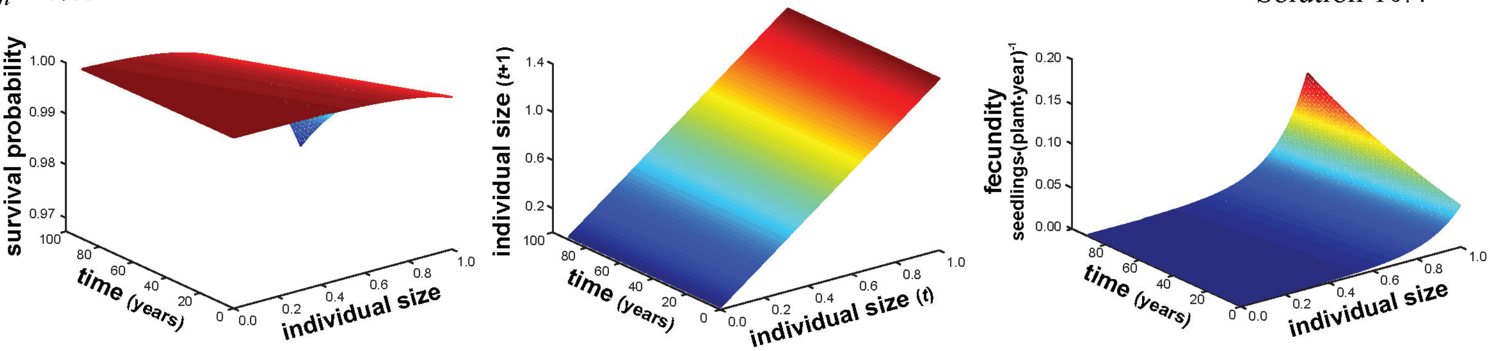

w = 10

r_m = **1.00**

Solution 10.5

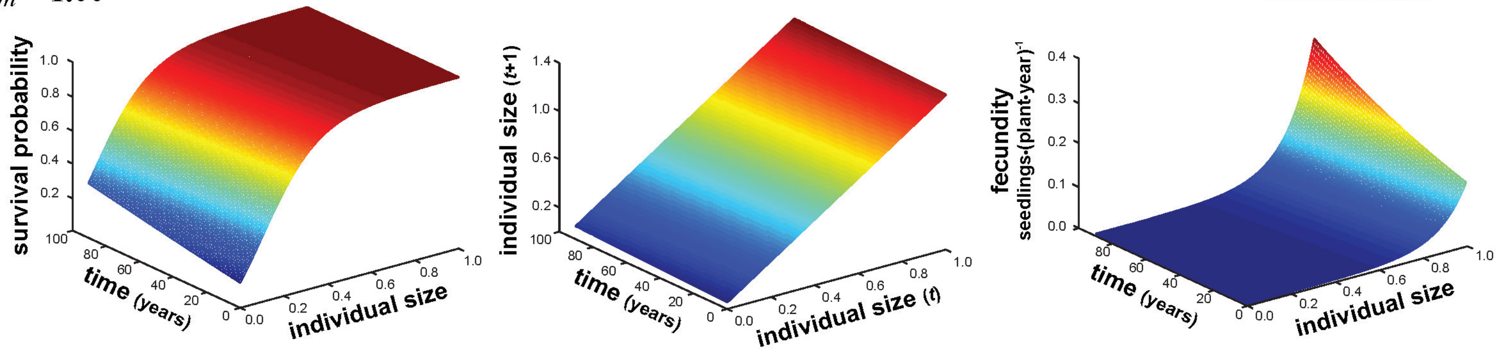

w = 100

r_m = 0.94

Solution 10.6

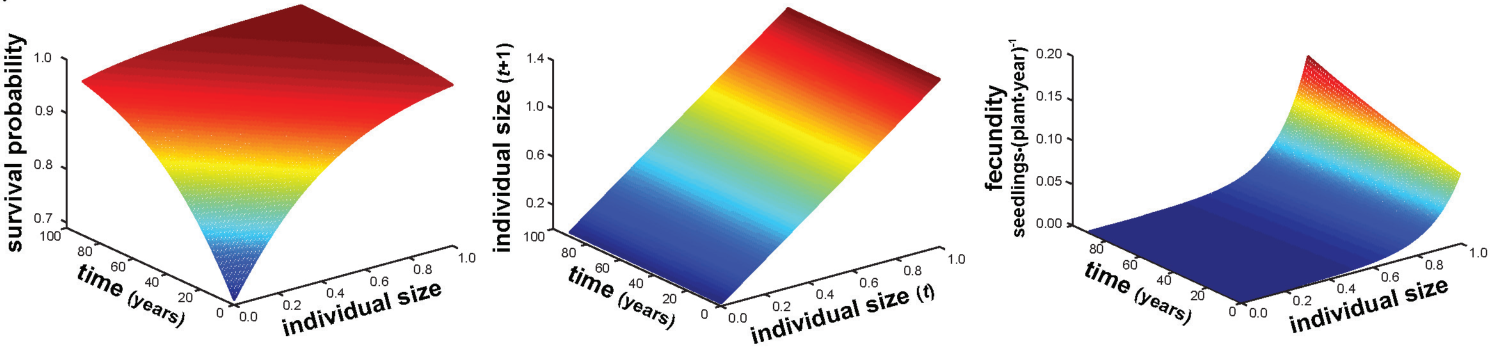

r_m = 0.64

Solution 10.7

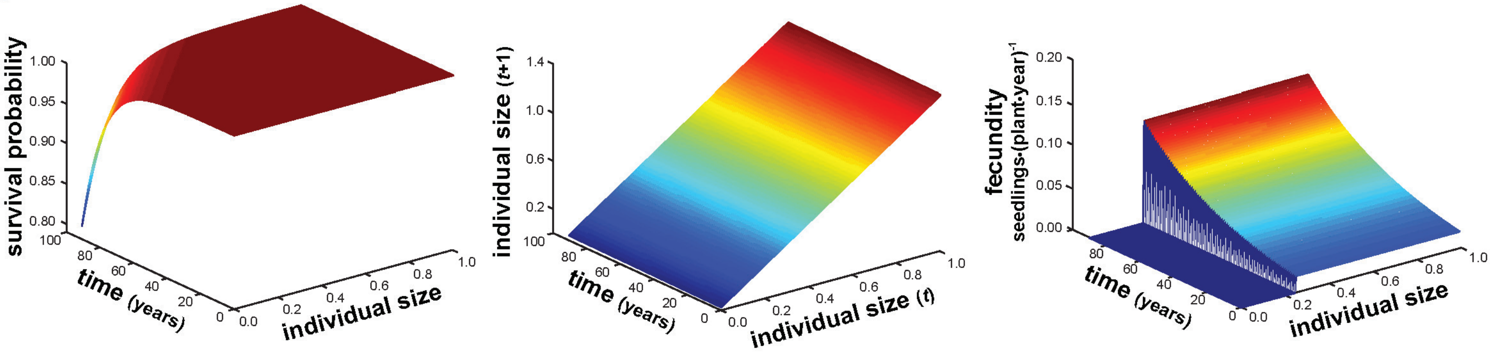

Appendix 3

Vital rates reconstructed for the artificial species

Artificial species 10 (*cont.*)

w = 1000

r_m = 0.64

Solution 10.8

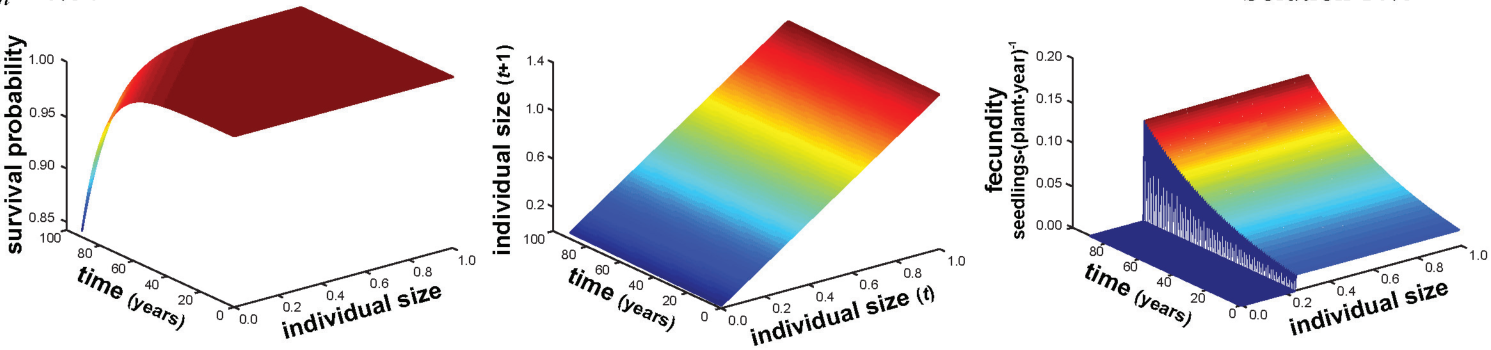

r_m = 0.59

Solution 10.9

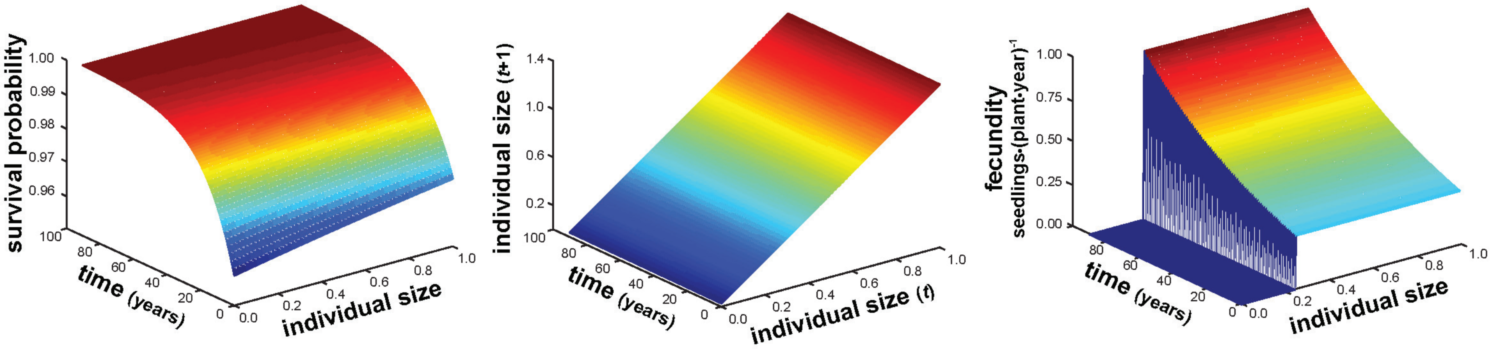

Supplement: Supplementary file 3 [file ece30003-2273-SD3.pdf]
