# Supplementary material for: Reconstructing shifts in vital rates driven by long-term environmental change: a new demographic method based on readily available data
Source: Ecol Evol. 2013 Jun 7;3(7):2273–84. doi: 10.1002/ece3.549 (PMC3728964; doi:10.1002/ece3.549)
Supplement: Supplementary file 4 [file ece30003-2273-SD4.docx]

***Appendix 4***

**Model code for ADMB**

The Automatic Differentiation Model Builder (ADMB) software requires three input files: a .tpl file containing the model code (see below), a .dat file containing the dataset (see DATA_SECTION below for the information to be included in such file), and a .pin file containing the starting point for the exploration of the likelihood surface (a 16-parameter vector). ADMB produces an output of 16 files. For our purposes, we focus on the .rep file, which contains the resulting parameter vector associated with the maximum likelihood (see REPORT_SECTION).

The content of the .tpl file is as follows (comments are preceded by //):

DATA_SECTION // Data included in the .dat file

init_int nt // Number of populations studied, *M*

init_int ni // Size of the partition of the size range *X* in equation A1; in our case 200

init_int nm // Largest sample size, i.e., max{*N*_1_,…, *N_M_*}

init_int minf // Minimum reproductive size

init_number xmin // Smallest size of an individual (observed or biological)

init_number xmax // Largest size of an individual (observed or biological)

init_number factor // Value of *w* in equation A6

init_vector t(1,nt) // Observed times {*T*_1_, …, *T_M_*}

init_vector x(1,ni) // Partition of the size range *X*

init_vector z(1,ni+1) // Class marks of the partition

init_vector dobs(1,nt) // Observed population densities, i.e. {*D*_1_, …, *D_M_*}

init_vector tm(1,nt) // Sample sizes of the populations studied, i.e. {*N*_1_, …, *N_M_*}

init_matrix data(1,nm,1,nt) // Data matrix, i.e. a matrix with columns *X*_1_, …, *X_M_*

PARAMETER_SECTION

init_bounded_number S1(-10,10) // The 16 parameters of the model with their init_bounded_number S2(-0.1,0.1) // associated intervals in parenthesis

init_bounded_number S3(-10,10)

init_bounded_number S4(-0.001,0.001)

init_bounded_number GS(0.001,0.1)

init_bounded_number G1(0,0.1)

init_bounded_number G2(-0.001,0.001)

init_bounded_number G3(0.5,1.5)

init_bounded_number G4(-0.00001,0.00001)

init_bounded_number NS1(-10,0)

init_bounded_number NS2(-0.02,0.02)

init_bounded_number NS3(0,10)

init_bounded_number NS4(-0.0002,0.0002)

init_bounded_number SSS(0.0001,0.1)

init_bounded_number SS1(0,0.01)

init_bounded_number SS2(-0.0001,0.0001)

objective_function_value v

number eval

number lambda

number ssmu

number sumg

number sumss

number temp

number da

number dsigma

number dtemp

number vd

number ve

number ssd

number ssdmu

number corrd

vector colfxy(1,ni)

vector colgxy(1,ni)

vector colpxy(1,ni)

vector ex1(1,ni)

vector gmu(1,ni)

vector inex(1,ni)

vector nsx(1,ni)

vector ssy(1,ni)

vector sumcol(1,nt)

vector sx(1,ni)

vector de(1,nt)

vector dmu(1,nt)

vector dsubst(1,nt)

vector zg(1,ni+1)

vector zss(1,ni+1)

vector zd(1,nt)

vector p(1,16)

vector doscaled(1,nt-1)

vector descaled(1,nt-1)

matrix structures(1,ni,1,nt)

matrix frequencies(1,ni,1,nt)

matrix nxy(1,ni,1,ni)

matrix sxtg(1,ni,1,nt)

matrix sxtp(1,ni,1,nt)

matrix gmg(1,ni,1,nt)

matrix gmp(1,ni,1,nt)

matrix nsxg(1,ni-minf+1,1,nt)

matrix nsxp(1,ni-minf+1,1,nt)

LOCAL_CALCS

int i,j,k;

eval = 0;

for(i = 1; i <= nt; i++)

for(j = 1; j <= tm[i]; j++)

{

if(data[j][i] < xmin)

data[j][i] = xmin;

if(data[j][i] > xmax)

data[j][i] = xmax;

}

for(i = 1; i <= nt; i++)

for(k = 1; k <= ni; k++)

frequencies[k][i] = 0;

for(i = 1; i <= nt; i++)

for(j = 1; j <= tm[i]; j++)

{

if(data[j][i] >= z[1] && data[j][i] <= z[2])

frequencies[1][i] = frequencies[1][i]+1;

for(k = 2; k <= ni; k++)

if(data[j][i] > z[k] && data[j][i] <= z[k+1])

frequencies[k][i] = frequencies[k][i]+1;

}

END_CALCS

PROCEDURE_SECTION

int i,j,k,l,ti,tj;

eval = eval+1;

de[1] = 1;

dtemp = de[1];

for(i = 1; i < minf; i++)

nsx[i] = 0;

for(i = 1; i <= ni; i++)

ex1[i] = 1;

j = (int)t[1];

sx = elem_div(exp(S1+S2*j+S3*x+S4*j*x),1+exp(S1+S2*j+S3*x+S4*j*x));

for(k = minf; k <= ni; k++)

nsx[k] = exp(NS1+NS2*j+NS3*x[k]+NS4*j*x[k]);

ssmu = SS1+SS2*j;

zss = (z-ssmu)/SSS;

for(k = 1; k <= ni; k++)

ssy[k] = cumd_norm(zss[k+1])-cumd_norm(zss[k]);

sumss = sum(ssy);

ssy = ssy/sumss;

for(k = 1; k <= ni; k++)

{

gmu[k] = G1+G2*j+G3*x[k]+G4*j*x[k];

zg = (z-gmu[k])/GS;

for(l = 1; l <= ni; l++)

colgxy[l] = cumd_norm(zg[l+1])-cumd_norm(zg[l]);

sumg = sum(colgxy);

colgxy = colgxy/sumg;

colpxy = sx[k]*colgxy;

colfxy = nsx[k]*ssy;

nxy.colfill(k,colpxy+colfxy);

}

for(i = 1; i <= 8; i++)

nxy = nxy*nxy;

ex1 = nxy*ex1;

for(j = 1; j <= ni; j++)

if(ex1[j] <= 0)

ex1[j] = 1e-323;

ex1 = ex1/sum(ex1);

inex = ex1;

structures.colfill(1,inex);

for(i = 1; i <= nt-1; i++)

{

ti = (int)t[i];

tj = (int)t[i+1];

for(j = ti; j < tj; j++)

{

sx = elem_div(exp(S1+S2*j+S3*x+S4*j*x),1+exp(S1+S2*j+S3*x+S4*j*x));

for(k = minf; k <= ni; k++)

nsx[k] = exp(NS1+NS2*j+NS3*x[k]+NS4*j*x[k]);

ssmu = SS1+SS2*j;

zss = (z-ssmu)/SSS;

for(k = 1; k <= ni; k++)

ssy[k] = cumd_norm(zss[k+1])-cumd_norm(zss[k]);

sumss = sum(ssy);

ssy = ssy/sumss;

for(k = 1; k <= ni; k++)

{

gmu[k] = G1+G2*j+G3*x[k]+G4*j*x[k];

zg = (z-gmu[k])/GS;

for(l = 1; l <= ni; l++)

colgxy[l] = cumd_norm(zg[l+1])-cumd_norm(zg[l]);

sumg = sum(colgxy);

colgxy = colgxy/sumg;

colpxy = sx[k]*colgxy;

colfxy = nsx[k]*ssy;

nxy.colfill(k,colpxy+colfxy);

}

inex = nxy*inex;

lambda = sum(inex);

dtemp = lambda*dtemp;

inex = inex/lambda;

}

for(j = 1; j <= ni; j++)

if(inex[j] <= 0)

inex[j] = 1e-323;

de[i+1] = dtemp;

if(de[i+1] <= 0)

de[i+1] = 1e-323;

structures.colfill(i+1,inex);

}

da = sum(log(dobs)-log(de))/nt;

dmu = da+log(de);

dsubst = log(dobs)-dmu;

dsigma = sqrt(sum(elem_prod(dsubst,dsubst))/(nt-1));

zd = (dsubst)/dsigma;

vd = 0;

for(i = 1; i <= nt; i++)

vd = vd+log(500/dsigma*(cumd_norm(zd[i]+0.001)-cumd_norm(zd[i]-0.001)));

ve = sum(colsum(elem_prod(frequencies,log(structures))));

v = -ve-factor*vd;

REPORT_SECTION // variables preceded by "report <<" are included in the output file .rep

int i;

report << S1 << endl; // parameter vector that maximizes the likelihood of the data

report << S2 << endl;

report << S3 << endl;

report << S4 << endl;

report << GS << endl;

report << G1 << endl;

report << G2 << endl;

report << G3 << endl;

report << G4 << endl;

report << NS1 << endl;

report << NS2 << endl;

report << NS3 << endl;

report << NS4 << endl;

report << SSS << endl;

report << SS1 << endl;

report << SS2 << endl;

report << endl;

for(i = 1; i <= nt; i++)

report << de[i] << endl; // densities obtained by the model

report << endl;

report << ve << endl; // maximum likelihood of the observed size-structures (eqn. A4)

report << vd << endl; // maximum likelihood of the observed densities (eqn. A5)

report << -v << endl; // composite maximum likelihood (eqn. A6)

report << endl;

ssd = 0;

for(i = 2; i <= nt; i++)

{

doscaled[i-1] = dobs[i]/dobs[1];

descaled[i-1] = de[i];

}

ssd = norm2(doscaled-descaled);

report << ssd << endl; // sum of squares between the observed and modelled densities report << endl;

corrd = 0;

for(i = 1; i <= nt-1; i++)

corrd = corrd+(doscaled[i]-mean(doscaled))*(descaled[i]-mean(descaled));

corrd = corrd/sqrt(norm2(doscaled-mean(doscaled))*norm2(descaled-mean(descaled)));

report << corrd << endl; // correlation between the observed and modelled densities

FINAL_SECTION
