# Supplementary material for: Reconstructing shifts in vital rates driven by long-term environmental change: a new demographic method based on readily available data
Source: Ecol Evol. 2013 Jun 7;3(7):2273–84. doi: 10.1002/ece3.549 (PMC3728964; doi:10.1002/ece3.549)

Appendix 5

Vital rates reconstructed for *Mammillaria dixanthocentron*

Solutions obtained by the model with different values of the coefficient weighting the fitting of the population densities vs. that of the size structures (w in equation 4). r_d is the Pearson correlation coefficient between the observed and reconstructed population densities. Bold r_d: correct reconstruction, roman r_d: incorrect solution that could be discarded (type-1 solution).

$w = 0$

$r_m = 0.63$

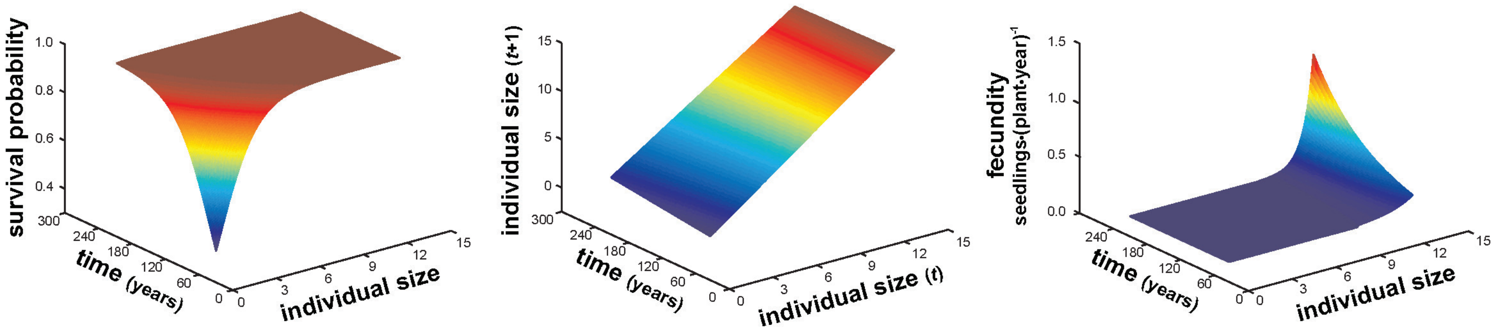

$r_m = 0.22$

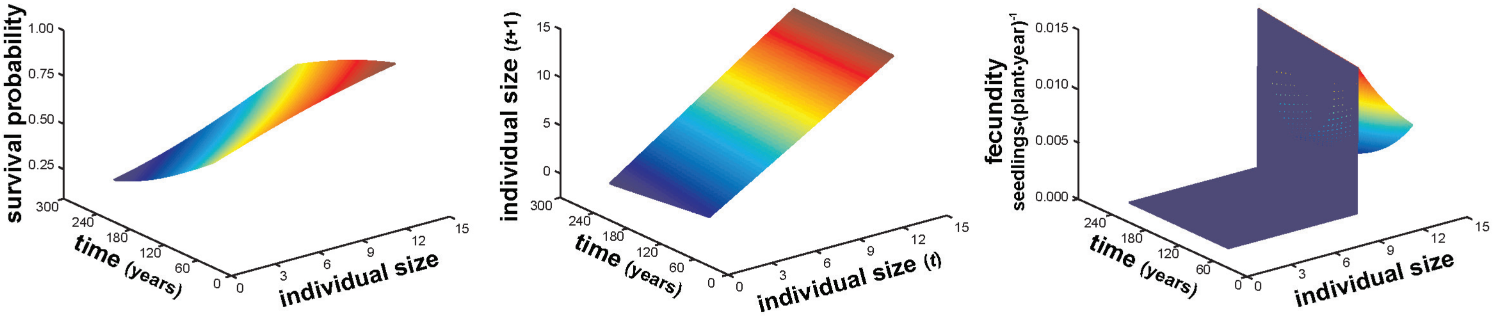

$r_m = 0.22$

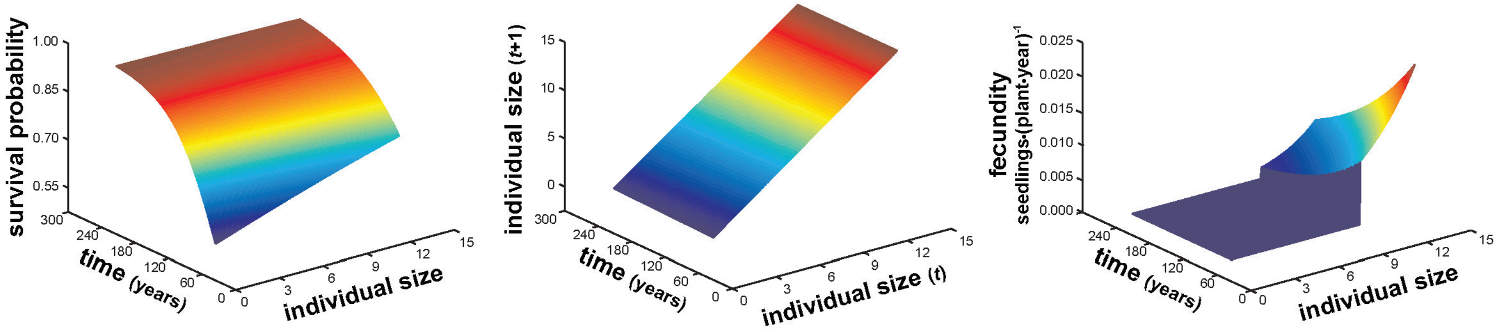

$w = 1$

$r_m = 0.73$

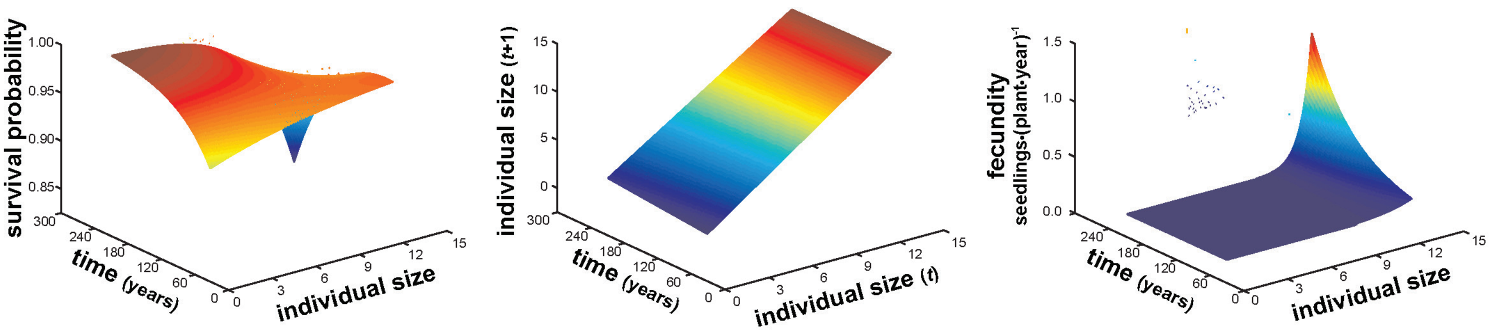

Appendix 5

Vital rates reconstructed for *Mammillaria dixanthocentron* (*cont.*)

$w = 1$

$r_d = 0.61$

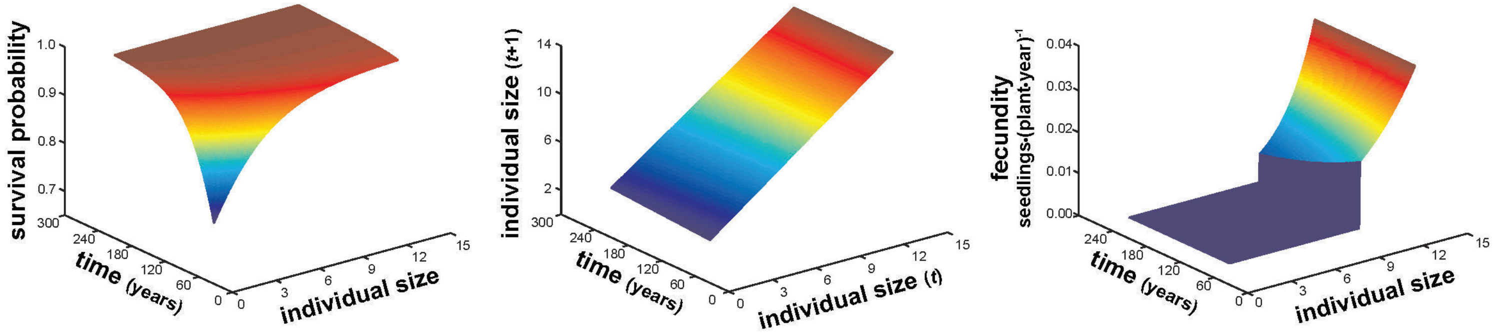

$w = 100$

$r_d = 0.81$

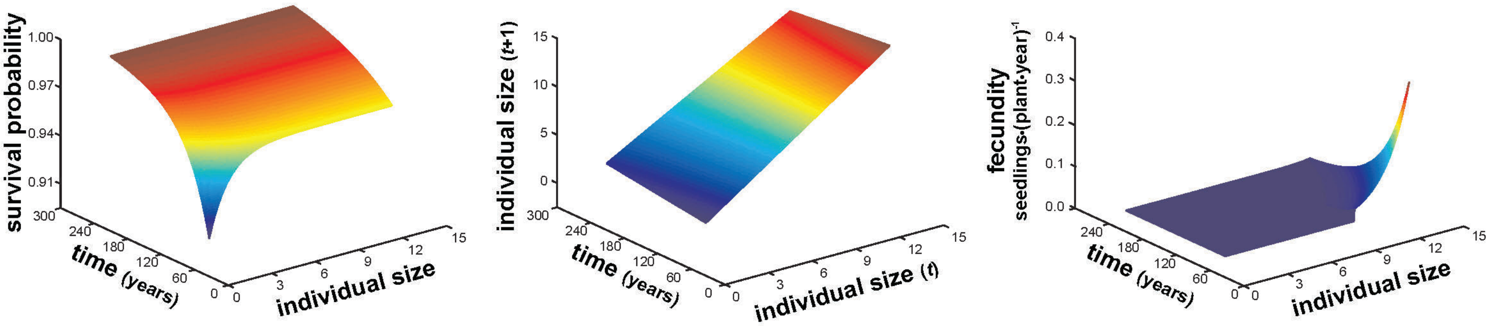

$r_d =$ **0.81**

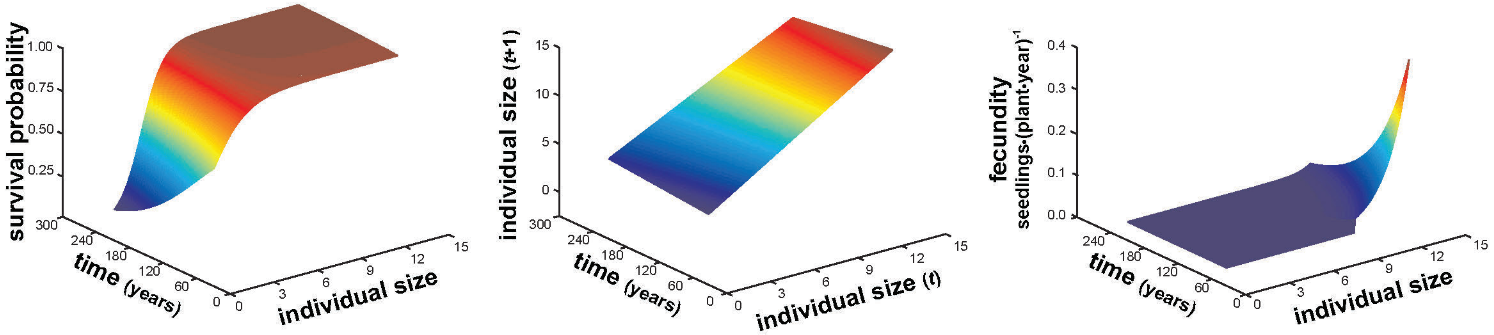

$r_d = 0.80$

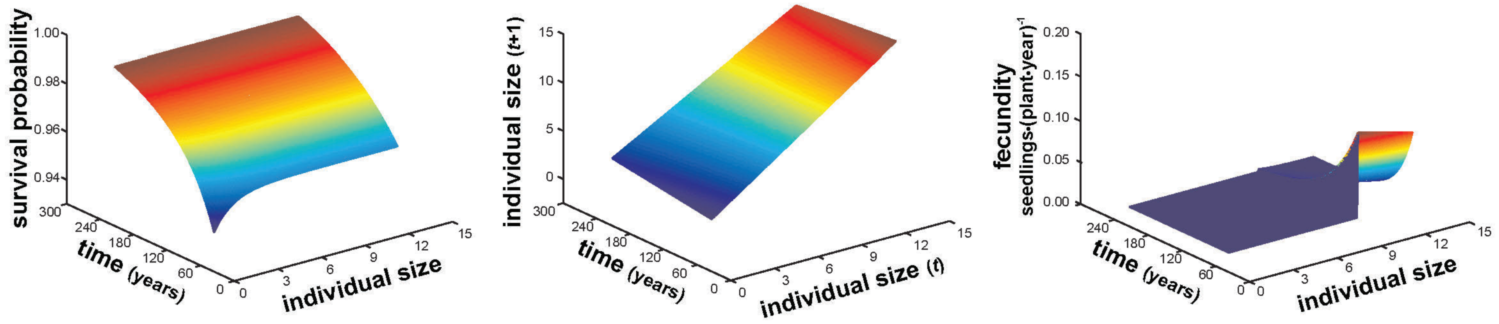

Appendix 5

Vital rates reconstructed for *Mammillaria dixanthocentron* (*cont.*)

w = 100

r_d = -0.62

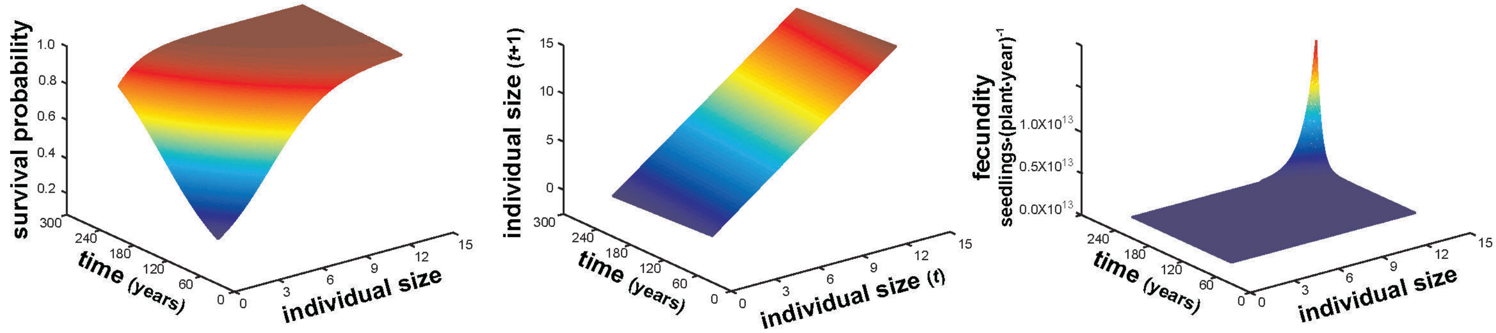

w = 1000

r_d = 0.82

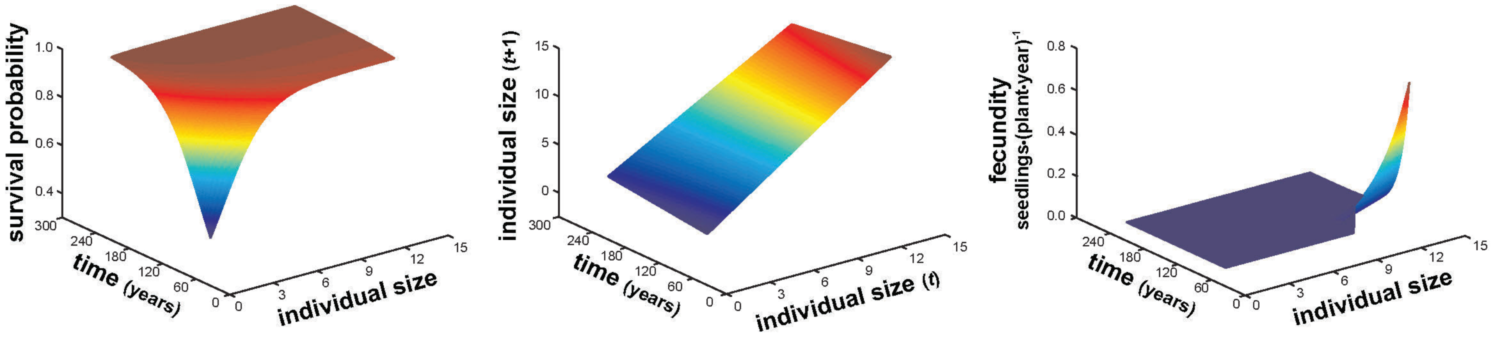

Supplement: Supplementary file 5 [file ece30003-2273-SD5.pdf]
